# Synthesis and Evaluation of New Coumarin Derivatives as Antioxidant, Antimicrobial, and Anti-Inflammatory Agents

**DOI:** 10.3390/molecules25143251

**Published:** 2020-07-16

**Authors:** Hanan M. Alshibl, Ebtehal S. Al-Abdullah, Mogedda E. Haiba, Hamad M. Alkahtani, Ghada E.A. Awad, Ahlam H. Mahmoud, Bassant M.M. Ibrahim, Ahmed Bari, Alexander Villinger

**Affiliations:** 1 Department of Pharmaceutical Chemistry, College of Pharmacy, King Saud University, Riyadh 11451, Saudi Arabia; ealabdullah@ksu.edu.sa (E.S.A.-A.); mogedda.haiba@yahoo.com (M.E.H.); ahamad@ksu.edu.sa (H.M.A.); abari@ksu.edu.sa (A.B.); 2 Department of Medicinal Chemistry, National Research Centre, Cairo 12622, Egypt; 3 Chemistry of Natural and Microbial Product Department, National Research Centre, Cairo 12622, Egypt; ghadaawadnrc@gmail.com; 4 Department of Therapeutic Chemistry, Pharmaceutical and Drug Industries Research Division, National Research Centre, Dokki, Cairo 12622, Egypt; ahlam@hotmail.co.uk; 5 Pharmacology Department, Medical Research Division, National Research Centre, Cairo 12622, Egypt; bmmih1974@gmail.com; 6 Institut für Chemie, Abteilung Anorganische Chemie, Universität Rostock, Albert-Einstein-Str. 3a, 18059 Rostock, Germany; alexander.villinger@uni-rostock.de

**Keywords:** anti-inflammatory, antioxidant, antimicrobial, coumarin, sulfonamide, pyranocoumarin, synthesis

## Abstract

New pyranocoumarin and coumarin-sulfonamide derivatives were prepared and evaluated for their antioxidant, antimicrobial, and/or anti-inflammatory activities. Coumarin-sulfonamide compounds **8a–d** demonstrated significant antioxidant activity, while **7c**,**d**, **8c**,**d**, and **9c**,**d** exhibited antimicrobial activity equal to or higher than the standard antimicrobials against at least one tested microorganism. Regarding the anti-inflammatory testing, pyranocoumarins **2b**, **3a**,**b** and **5c** and coumarin-sulfonamide compound **9a** showed more potent antiproteinase activity than aspirin in vitro; however, five compounds were as potent as aspirin. The anti-inflammatory activity of the promising compounds was further assessed pharmacologically on formaldehyde-induced rat paw oedema and showed significant inhibition of oedema. For in vitro COX-inhibitory activity of coumarin derivatives, pyranocoumarin derivative **5a** was the most selective (SI = 152) and coumarin-sulfonamide derivative **8d** was most active toward COX-2 isozyme. The most active derivatives met the in silico criteria for orally active drugs; thus, they may serve as promising candidates to develop more potent and highly efficient antioxidant, antimicrobial, and/or anti-inflammatory agents.

## 1. Introduction

Many diseases result from oxidative stress caused by the overproduction of reactive oxygen species (ROS) [1], which play an essential role in the pathogenesis of cardiovascular and neurodegenerative diseases as well as in tumour growth, and age-related disorders. Inflammatory diseases can affect the quality of life of many patients; however, the current medicinal drugs are not always effective and may cause serious adverse effects [2]. Nonetheless, infectious diseases are among the leading causes of death globally [3], and antimicrobial resistance has been commonly reported worldwide [3,4]. These obstacles necessitate the search for new therapies with potential antioxidant, anti-inflammatory, and antimicrobial activities.

**Figure 1:**
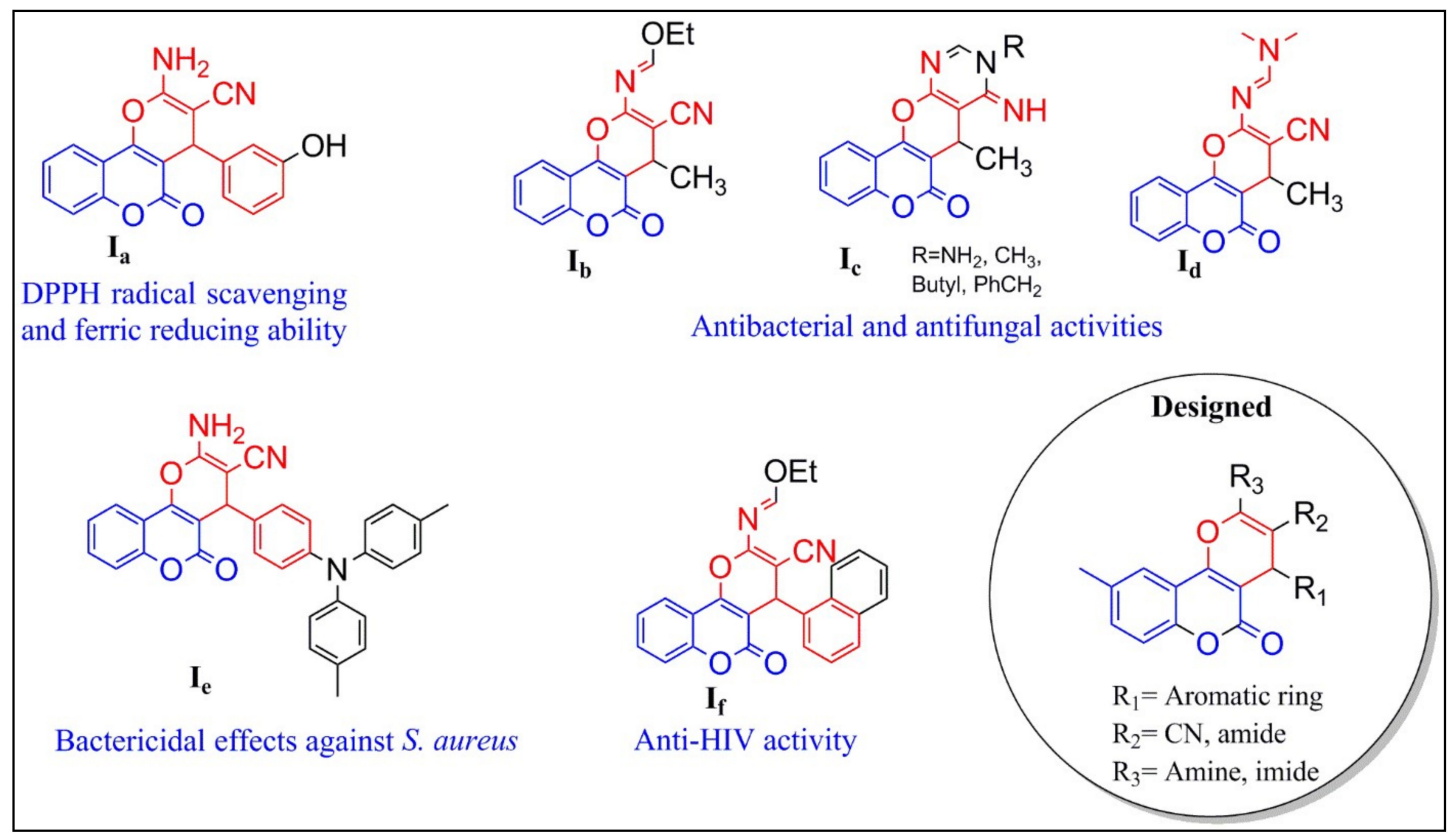
Bioactive pyranocoumarin derivatives and design of pyranocoumarins. DPPH, 2,2-diphenyl-1-picrylhydrazyl.

**Figure 2:**
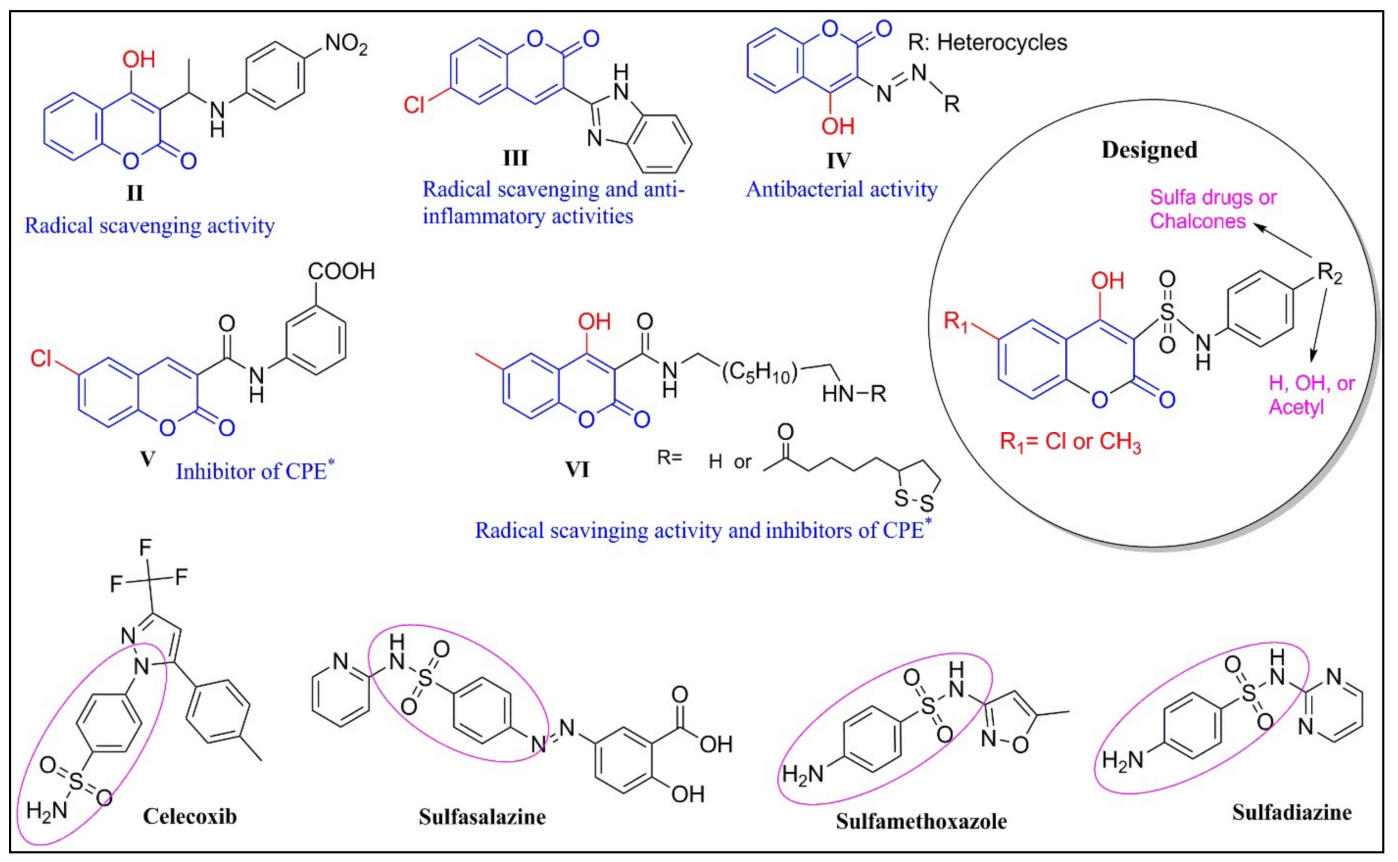
Bioactive coumarin derivatives and design of coumarin-sulfonamides. * CPE: Carrageenan-induced paw oedema.

Coumarin (2*H*-1-benzopyran-2-one) derivatives are a large class of highly important lactones containing a fused structure of a benzene ring and α-pyrone. Coumarin derivatives have increasingly appealed to medicinal chemists because of their potential role in preventing and treating diseases [5,6]. Numerous research reports have indicated hydroxycoumarin and its dihydropyran derivatives as potential candidates for the development of antioxidant (Figure 1 and Figure 2; **I_a_** [7], **II** [8], **III** [9], and **V** [10]), anti-inflammatory (**III**, **V**, and **VI** [11]), and antimicrobial (**I_b–d_** [12], **I_e_** [13], **I_f_** [14], and **IV** [15]) agents. Therefore, they are considered as a compelling starting point for a wide range of applications in the medicinal field.

Despite massive efforts by various global academic and industrial research laboratories over the years, developing antioxidant, anti-inflammatory, and antimicrobial agents that are potent, safe, and selective remains quite challenging. On one hand, coumarin derivatives represent a broad spectrum of biological activities, which may be useful in developing new compounds with the aforementioned desired activities [16]. On the other hand, sulfonamides have become an intriguing scaffold due to their broad medicinal applicability. Several sulfonamides are clinically used as therapeutic agents, such as the anti-inflammatory agents celecoxib [17] and sulfasalazine [18] and the antimicrobial agents sulfamethoxazole [19] and sulfadiazine [20] (Figure 2). Thus, they have been considered as promising molecules for the design and development of new drugs. 

Previous reports have suggested that beginning with modification of compounds known to have pharmacological effects (pyranocoumarins, Figure 1) or conjugating two structures with promising biological effects into an interesting motif (coumarin-3-sulfonamides, Figure 2) may be useful for developing new effective therapeutic agents. Therefore, this work aimed to design and synthesise new coumarin derivatives [21], including pyranocoumarins and coumarin-sulfonamides, in order to evaluate their antioxidant, anti-inflammatory, and antimicrobial activities against medically important bacterial and fungal strains. In this study, 4-hydroxy-6-methylcoumarin and 6-chloro-4-hydroxycoumarin moieties were selected as pharmacophores with the aim of obtaining compounds with more powerful desired effects. In addition, in silico assessment of the drug-likeness of the target compounds was performed.

## 2. Results and Discussion

### 2.1. Chemistry

**Scheme 1:**
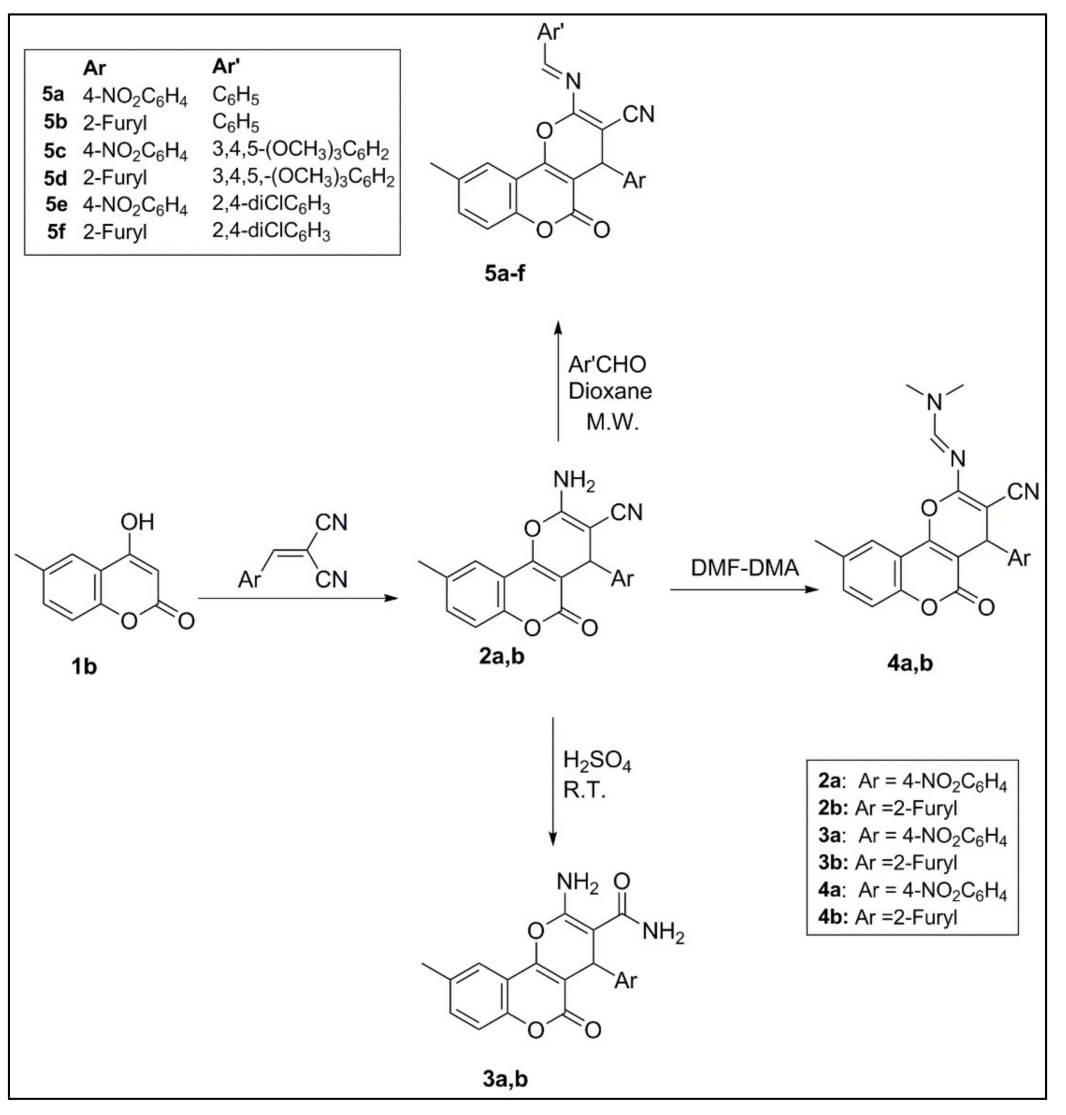
Synthetic routes of pyranocoumarins **2–5**. DMF-DMA, *N,N*-dimethylformamide-dimethylacetal; M.W., microwave; R.T., room temperature.

**Figure 3:**
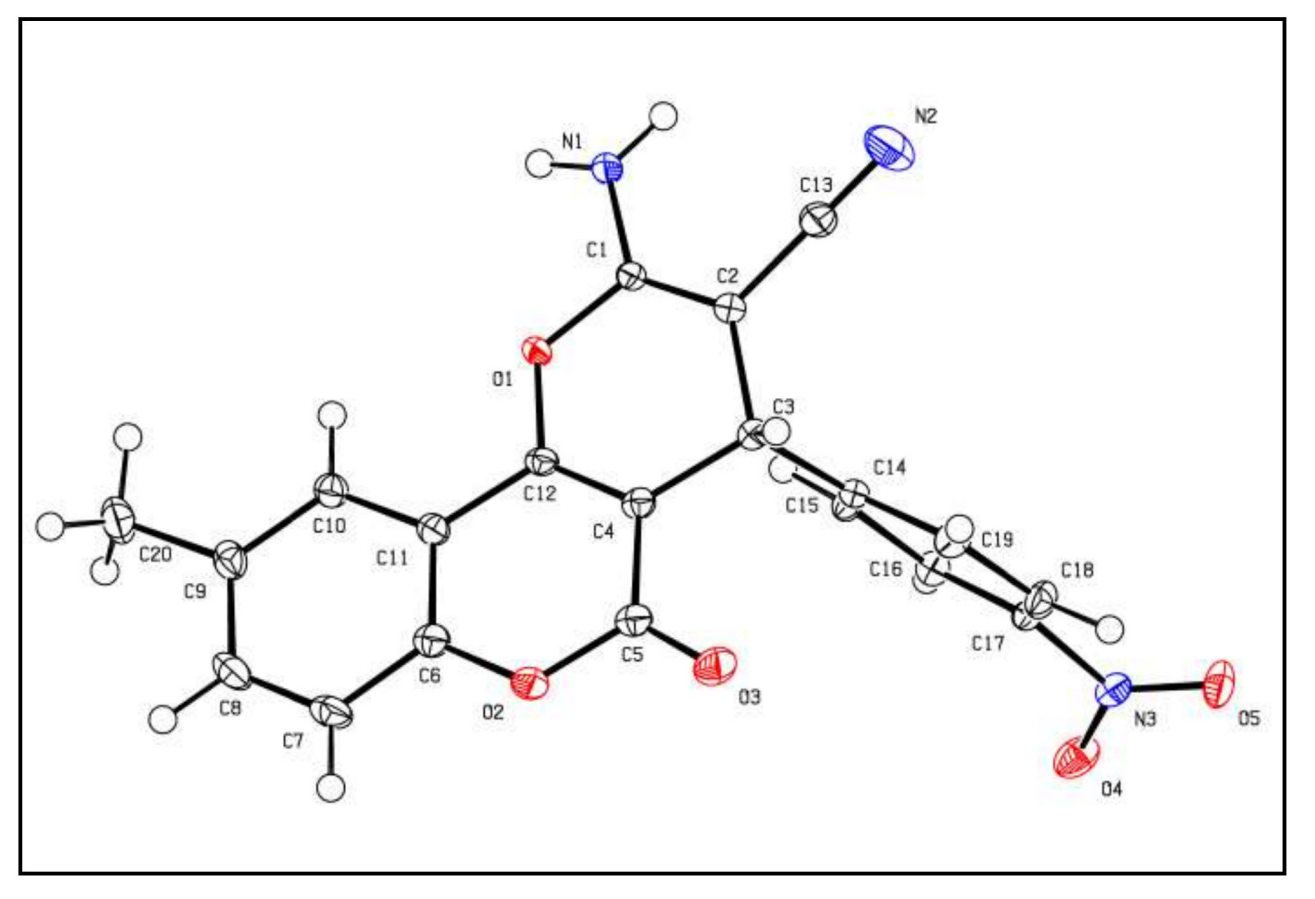
X-ray crystal structure of **2a**.

**Scheme 2:**
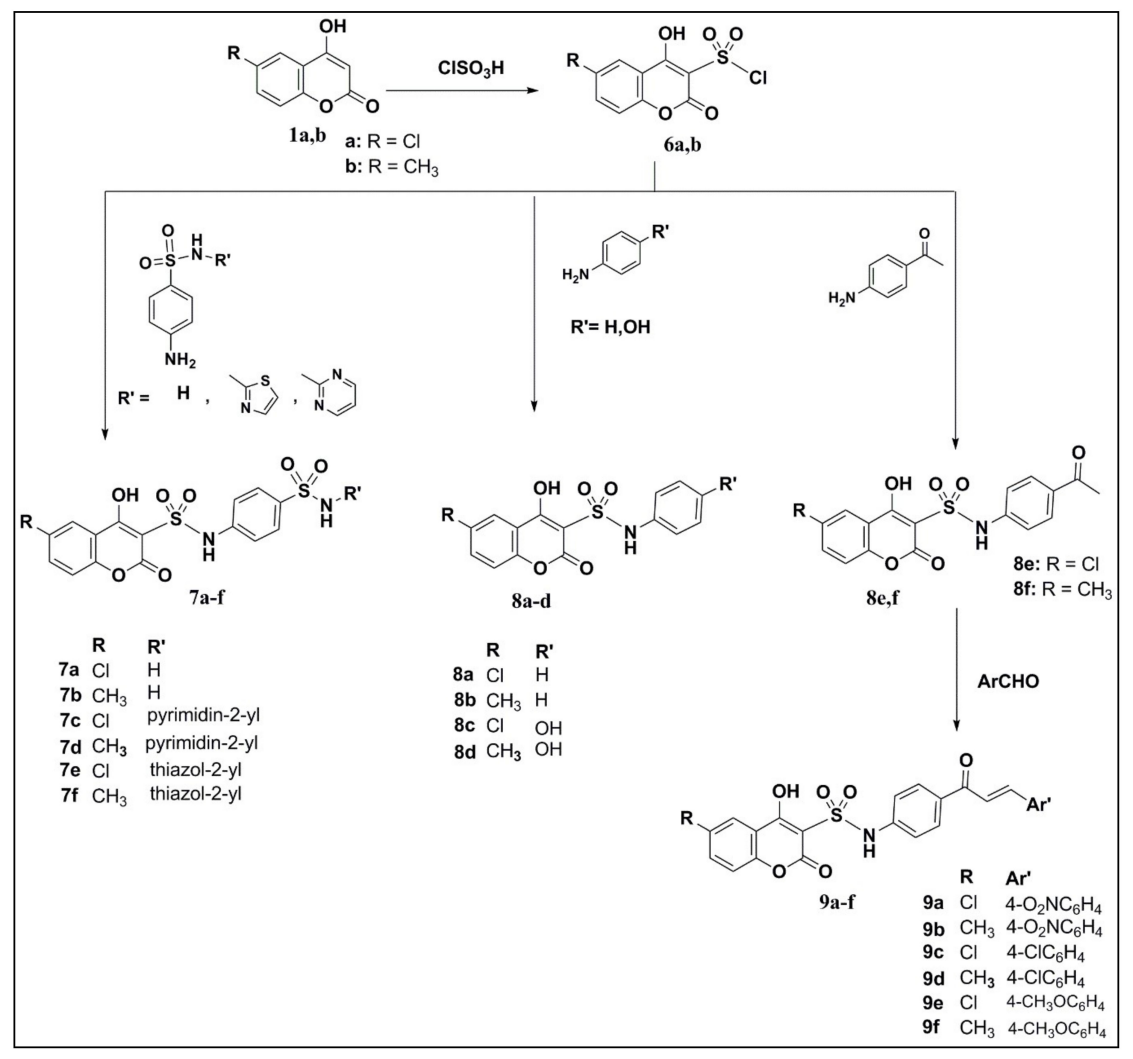
Synthetic routes of coumarin-sulfonamide derivatives **6–9**.

In this work, facile and green chemistry methods were used for synthesising new coumarin derivatives via the routes illustrated in Scheme 1 and Scheme 2. Synthetic Scheme 1 shows the procedure for pyranocoumarin preparation. All of these derivatives have a chiral centre at Position 4, which are confirmed by one and, in some cases, two dimensional NMR spectroscopy and supported by the X-ray analysis of **2a**. These results suggest the presence of these compounds as racemic mixtures of (±)-enantiomers. High yields of the new pyranocoumarins **2a,b** were prepared with one-pot reaction using water as a solvent by the reaction of different aryl aldehydes with malononitrile and 4-hydroxy-6-methylcoumarin, catalysed by potassium hydrogen phthalate (KHP) to generate the desired carbonitriles according to a previously reported method [22]. The X-ray crystal structure of compound **2a** was studied at 123 K (Figure 3). In the crystal structure, NH protons were refined freely. Moreover, the nitrobenzene moiety in Position 4 is perpendicular to the pyranocoumarin moiety. This compound has chirality at C-3 and was crystallised in a space group P 21/n with a chiral monoclinic centrosymmetric arrangement confirming its presence as a racemic mixture (R/S). The tentative assignments are done on the basis of geometric and atom type data in the cif. The full crystallographic data are included in the Supplementary Materials (Table S1).

The carboxamide derivatives **3a** and **3b** were obtained in good yields through acidic hydrolysis of **2a** or **2b** with sulphuric acid, at room temperature. The reaction of **2a,b** with excess *N,N*-dimethylformamide-dimethylacetal (DMF-DMA) at 100 °C yielded the corresponding derivatives **4a,b** within 15–40 min. Microwave irradiation was used to obtain the target derivatives **5a–f** by treating **2a,b** with different aromatic aldehydes in 1,4-dioxane. Several pilot experiments were carried out to optimise the irradiation time using the Biotage Initiator+ microwave (400 W). It was revealed that microwave irradiation for 90–110 min was optimal for this reaction. In this method, the products were obtained with a short reaction time without using hazardous catalysts; however, all compounds had low yields (6–37%).

Synthetic Scheme 2 shows the procedure for preparing coumarin-3-sulfonamide compounds. Coumarin sulfonyl chlorides **6a,b** were produced with excellent yields (≥97%) by treating 4-hydroxy-6-substituted coumarin **1a** or **1b** with chlorosulfonic acid in dichloromethane at 0 °C. Despite the limited available information regarding coumarin-3-sulfonamides, they were generally obtained either by using a Knoevenagel condensation reaction [23] or through the condensation of coumarin-3-sulfonyl chlorides with aromatic amines [24]. In the present work, the coumarin-3-sulfonamides **7a–f** were prepared by refluxing an equimolar quantity of **6a** or **6b** and different sulfa-drugs in absolute ethanol. However, the reaction of **6a** or **6b** with nucleophilic reagents (1:2), aniline or *p*-substituted aniline, in absolute ethanol under reflux involves the formation of the corresponding coumarin-3-sulfonamides **8a–f** with yields of 48–92%. Coumarin-sulfonamide chalcone derivatives **9a–f** were synthesised in low yields (15–35%) by Claisen–Schmidt condensation of *p*-acetyl derivatives of 4-hydroxy-6-(substituted)coumarin-3-sulfonamides **8e** or **8f** with the *p*-substituted aromatic aldehyde through adding sodium hydroxide solution in ethanol at room temperature.

### 2.2. Antioxidant Testing

The antioxidant effects of the synthesised compounds were assessed using the 2,2-diphenyl-1-picrylhydrazyl (DPPH) antioxidant assay [25,26]. Table 1 summarises the antioxidant effects of the test samples and ascorbic acid except for compound **5b** because it was produced in poor yield. Fourteen compounds exhibited free radical scavenging activity; however, all compounds showing antioxidant activity were dose-dependent. 

Among the pyranocoumarins, only compounds **3b** and **5d** showed modest antioxidant activity, with IC_50_ values of 48.38 ± 4.61 and 82.92 ± 3.30 μg/mL, respectively. Similarly, the activities of the coumarin-sulfonamide derivatives **7a–f** were comparable to those of compounds **3b** and **5d** with IC_50_ values in the range of 61–120 μg/mL. However, antioxidant activity was significantly improved in compounds **8a–f** (IC_50_ = ~4–35 μg/mL), of which **8c**,**d** were the most potent. The coumarin-sulfonamide chalcones **9a–f** demonstrated no antioxidant activity, with IC_50_ values >200 μg/mL.

Regarding the newly synthesised coumarin-3-sulfonamides, generally, the chloro-substituted compounds at Position 6 of the coumarin ring showed higher radical scavenging potential than the methyl-substituted derivatives. Interestingly, compounds **8c**,**d** exhibited the highest antioxidant activity; this improved activity might be explained by the presence of the phenolic hydroxyl group. In addition, compounds **8a**,**b**,**e**,**f** showed good radical scavenging activities when compared to ascorbic acid. The sulfathiazole-containing compounds **7e**,**f** and sulphanilamide-containing compounds **7a**,**b** showed higher antioxidant activity than that of sulfadiazine-substituted compounds **7c**,**d**.

### 2.3. Antimicrobial Testing

The new molecules were individually tested for their antimicrobial activity in vitro against standard bacteria strains from the American Type Culture Collection (ATCC), namely, *Staphylococcus aureus* ATCC 29213, *Bacillus subtilis* ATCC 6633*, Bacillus megaterium* ATCC 9885 (Gram-positive), *Escherichia coli* ATCC 2592, and *Pseudomonas aeruginosa* ATCC 27,953 (Gram-negative). The compounds were also microbiologically tested against locally isolated *Saccharomyces cerevisiae* (yeast) and the standard Agricultural Research Service Culture Collection (NRRL) strain of the yeast-like pathogenic fungus *Candida albicans* NRRL Y-477. Primary screening was conducted using the agar well diffusion method [27] using nutrient agar (NA) medium and Sabourand dextrose agar (SDA) medium for the tested pathological bacteria and yeast, respectively. The minimal inhibitory concentration (MIC) for the most active compounds (having inhibition zones (IZ) > 16 mm) against the same microorganisms used in the primary screening was evaluated using the two-fold serial dilution technique with the proper nutrient broth [28]. 

Table 2 represents the preliminary antimicrobial testing results using the agar well diffusion method. The compounds showed varying degrees of inhibition against these microorganisms. Generally, potent antimicrobial effects were observed with compounds **2a,b**, **5a**,**c**,**f**, **7a**,**c**,**d–f**, **8a**,**c**,**d**, and **9b–f** with IZs larger than 25 mm against at least one tested microorganism. However, molecules **5e** and **8b** were moderately active with IZs between 20 and 24 mm, whereas **3a**,**b**, **4a**,**b**, **5d**, **8e**,**f**, and **9a** showed marginal activity (IZs of 15–19 mm). Contrarily, only molecule **7b** showed no inhibitory activity against the tested microorganisms (IZ <15 mm).

The MICs of the active molecules (IZs >16 mm) are summarised in Table 3. Generally, compounds **7c**,**d**, **8c**,**d**, and **9c**,**d** which showed the highest IZs, also exhibited potent antimicrobial activity against one or more tested microorganisms when compared to standards, with MICs of 125 μg/mL. Interestingly, compound **7d** demonstrated broad-spectrum activity against all microorganisms with a 125 μg/mL MIC.

Microbiological screening showed that the newly synthesised pyranocoumarins exhibited variable antimicrobial effects against Gram-positive and Gram-negative bacteria and fungi. These compounds were generally more sensitive toward Gram-positive bacteria. However, only compounds **5a** and **5c**, which contained unsubstituted benzylidene and 3,4,5-trimethoxybenzylidene, respectively, displayed strong antibacterial activity against Gram-negative bacteria (IZ: 24–26 mm) with strong-to-moderate inhibitory activity against Gram-positive bacteria (22–27 mm). Similarly, compounds **2a**,**b** and **5f** showed strong-to-moderate antibacterial effects against Gram-positive bacteria (20–25 mm), with MICs in the range of 500–1000 μg/mL. Amongst the entire series, only compound **5a**, which contains an unsubstituted benzylidene, possessed strong antimicrobial activity against most tested microorganisms (22–27 mm) with MICs of 250–500 μg/mL, as compared to the control drugs. In addition, strong antimicrobial activity was observed against *S. cerevisiae* in compounds **2a** (25 mm), **2b** (27 mm), and **5c** (27 mm), with a 500 μg/mL MIC.

Most of the newly synthesised coumarin-3-sulfonamides exhibited strong-to-moderate antimicrobial activity with respect to the reference drugs. Generally, most of these molecules demonstrated broad-spectrum antimicrobial activity. In addition, compounds **7a**,**c**,**d**, **8a**,**c**,**d**, and **9c,d** exhibited antimicrobial activity against at least one microorganism that was equal to or higher than those of the standard antimicrobials ciprofloxacin and ketoconazole.

Samples **7c**,**d**, and **8c**,**d** which contain sulfadiazine or 4-hydroxyphenyl moieties, exhibited slightly higher antibacterial activity (30 mm) than that of standard ciprofloxacin (28 mm) against *S. aureus*, with an MIC of 125 μg/mL. Meanwhile, compounds **7a**, and **9c**,**d** which contain sulfanilamide or 4-chlorophenyl moieties, displayed antibacterial activity equal to that of ciprofloxacin (28 mm) against *S. aureus*, with a 250 μg/mL MIC. The antibacterial activity against *B. subtilis* by compounds **7c**,**d** which are substituted with sulfadiazine, was higher than that of the reference drug (30 mm) by one unit, whereas the same activity of compounds **7a**, **8c**,**d**, and **9c**,**d** was lower by one unit. Compounds **7c**,**d**, and **9c** showed antibacterial activity against *B. megaterium* equal to that of ciprofloxacin (30 mm). On the other hand, only compounds **8d** and **9d**, containing a methyl moiety along with 4-hydroxyphenyl or 4-chlorophenyl, respectively, exhibited higher antibacterial activity than ciprofloxacin (30 mm) against *E. coli* by one and eight units, respectively. Meanwhile, compounds **7c** and **9c**, containing a chloro moiety along with sulfadiazine or 4-chlorophenyl, respectively, possessed antibacterial potential against *E. coli* similar to that of the control drug. Only compound **8d**, containing a methyl moiety and 4-hydroxyphenyl, exerted antibacterial activity (31 mm) against *P. aeruginosa* higher than that of the standard drug, whereas **9c** showed activity equal to the standard (30 mm).

The antimicrobial activity against *S. cerevisiae* of compounds **7c**,**d**, **8c**,**d**, and **9c**,**d** showed IZ diameters of 31 or 32 mm, which was slightly higher than that of the control drug, ketoconazole (30 mm). In addition, compounds **7d**, **8a**,**c**,**d**, and **9c**,**d** exhibited antifungal activity against *C. albicans* that was two units higher than that of the reference drug (28 mm).

Lipophilicity is considered one of the most important properties in the optimisation process. Moreover, lipophilicity affects not only the pharmacodynamic profile of the drug, but also its pharmacokinetics [29]. However, increasing the lipophilicity of a compound facilitates microbial cell penetration, hence improving antimicrobial activity [30,31]. In fact, only compound **7b** was inactive against all tested microorganisms and showed the lowest consensus Log *P-*value (1.05) amongst all tested compounds, which may be attributed to its low Log *P-*value. Meanwhile, the remaining compounds, which showed mild-to-strong activity against one or more microorganism, have consensus Log *P-*values in the range of 1.23–5.33. However, lipophilicity is not the only factor that influences biological activity; other factors such as electronic effects and steric properties may also contribute to the biological effects of these compounds.

### 2.4. Anti-Inflammatory Activity

The newly synthesised compounds were individually tested for their in vitro anti-inflammatory activity against proteinase enzyme [32]. The most active compounds, **2b, 3a**, **7f**, and **8c**, were tested further in vivo for their acute anti-inflammatory activity using the formaldehyde-induced paw oedema method in rats [33]. This test was performed at the Pharmacology Department, Medical Research Division, National Research Centre, Cairo, Egypt. Furthermore, compounds **2–8** were evaluated for their COX1/COX2 inhibitory activities.

#### 2.4.1. Proteinase-Inhibitory Activity

Proteinases play a significant role in inflammatory reactions; in particular, they can contribute to arthritic reactions. Proteinases mainly exist in the lysosomal granules of neutrophils [34]. However, proteinase inhibitors were reported to exhibit significant levels of protection against tissue damage and proteolytic activity of neutrophils during inflammatory reactions [34,35,36]. 

The proteinase-inhibitory activities of the newly synthesised compounds are summarised in Table 4. The inhibition of proteinase activity at 250 µg/mL was found to be highest in compounds **3a** and **2b**, with 79.72 ± 4.51% and 74.68 ± 3.01% inhibition, respectively. Next, compounds **5c** and **9a** exhibited percent inhibition values of 68.5 ± 6.23% and 62.14 ± 4.87%, respectively. Compounds **3b**, **5d**,**e**, **7f**, and **8a**,**c** showed proteinase-inhibitory activity with 41–49% inhibition. Meanwhile, compounds **4a**,**b**, **7d**, and **9d** showed modest proteinase-inhibitory activity, with percent inhibition values of 31–36%. The remaining compounds showed weak antiproteinase activity with percent inhibition values below 30%. Compound **9b**, however, showed no antiproteinase activity at the tested concentration.

The pyranocoumarins **2b**, **3a**,**b**, and **5c** showed more potent antiproteinase activity than did standard aspirin (45.83 ± 4.21%), whereas compounds **5d**,**e** were comparable to the standard drug. The remaining pyranocoumarins showed moderate-to-poor activity. Compound **2a**, containing 4-nitrophenyl, showed weak antiproteinase activity, while the replacement of a cyano group with carboxamide (**3a**) significantly enhanced this activity. In addition, replacement of the protons of the amino group with 3,4,5-trimethoxybenzylidene or 2,4-dichlorobenzylidene (**5c** and **5e**, respectively) significantly enhanced the proteinase-inhibitory activity, indicating that the presence of a separate 4-nitrophenyl in pyranocoumarin does not contribute to significant antiproteinase activity, but its presence along with carboxamide, 3,4,5-trimethoxybenzylidene, or 2,4-dichlorobenzylidene does aid such activity. In contrast, compound **2b**, which contains furan-2-yl, showed significant antiproteinase activity; however, hydrolysis of the cyano group resulting in carboxamide (**3b**) or the replacement of amino group protons with 3,4,5-trimethoxybenzylidene or 2,4-dichlorobenzylidene (**5d,f**) reduced the activity by more than 25%. Compounds **4a**,**b** exhibited modest antiproteinase activity. This is probably due to the presence of *N,N*-dimethyl formimidamide at Position 2 of the pyrano ring.

Regarding the newly synthesised coumarin-3-sulfonamides, only compound **9a** exhibited higher antiproteinase activity than aspirin due to the presence of the chloro group at Position 6 of the coumarin ring along with 4-nitrophenyl in the chalcone moiety. Conversely, replacement of this chloro substituent with a methyl group diminished the antiproteinase activity. Meanwhile, compounds **7f** and **8a**,**c** possess activity comparable to that of standard aspirin. The remaining compounds of this series exhibited modest-to-poor antiproteinase activity. The presence of a methyl moiety at Position 6 along with pyrimidine or thiazole rings in compounds **7d**,**f** was essential to their antiproteinase activity. In contrast, this activity was significantly reduced by replacing the methyl moiety with a chloro moiety or replacing the rings with hydrogen. Compounds **8e**,**f** showed poor antiproteinase activity due to the presence of 4-acetylphenyl at Position 3 of the coumarin ring. Meanwhile, compounds **8a**,**c** showed good activity that could be due to the presence of the chloro moiety and phenyl or 4-hydroxyphenyl at Positions 6 and 3, respectively, of the coumarin ring. In contrast, replacement of the chloro moiety with methyl reduced the activity significantly. Compounds **9e,f** exhibited weak antiproteinase activity that might be due to the presence of 4-methoxyphenyl in the chalcone moiety. Compound **9d**, which contains methyl and 4-chlorophenyl, showed modest antiproteinase activity, whereas compound **9c**, containing chloro and 4-chlorophenyl, exhibited poor activity.

#### 2.4.2. Pharmacological In Vivo Study

Prophylactic anti-inflammatory effect on formaldehyde-induced rat paw oedema:

Based on the results of the antiproteinase activity, the most effective compounds, **2b** and **3a**, and the moderately effective ones, **7f** and **8c**, were selected for their in vivo evaluation of anti-inflammatory activity.

**Figure 4:**
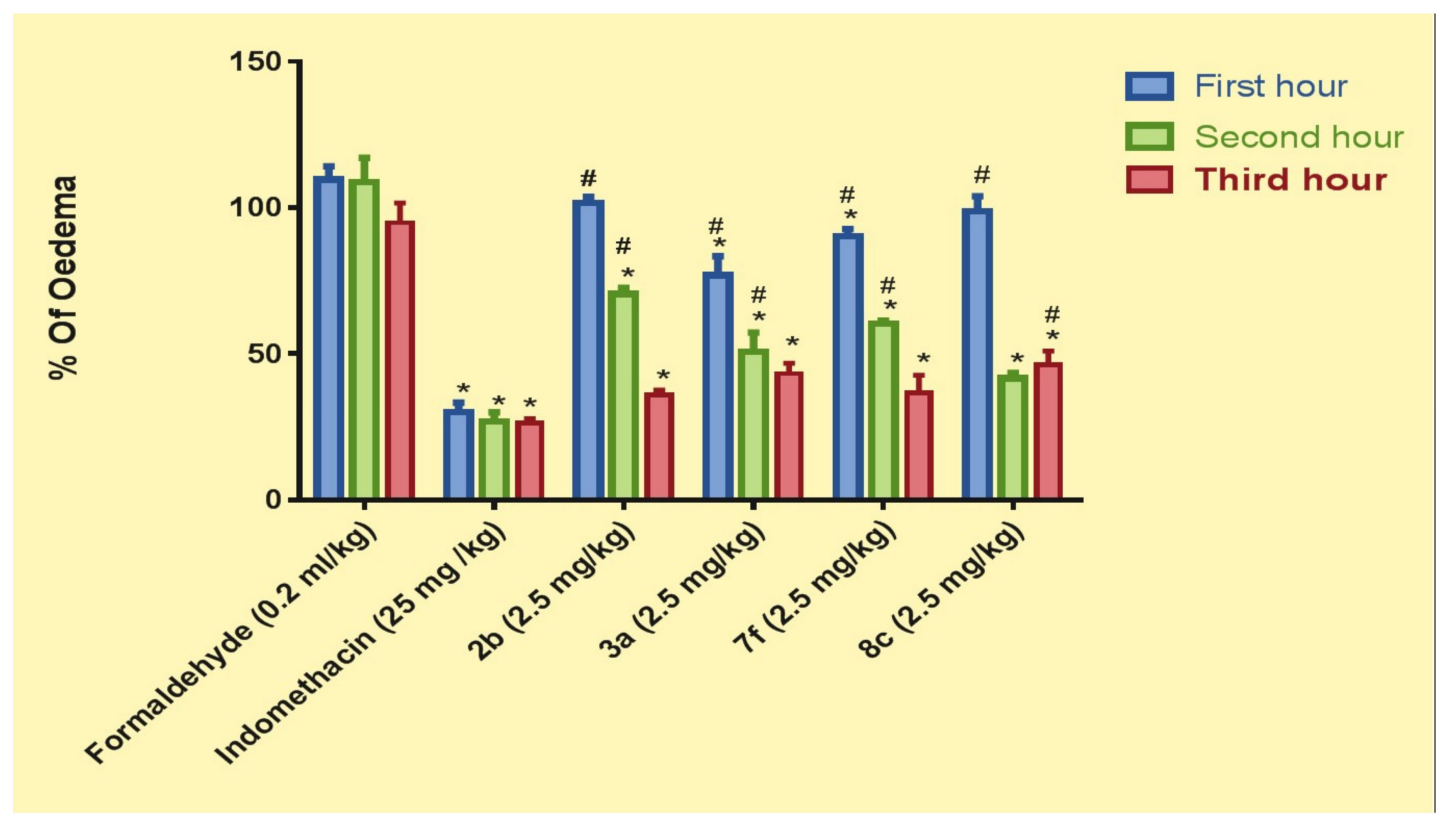
Percent of oedema in rats pretreated with indomethacin (25 mg/kg) and compounds **2b**, **3a**, **7f**, and **8c** (2.5 mg/kg). Results are expressed as means ± SE, n = 6. Results are statistically significant at *p* < 0.0001 (* vs. formaldehyde, # vs. indomethacin).

In the present study, when rat paw volumes were consecutively measured 1, 2, and 3 h after subplantar injection of 0.2 mL (1%, *w*/*v*) formaldehyde in the positive control group, it was found that formaldehyde significantly increased paw volumes by 109.6, 108.7, and 94.89%, respectively, of their basal volumes (Figure 4).

When used orally as a reference drug for coumarins, indomethacin (25 mg/kg) significantly inhibited inflammation, as evidenced by paw volume reduced by 72.09, 74.79, and 71.5% compared to the positive control group after 1, 2, and 3 h, respectively, following subplantar injection of 0.2 mL (1% *w*/*v*) formaldehyde.

**Figure 5:**
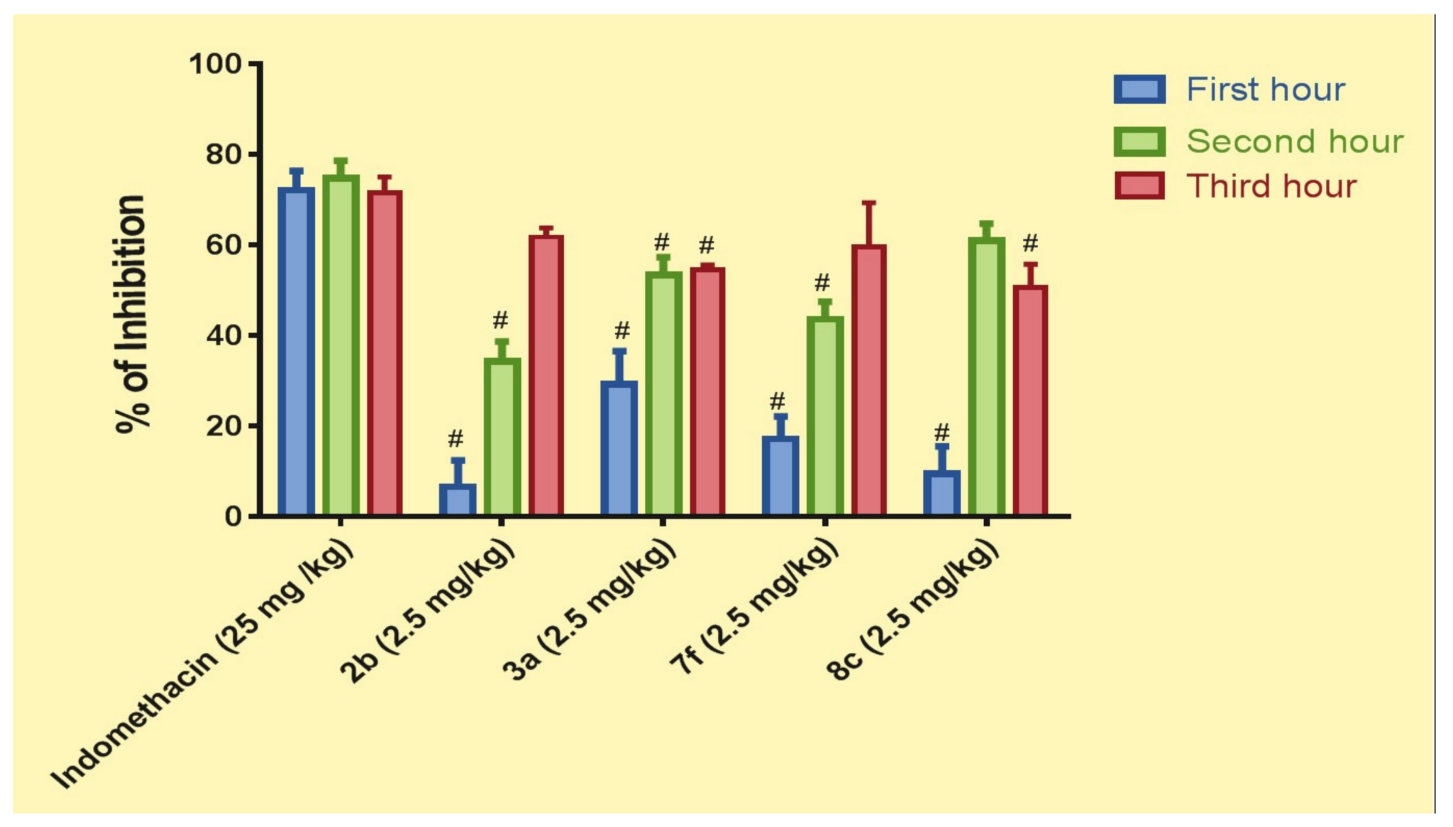
Percent inhibition of the standard drug indomethacin (25 mg/kg) and compounds **2b**, **3a**, **7f**, and **8c** (2.5 mg/kg) on rat paw oedema induced by formaldehyde (0.2 mL/kg). Results are expressed as means ± SE, n = 6. Results are statistically significant at *p* < 0.0001 (# vs. indomethacin).

Regarding the anti-inflammatory effects of coumarins **2b**, **3a**, **7f**, and **8c**, all compounds produced significant protection against inflammation throughout the experiment duration (Figure 5). Compound **3a** showed significantly better inhibition than others (29.2% inhibition) only 1 h after inducing inflammation, followed by compound **7f** (17.18% inhibition), **8c** (9.45%) and **2b** (6.57%). However, **3a** was less effective than indomethacin; which is likely due to administering these compounds at one-tenth of the indomethacin concentration. Moreover, the anti-inflammatory effects of the compounds were significantly increased after 2 and 3 h when compared to the positive control group. 

#### 2.4.3. COX Inhibition

Compounds **2–8** were further tested for their inhibitory activities against COX-1 and COX-2 using human assay kits. The COX-inhibitory effects of these compounds and the standards indomethacin and celecoxib are summarised in Table 5. All compounds were screened for their COX-inhibitory activity except compounds **9a–f** because they were obtained in low yield. The selectivity index (ratio of COX-1/COX-2) of each compound was determined and compared with that of celecoxib, a selective COX-2 inhibitor. 

Compared to indomethacin, all of these molecules were generally less active against COX-1 isozyme and more active toward COX-2. However, potent COX-2-inhibitory activity (IC_50_ ≤ 10μM) was observed in most pyranocoumarin derivatives (**2a**,**b**, **3a**,**b**, **4a**, and **5a**,**e**,**f**) and six coumarin-sulfonamide derivatives (**7b**,**d**,**f**, and **8a**,**d**,**e**). Interestingly, compound **5a** showed significant selectivity toward COX-2 isozyme, which was highest amongst the tested compounds (SI = 152) compared to the standard celecoxib (SI = 243.9). Compounds **2a**,**b** and **7d** followed with SI values of 64, 47.3, and 42.6, respectively. 

Regarding the pyranocoumarins, hydrolysis of the cyano group at Position 3 to an amide or replacement of the amino group at Position 2 with *N,N*-dimethyl formimidamide was found to reduce the selectivity toward COX-2 isozyme (**2a**,**b** vs. **3a**,**b** and **4a**,**b**). Substitution with 3,4,5-trimethoxybenzylidene or 2,4-dichlorobenzylidene in Position 2 was found to decrease the selectivity as well (**5c–f**). Notably, the presence of unsubstituted benzylidene along with the nitrophenyl at Position 4 improved the selectivity by about 3.6-fold (**2a**, SI = 42 vs. **5a**, SI = 152), whereas a furyl ring at the same position significantly reduced the selectivity (**5b**, SI = 10.9).

For coumarin-sulfonamides, the presence of a methyl moiety at Position 6 of the coumarin ring and pyrimidin-2-yl in Position 3 was shown to improve COX-2 selectivity compared to the remaining derivatives of this series (**7d**, SI = 64 vs. **7a–c** and **7e–f**, SI = 1.8–13.7). However, compound **8f**, which contains methyl and acetyl moieties, was the only derivative that showed relatively selective COX-1 activity (SI = 0.3), and replacing the methyl with a chloro moiety changed it into a selective COX-2 compound (**8e**, SI = 2.2). Amongst all tested compounds, **8d**, containing methyl and hydroxyl moieties, demonstrated the most potent activity against COX-2 isozyme (IC_50_ = 1.86, SI = 10.5); this potency and selectivity were reduced nearly ten-fold by replacing the methyl with a chloro moiety (**8c**, IC_50_ = 11.9, SI = 1.3).

### 2.5. In Silico Studies

#### 2.5.1. Docking Studies

The major difference in the COX-2 active site compared to that of COX-1 is the presence of Val 523 instead of Ile 523. As a much smaller amino acid, Val 523 causes a conformational change in Tyr 355, thus producing a side pocket in COX-2 [37,38]. In addition, it has been reported that His 90, Gln 192, and Tyr 355 control the access of ligands to this pocket in COX-2 [37,38].

**Figure 6:**
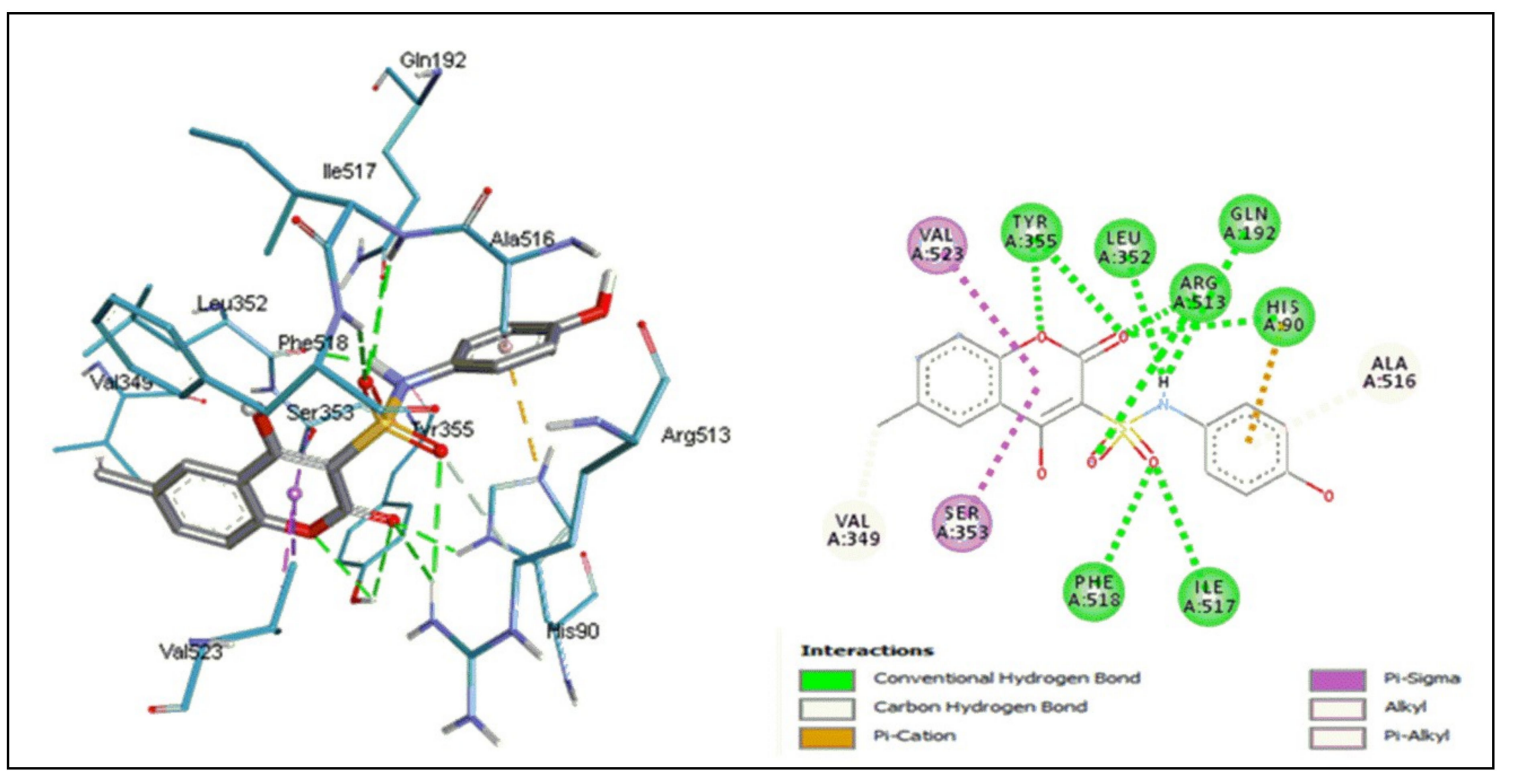
Binding interactions between **8d** and human COX-2 enzyme.

**Figure 7:**
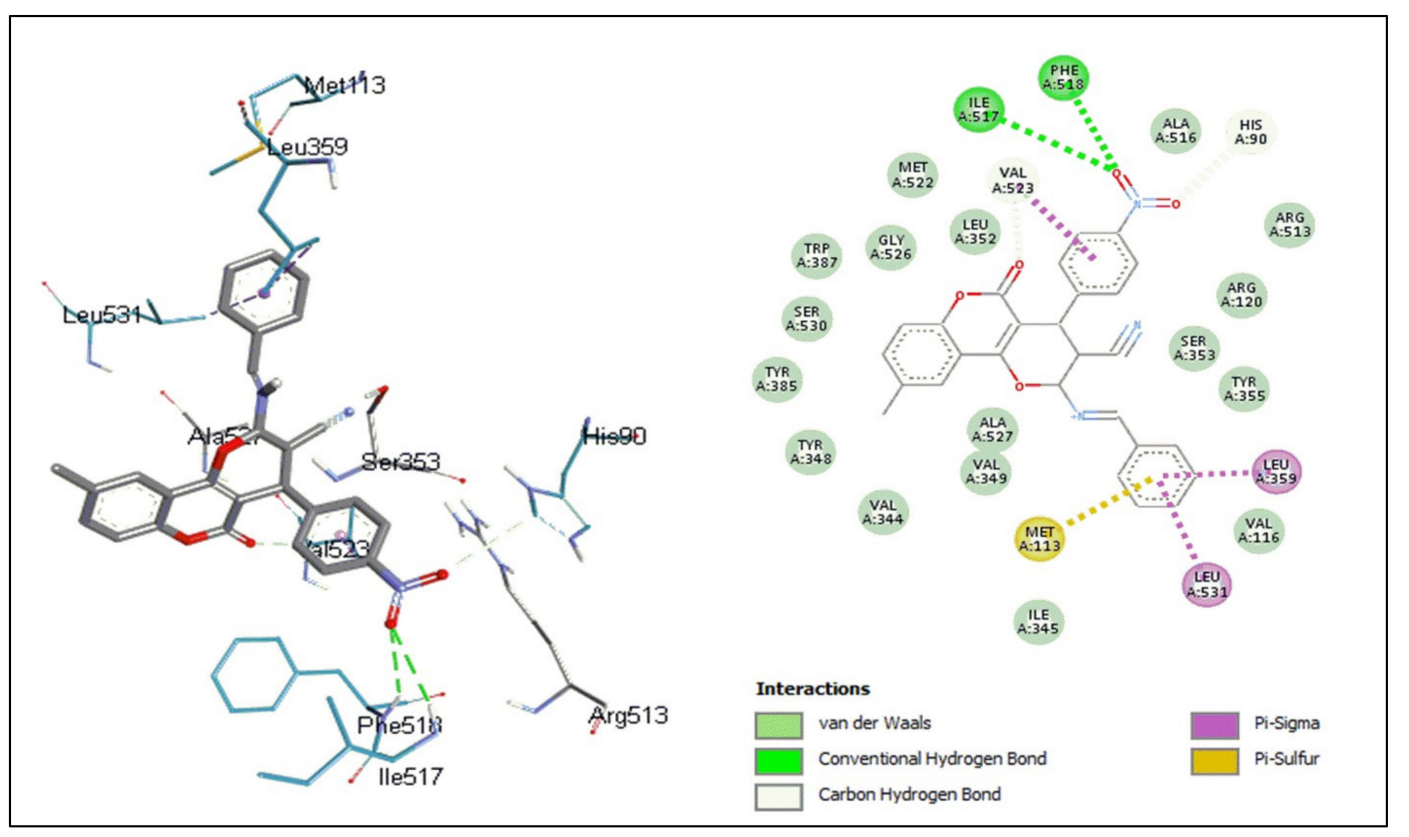
Binding interactions between **5a** and human COX-2 enzyme.

Molecular docking was studied to predict the intermolecular interactions of compounds **5a** and **8d** with human COX-2 isozyme (PDB ID: 5KIR, Figure 6 and Figure 7). The docking simulation shows that the binding affinity of the coumarin-sulfonamide derivative **8d** (−7.3 kcal/mol) was better than that of the pyranocoumarin derivative **5a** (−4.4 kcal/mol). These results are consistent with the in vitro assay findings. 

Compound **8d** formed eight hydrogen bonds with the binding site. Oxygen atoms of the coumarin ring and sulfonamide serve as the acceptors of hydrogen bonds with Typ 355, Arg 513, Phe 518, Ile 517, and His 90. However, the amino group of the sulfonamide acted as a hydrogen bond donor with Leu 352 and Gln 192. The Val 523 residue formed a Pi-sigma interaction with the pyrone ring of coumarin; furthermore, other weaker bonds, such as carbon hydrogen, Pi-cation, Pi-sigma, alkyl, and Pi-alkyl bonds, also accounted for the binding affinity of this compound. 

Meanwhile, compound **5a** showed only two hydrogen bonds with Ile 517 and Phe 518, and the oxygen atom of the nitro group served as the hydrogen bond acceptor. Moreover, the Val 523 residue formed a Pi-sigma interaction with the nitrobenzyl ring of this derivative. Other interactions, including van der Waals, carbon hydrogen, Pi-sigma, and Pi-sulphur bonds, formed between **5a** and other binding site residues. The lower number of hydrogen bonds could contribute to a lower binding affinity as compared with **8d**.

#### 2.5.2. Drug-Likeness Assessment

In order to study the drug-likeness of the newly synthesised compounds, in silico Lipinski’s Rule of Five (RO5) and topological polar surface area (TPSA) analyses were conducted using Molinspiration [39]. About 90% of orally active compounds that reach phase II clinical trials or higher satisfy RO5 [40]. This rule states that a molecule likely to be developed as an orally active drug candidate should not show more than one violation of the following four criteria: Molecular weight ≤ 500, octanol-water coefficient (LogP) ≤ 5, H-bond donors (n-OHNH) ≤ 5, and H-bond acceptors (n-ON) ≤ 10 [40]. Despite the importance of RO5 for labelling a molecule as “drug-like”, these criteria are restricted to the topic of oral bioavailability via passive transport alone. Moreover, TPSA is another major factor for predicting the oral availability of compounds, and its values for intestinal absorption should be less than 140 Å^2^ [41].

Table 6 shows that all compounds have no more than four hydrogen bond donors, and most compounds have 10 or fewer hydrogen bond acceptors, except for **5c** and **7c,d**. The molecular weights of compounds **5c**,**e**, **7c**,**e**, and **9a–c**,**e** are each greater than 500 Da. The Log *P-*value is not more than five for most compounds, except **5a**,**e**,**f** and **9c**,**d**. These results indicated that all compounds seem to be oral drug candidates, except compounds **5c**,**e**, **7c**, and **9c**,**e** which all violated RO5; however, some drugs in clinical use do not obey RO5. The TPSA was less than 140 Å^2^ in all compounds, except **3a**, **7a–f**, and **9a**,**b**, which indicated easy permeability through the intestinal cell membrane. Based on these findings and the results of the biological tests, all tested compounds met the in silico criteria for orally active drugs and showed promising biological activity; thus, these compounds will be studied further in vivo for development as orally active agents.

## 3. Materials and Methods 

### 3.1. Chemistry

The starting material 4-hydroxy-6-methylcoumarin (**1b**) was synthesised according to the method reported by Shah and colleagues [42]. All other reagents and solvents were obtained from commercial suppliers and used without further purification. Melting points (MP; °C, uncorrected) were recorded using a melting point apparatus (Electrothermal, Staffordshire, UK). The infrared (IR) spectra (expressed in wave number υ [cm^−1^]) were recorded by the SHIMADZU FT/IR S1 Plus Spectrometer (Shimadzu Europa GmbH, Duisburg, Germany) using potassium bromide (KBr) discs. The Agilent Technologies 600 Ultra Shield NMR Spectrometer (Santa Clara, CA, USA) was utilised to obtain NMR spectra at 600 and 154 MHz for ^1^H and ^13^C, respectively. Chemical shifts are expressed in δ (ppm); coupling constants are reported in Hz. CDCl_3_ and DMSO-*d*_6_ were used as solvents. The splitting patterns were designated as: s (singlet), d (doublet), dd (doublet of doublets), t (triplet), q (quartet), m (multiplet), br (broad), and br s (broad singlet). The Bruker Ascend 700 NMR Spectrometer (Bruker, Fällanden, Switzerland) was utilised to obtain NMR spectra of compounds **2a**, **4a**, **5d**, and **9c**,**d,f** at 700.17 and 176.08 MHz for ^1^H and ^13^C, respectively. The ^13^C-NMR spectrum of compound **5c** was obtained at 176.08 MHz. The ^1^H and ^13^C-NMR spectra for three compounds in each scheme were presented in the Supplementary Materials including one intermediate and two target compounds (**2a**, **4a**, **5c**; **6b**, **8e**, **9f**; Figures S1–S16). These data include two dimensional NMR spectra of **4a** and **8e**. The Shimadzu GC-MS-QP 2010 instrument (Shimadzu Europa GmbH, Duisburg, Germany) was used to record the electron impact mass spectra (EI-MS) at 70 eV. Elemental analyses (C, H, and N) were carried out at the Microanalytical Center of the Faculty of Science of Cairo University, Cairo, Egypt. They aligned with the proposed structures within ± 0.1–0.3% of the theoretical values. The microwave reaction was conducted using a microwave reactor (Biotage Initiator+, EXP EU, 400 W, 2450 MHz; Biotage, Uppsala, Sweden). The reactions were monitored by thin-layer chromatography (TLC) using silica gel-precoated aluminium sheets (60 F254, Merck, Kenilworth, NJ, USA) and visualised with UV at 365 and 254 nm.

#### 3.1.1. 4-Hydroxy-6-methylcoumarin (**1b**) 

This compound was synthesised following the reported procedure [42]. Yield: 75.67%. MP: 259–261 °C. EI-MS, *m/z* (Rel. Int.%): 176 (M^+^, 40.7), 147.

#### 3.1.2. 2-Amino-9-methyl-5-oxo-4-aryl-4*H*,5*H*-pyrano[3,2-*c*]chromene-3-carbonitriles (**2a–b**)

These compounds were synthesised according to the reported procedure [22]. A mixture of arylaldehyde namely, *p*-nitrobenzaldehyde or furfuraldehyde, (1mmol), 4-hydroxy-6-methylcoumarin (0.176 g 1mmol), malononitrile (0.066 g, 1mmol) and KHP (0.2 g) in distilled water (5 mL) was heated at 50 ^o^C for 5–6 h. After the completion of the reaction, the reaction mixture was cooled to room temperature and the solid was filtered off, washed with cold distilled water, dried, and recrystallised with CHCL_3_/ethanol to give compounds **2a,b**.

(±)-2-Amino-9-methyl-4-(4-nitrophenyl)-5-oxo-4*H*,5*H*-pyrano[3,2-*c*]chromene-3-carbonitrile (**2a**): Yield: 98%; yellow solid. MP: 259–261 °C. IR (υ_max_, cm^−1^): 3460, 3346 (NH_2_), 2204 (CN), 1716 (C=O). ^1^H-NMR (DMSO-*d*_6_, 600 MHz): δ 2.44 (s, 3H, Ar-CH_3_), 4.6 (s, 1H, CH-4), 7.3 (d, *J* = 8.4, 1H, Ar-H), 7.55–7.59 (m, 5H, Ar-H and NH_2_), 7.73 (s, 1H, Ar-H-10), 8.2 (d, *J* = 9, 2H, Ar-H-3′, 5′). ^13^C-NMR (DMSO-*d*_6_, 154 MHz): δ 20.9 (CH_3_), 37.2 (C-4), 57.1 (C-3), 103.1 (C-4’a), 113.05, 116.9, 119.3, 122.6, 124.2, 129.6, 134.4, 134.6, 147.0, 150.9, 151.3, 154.3, 158.5, 160.1 (Ar-C, CN and C=O). EI-MS, *m/z* (%): 377 (M + 2, 0.13), 73, consistent with the molecular formula C_20_H_13_N_3_O_5_ (375.33).

(±)-2-Amino-4-(furan-2-yl)-9-methyl-5-oxo-4*H*,5*H*-pyrano[3,2-*c*]chromene-3-carbonitrile (**2b**): Yield: 93.7%; grey solid. MP: 251–253 °C. IR (υ_max_, cm^−1^): 3392, 3323 (NH_2_), 2198 (CN), 1701 (C=O). ^1^H-NMR (DMSO-*d*_6_, 600 MHz): δ 2.38 (s, 3H, Ar-CH_3_), 4.57 (s, 1H, CH-4), 6.2 (d, *J* = 2.4, 1H, Ar-H), 6.3 (d, *J* = 3.2, 1H, Ar-H), 7.3 (d, *J* = 8.4, 1H, Ar-H), 7.44 (s, 2H, NH_2_), 7.49 (m, 2H, Ar-H), 7.6 (s,1H, Ar-H, CH-10). ^13^C-NMR (DMSO-*d*_6_, 154 MHz): δ 20.95 (CH_3_), 37.3 (C-4), 55.6 (C-3), 102.3 (C-4’a), 106.8, 110.6, 113.0, 116.8, 119.1, 122.3, 133.8, 134.4, 142.8, 150.8, 152.9, 153.9, 158.7, 160.3 (Ar-C, CN and C=O). EI-MS, *m/z* (%): 321 (M + 1, 0.15), 256, consistent with the molecular formula C_18_H_12_N_2_O_4_ (320.3).

#### 3.1.3. 2-Amino-9-methyl-5-oxo-4-aryl-4*H*,5*H*-pyrano[3,2-c]chromene-3-carboxamides (**3a,b**)

A mixture of **2a** or **2b** (0.01 mol) and concentrated H_2_SO_4_ (15 mL) was kept at room temperature (25 ± 2 °C) with continuous stirring for 10–14 h and then poured on cold water. The solid was filtered, washed with distilled water, dried, and crystallised with the indicated solvent.

(±)-2-Amino-9-methyl-4-(4-nitrophenyl)-5-oxo-4*H*,5*H*-pyrano[3,2-*c*]chromene-3-carboxamide (**3a**): Crystallisation solvent: ethanol (EtOH)/CHCl_3_. Yield: 92.9%; yellow solid. MP: 142–144 °C. IR (υ_max_, cm^−1^): 3446, 3408 (NH_2_), 1734 (C=O ester), 1676 (C=O amide). ^1^H-NMR (DMSO-*d*_6_,, 600 MHz): δ 2.34 (s, 3H, CH_3_), 4.86 (s, 1H, CH-4), 7.0 (s, 2H, NH_2_), 7.1 (d, *J* = 9, 2H, Ar-H, CH-2′,6′), 7.3 (d, *J* = 8.4, 1H, Ar-H), 7.5 (s, 2H, NH_2_), 7.6 (d, *J* = 7.2, 1H, Ar-H), 7.7 (s, 1H, Ar-H, CH-10), 8.1 (d, *J* = 7.8, 2H, Ar-H, CH-3′,5′). ^13^C-NMR (DMSO-*d*_6_, 154 MHz): 20.9 (CH_3_), 40.4 (C-4) 54.1 (C-3), 103.4 (C-4’a), 113.0, 116.2, 123.32, 124.6, 129.0, 129.7, 133.2, 146.2, 150.1, 150.7, 151.5, 160.6, 162.4, 169.9 (Ar-C, CN and C=O). EI-MS, *m/z* (%): 393 (M^+^, 1.1), 204, consistent with the molecular formula C_20_H_15_N_3_O_6_ (393.36).

(±)-2-Amino-4-(furan-2-yl)-9-methyl-5-oxo-4*H*,5*H*-pyrano[3,2-*c*]chromene-3-carboxamide (**3b**): Crystallisation solvent: EtOH. Yield: 62.5%; brown solid. MP: 202–204 °C. IR (υ_max_, cm^−1^): 3446, 3425 (NH_2_), 1724 (C=O ester), 1685 (C=O amide). ^1^H-NMR (DMSO-*d*_6_,, 600 MHz): δ 2.22 (s, 3H, CH_3_), 4.83 (s, 1H, H-4), 6.49 (d, *J* = 2.4, 1H, Ar-H), 6.78 (d, *J* = 3.2, 1H, Ar-H), 7.04–7.51 (m, 7H, Ar-H and NH_2_), 7.79 (s, 1H, Ar-H-10). ^13^C-NMR (DMSO-*d*_6_, 154 MHz): 20.7 (CH3), 38.6 (C-4), 59.4 (C-3), 103. (C-4’a), 109.0, 115.6, 116.5, 121.5, 123.0, 123.4, 133.1, 134.1, 143.6, 151.7, 152.7, 162.7, 163.5, 166.5 (Ar-C, CN and C=O). EI-MS, *m/z* (%): 339 (M + 1, 0.1), 338 (M^+^, 0.1), 296 (25.82), 73, consistent with the molecular formula C_18_H_14_N_2_O_5_ (338.31).

#### 3.1.4. *N*′-(3-cyano-9-methyl-5-oxo-4-aryl-4*H*,5*H*-pyrano[3,2-c]chromen-2-yl)-*N*,*N*-dimethyl formimidamides (**4a,b**)

A mixture of **2a** or **2b** (2.7 mmol) and DMF-DMA (25 mL) was heated at 100 °C for 15–40 min. The solid was obtained by filtration, washed with EtOH, dried, and then crystallised with CHCl_3_/EtOH.

(±)-*N*′-(3-cyano-9-methyl-4-(4-nitrophenyl)-5-oxo-4*H*,5*H*-pyrano[3,2-*c*]chromen-2-yl)-*N*,*N*-dimethylformimidamide (**4a**): Yield: 66.3%; yellow solid. MP: 279–281 °C. IR (υ_max_, cm^−1^): 2204 (CN), 1722 (C=O), 1664 (C=N). ^1^H-NMR (DMSO-*d*_6_, 600 MHz): δ 2.4 (s, 3H, Ar-CH_3_), 3.06 (s, 3H, N-CH_3_), 3.2 (s, 3H, N-CH_3_), 4.7 (s,1H, CH-4), 7.3 (d, *J* = 8.4, 1H, Ar-H), 7.5 (dd, *J* = 1.4 and *J* = 8.4, 1H, Ar-H), 7.6 (dd, *J* = 2.1 and *J* = 7, 2H, Ar-H), 7.9 (d, *J* = 1.4, 1H, Ar-H), 8.2 (dd, *J* = 2.1 and *J* = 7, 2H, Ar-H), 8.5 (s,1H, N=CH). ^13^C-NMR (DMSO-*d*_6_, 154 MHz): 20.8 (Ar-CH_3_), 34.9 (N-CH_3_), 38.7 (C-4), 41.2 (N-CH_3_), 73.0 (C-3), 102.6 (C-4’a), 113.3, 116.7, 119.2, 123.3, 124.2, 129.8, 134.5, 134.8, 147.1, 150.7, 150.9, 154.9, 155.3, 157.9, 160.3 (Ar-C, CN and C=O). EI-MS, *m/z* (%): 431 (M + 1, 0.1), 430 (M^+^, 0.14), 415 (5.77), 73, consistent with the molecular formula C_23_H_18_N_4_O_5_ (430.41).

(±)-*N*′-(3-cyano-4-(furan-2-yl)-9-methyl-5-oxo-4*H*,5*H*-pyrano[3,2-*c*]chromen-2-yl)-*N*,*N*-dimethylformimidamide (**4b**): Yield: 39%; white solid. MP: 273–275 °C. IR (υ_max_, cm^−1^): 2198 (CN), 1724 (C=O), 1654 (C=N). ^1^H-NMR (CDCl_3_, 600 MHz): δ 2.4 (s, 3H, Ar-CH_3_), 3.1 (s, 3H, N-CH_3_), 3.2 (s, 3H, N-CH_3_), 4.8 (s, 1H, CH-4), 6.31–6.36 (m, 2H, Ar-H), 7.23–7.26 (m, 2H, Ar-H), 7.37 (d, *J* = 8.4, 1H, Ar-H), 7.5 (s, 1H, Ar-H, CH-10), 8.2 (s, 1H, N=CH). ^13^C-NMR (CDCl_3_, 154 MHz): 21.0 (Ar-CH_3_), 32.6 (C-4), 35.0 (N-CH_3_), 41.2 (N-CH_3_), 72.9 (C-3), 101.4 (C-4’a), 107.6, 110.8, 113.4, 116.8, 118.7, 121.8, 133.6, 134.1, 142.2, 150.9, 152.7, 153.5, 154.8, 159.1, 160.5 (Ar-C, CN and C=O). EI-MS, *m/z* (%): 376 (M + 1, 1.9), 99, consistent with the molecular formula C_21_H_17_N_3_O_4_ (375.38).

#### 3.1.5. 2-(Arylideneamino)-9-methyl-4-(4-nitrophenyl)-5-oxo-4*H*,5*H*-pyrano[3,2-c]chromene-3-carbonitriles (**5a–f**)

An aromatic aldehyde (benzaldehyde, 2,4-dichlorobenzaldehyde, or 2,3,4-trimethoxybenzaldehyde, 1 mmol) was added to a mixture of **2a** or **2b** (1mmol) in 1,4-dioxane and then irradiated in a Biotage Initiator+ microwave for 90–110 min (TLC monitoring). After solvent evaporation, the residue was washed with toluene to obtain pure product and dried.

(±)-2-(benzylideneamino)-9-methyl-4-(4-nitrophenyl)-5-oxo-4*H*,5*H*-pyrano[3,2-*c*]chromene-3-carbonitrile (**5a**): Yield: 23.5%; buff solid. MP: 208–209 °C. IR (υ_max_, cm^−1^): 2198 (CN), 1701 (C=O), 1670 (C=N). ^1^H-NMR (DMSO-*d*_6_, 600 MHz): 2.39 (s, 3H, CH_3_), 4.65 (s, 1H, CH-4), 7.2–7.7 (m, 9H, Ar-H), 8.1–8.3 (m, 4H, Ar-H and N=CH). ^13^C-NMR (DMSO-*d*_6,_ 154 MHz): 20.9 (CH_3_), 37.2 (C-4), 57.1 (C-3), 103.1 (C-4’a), 112.9, 116.8, 119.3, 122.6, 124.2, 128.0, 128.6, 129.0, 129.3, 129.5, 134.2, 134.4, 134.6, 147.0, 150.9, 151.30, 158.4, 160.1 (Ar-C, CN and C=O). EI-MS, *m/z* (%): 465 (M + 2, 3.7), 57, consistent with the molecular formula C_27_H_17_N_3_O_5_ (463.44).

(±)-2-(benzylideneamino)-4-(furan-2-yl)-9-methyl-5-oxo-4*H*,5*H*-pyrano[3,2-*c*]chromene-3-carbonitrile (**5b**): Yield: 5.8%; brown solid. MP: 250–252 °C. IR (υ_max_, cm^−1^): 2194 (CN), 1703 (C=O), 1670 (C=N). ^1^H-NMR (DMSO-*d*_6_, 600 MHz): 2.4 (s, 3H, CH_3_), 4.4 (s, 1H, CH-4), 7.2–7.5 (m, 11H, Ar-H), 7.7 (s, 1H, N=CH). ^13^C-NMR (DMSO-*d*_6,_ 154 MHz): 20.9 (CH_3_), 37.3 (C-4), 58.4 (C-3), 104.3 (C-4’a), 107.7, 110.5, 113.0, 116.8, 119.7, 122.5, 127.6, 128.0, 128.6, 129.0, 129.3, 134.2, 134.5, 143.8, 150.7, 153.8, 158.4, 160.1 (Ar-C, CN and C=O). EI-MS, *m/z* (%): 410 (M + 2, 4), 409 (M + 1, 3), 408 (M^+^, 18), 56, consistent with the molecular formula C_25_H_16_N_2_O_4_ (408.41).

(±)-9-methyl-4-(4-nitrophenyl)-5-oxo-2-((3,4,5-trimethoxybenzylidene)amino)-4*H*,5*H*-pyrano[3,2-*c*]chromene-3-carbonitrile (**5c**): Yield: 30.5%; buff solid. MP: 221–223 °C. IR (υ_max_, cm^−1^): 2200 (CN), 1702 (C=O), 1672 (C=N). ^1^H-NMR (DMSO-*d*_6_, 600 MHz): 2.3 (s, 3H, CH_3_), 3.6 (s, 3H, OCH_3_), 3.9 (s, 6H, 2OCH_3_), 4.6 (s, 1H, CH-4), 6.4 (s, 1H, Ar-H), 6.5 (s, 1H, Ar-H), 7.2–7.7 (m, 5H, Ar-H), 8.0–8.1 (m, 3H, Ar-H and N=CH). ^13^C-NMR (DMSO-*d*_6_, 154 MHz): 20.8 (CH_3_), 37.2 (C-4), 56.2 (2OCH_3_, 3′, 5′-trimethoxyphenyl), 57.2 (C-3), 60.7 (OCH_3_), 102.9 (C-4’a), 107.1, 112.8, 116.8, 119.4, 122.7, 123.8, 124.2, 129.5, 131.1, 134.6, 136.8, 139.5, 147.0, 151.2, 153.2, 158.5, 160.4, 160.5 (Ar-C, CN and C=O). EI-MS, *m/z* (%): 555 (M + 2, 0.1), 554 (M + 1, 0.23), 204, consistent with the molecular formula C_30_H_23_N_3_O_8_ (553.52).

(±)-4-(furan-2-yl)-9-methyl-5-oxo-2-((3,4,5-trimethoxybenzylidene)amino)-4*H*,5*H*-pyrano[3,2-*c*]chromene-3-carbonitrile (**5d**): Yield: 17.5%; brown solid. MP: 248–250 °C. IR (υ_max_, cm^−1^): 2196 (CN), 1701 (C=O), 1664 (C=N). ^1^H-NMR (DMSO-*d*_6_, 600 MHz): δ 2.4 (s, 3H, CH_3_), 3.3 (s, 3H, OCH_3_), 3.6–3.7 (m, 6H, 2OCH_3_), 4.4 (s, 1H, CH-4), 6.42–6.49 (m, 4H, Ar-H), 7.2–7.7 (m, 4H, Ar-H), 9.4 (s, 1H, N=CH). ^13^C-NMR (DMSO-*d*_6_, 154 MHz): 20.9 (CH_3_), 37.6 (C-4), 56.3 (2OCH_3_, 3′, 5′-trimethoxyphenyl), 58.3 (C-3), 60.3 (OCH_3_), 104.0 (C-4’a), 105.2, 113.1, 116.8, 119.7, 122.6, 134.1, 134.4, 137.0, 139.5, 150.7, 153.2, 153.9, 158.4, 160.2, 160.3 (Ar-C, CN and C=O). EI-MS, *m/z* (%): 496 (M − 2, 1.18), 135, consistent with the molecular formula C_28_H_22_N_2_O_7_ (498.48).

(±)-2-((2,4-dichlorobenzylidene)amino)-9-methyl-4-(4-nitrophenyl)-5-oxo-4*H*,5*H*-pyrano[3,2-*c*]chromene-3-carbonitrile (**5e**): Yield: 37.2%; buff solid. MP: 236–238 °C. IR (υ_max_, cm^−1^): 2198 (CN), 1718 (C=O), 1676 (C=N). ^1^H-NMR (DMSO-*d*_6_, 600 MHz): δ 2.4 (s, 3H, CH_3_), 4.6 (s, 1H, CH-4), 7.03–8.4 (m, 10H, Ar-H), 10.2 (s, 1H, N=CH). ^13^C-NMR (DMSO-*d*_6_, 154 MHz): 20.96 (CH_3_), 37.2 (C-4), 57.1 (C-3), 103.1 (C-4’a), 113.0, 116.9, 119.3, 122.6, 123.7, 124.2, 128.6, 129.1, 129.6, 129.7, 130.7, 131.1, 131.9, 134.4, 134.6, 147.0, 150.9, 151.3, 158.5, 160.1(Ar-C, CN and C=O). EI-MS, *m/z* (%): 533 (M + 1, 0.2), 532 (M^+^, 3.7), 531 (M − 1, 8.7), 530 (M − 2, 27.8), 291, consistent with the molecular formula C_27_H_15_Cl_2_N_3_O_5_ (532.33).

(±)-2-((2,4-dichlorobenzylidene)amino)-4-(furan-2-yl)-9-methyl-5-oxo-4*H*,5*H*-pyrano[3,2-*c*]chromene-3-carbonitrile (**5f**): Yield: 17.8%; brown solid. MP: >300 °C. IR (υ_max_, cm^−1^): 2220 (CN), 1718 (C=O), 1647 (C=N). ^1^H-NMR (DMSO-*d*_6_, 600 MHz): δ 2.38 (s, 3H, CH_3_), 4.4 (s, 1H, CH-4), 7.2–8.1 (m, 9H, Ar-H), 10.1 (s, 1H, N=CH). ^13^C-NMR (DMSO-*d*_6_, 154 MHz): 20.8 (CH_3_), 37.4 (C-4), 58.5 (C-3), 100.1 (C-4’a), 107.2, 113.5, 116.68, 117.1, 119.7, 122.2, 122.8, 125.6, 126.2, 128.6, 129.3, 130.9, 131.7, 133.7, 134.3, 148.2, 149.5, 152.2, 158.9, 160.3 (Ar-C, CN and C=O). EI-MS, *m/z* (%): 478 (M + 1, 1.12), 476 (M − 1, 2.72), 475 (M − 2, 6), 474 (M − 3, 8), 91, 73, consistent with the molecular formula C_25_H_14_Cl_2_N_2_O_4_ (477.3).

#### 3.1.6. 6-substituted-4-hydroxyl-2-oxo-2*H*-chromene-3-sulfonyl chloride (**6a**,**b**)

Chlorosulfonic acid (13.5 mmol) was added dropwise to a solution of **1a** or **1b** (11.3 mmol) in dichloromethane (22.7 mL) cooled to 0 °C. The reaction mixture was continuously stirred at ambient temperature overnight. The precipitates were filtered, washed with dichloromethane and petroleum ether, and then dried.

6-Chloro-4-hydroxy-2-oxo-2*H*-chromene-3-sulfonyl chloride (**6a**): Yield: 97%; white solid. MP: 118–120 °C. IR (υ_max_, cm^−1^):3317 (br., OH), 1685 (C=O), 1375 (asymmetric, S-O), 1176 (symmetric, S-O). ^1^H-NMR (DMSO-*d*_6_, 600 MHz): δ 7.3–7.6 (m, 2H, Ar-H), 7.7 (s, 1H, Ar-H-5), 14.0 (br. s, 1H, OH). ^13^C-NMR (DMSO-*d*_6_, 154 MHz): 108.54 (C-3), 116.7, 118.9, 123.8, 128.6, 133.7, 151.6, 156.9, 161.6 (Ar-C, CN and C=O). EI-MS, *m/z* (%): 299 (M + 4, 0.2), 298 (M + 3, 0.4), 297 (M + 2, 2.1), 296 (M + 1, 3.5), 295 (M^+^, 13.21), 294 (M − 1, 0.5), 293 (M − 2, 0.1), 207, consistent with the molecular formula C_9_H_4_Cl_2_O_5_S (295.1).

4-Hydroxy-6-methyl-2-oxo-2*H*-chromene-3-sulfonyl chloride (**6b**): Yield: 98%, white solid. MP: 116–117 °C. IR (υ_max_, cm^−1^): 3381 (br., OH), 1695 (C=O), 1328 (asymmetric, S-O), 1131 (symmetric, S-O). ^1^H-NMR (DMSO-*d*_6_, 600 MHz): δ 2.34 (s, 3H, CH_3_), 7.2 (d, *J* = 7.8, 1H, Ar-H-7), 7.4 (d, *J* = 8.4, 1H, Ar-H-8), 7.6 (s, 1H, Ar-H-5), 13.9 (br. s, 1H, OH). ^13^C-NMR (DMSO-*d*_6_, 154 MHZ): 20.7 (CH_3_), 107.9 (C-3), 114.9, 116.4, 124.2, 133.9, 134.8, 151.1, 157.5, 162.7(Ar-C, CN and C=O). EI-MS, *m/z* (%): 278 (M + 4, 0.07), 277 (M + 3, 0.52), 276 (M + 2, 2.72), 275 (M + 1, 0.23), 274 (M^+^, 0.05), 44, consistent with the molecular formula C_10_H_7_ClO_5_S (274.68).

#### 3.1.7. 4-Hydroxy-6-(substituted) coumarin-3-sulfonamides (**7a–f**)

A mixture of **6a** or **6b** (0.002 mol), sulfa compounds (sulfanilamide, sulfadiazine, or sulfathiazole, 0.002 mol), and absolute EtoH (15 mL) was heated under reflux for 3–6 h (TLC monitoring). After cooling, the solid was filtered out, washed with absolute EtOH and petroleum ether, and then dried to obtain the pure products.

6-Chloro-4-hydroxy-2-oxo-*N*-(4-sulfamoylphenyl)-2*H*-chromene-3-sulfonamide (**7a**): Yield: 27%, white solid. MP: 245–247 °C. IR (υ_max_, cm^−1^):3346–3257 (NH_2_, NH), 3101 (br, OH), 1726 (C=O), 1338 (asymmetric S-O), 1165 (symmetric S-O). ^1^H-NMR (DMSO-*d*_6_, 600 MHz): δ 5.62 (br. s, 2H, NH_2_), 6.9–7.0 (m, 3H, Ar-H-2’, 6’ and NH), 7.4 (s, 1H, Ar-H-5), 7.6–7.8 (m, 4H, Ar-H). ^13^C-NMR (DMSO-*d*_6_, 154 MHz): 108.5 (C-3), 116.8, 118.9, 119.0, 122.8, 123.8, 127.8, 128.6, 132.8, 133.7, 151.7, 157.0, 161.6 (Ar-C, CN and C=O) EI-MS, *m/z* (%): 433 (M + 3, 0.1), 432(M + 2, 0.22), 431 (M +1, 0.85), 430 (M^+^, 1.9), 73, consistent with the molecular formula C_15_H_11_ClN_2_O_7_S_2_ (430.84).

4-Hydroxy-6-methyl-2-oxo-*N*-(4-sulfamoylphenyl)-2*H*-chromene-3-sulfonamide (**7b**): Yield: 56%; white solid. MP: 241–243 °C. IR (υ_max_, cm^−1^): 3352, 3250 (NH_2_, NH), 3142 (br., OH), 1680 (C=O), 1355 (asymmetric S-O), 1157 (symmetric S-O). ^1^H-NMR (DMSO-*d*_6_, 600 MHz): δ 2.36 (s, 3H, CH_3_), 2.49 (s, 1H, NH), 4.55 (br. s, 2H, NH_2_), 6.9 (d, *J* = 8.4, 2H, Ar-H-2’, 6’), 7.2 (d, *J* = 8.4, 1H, Ar-H-7), 7.4 (d, *J* = 7.8, 1H, Ar-H-8), 7.6 (m, 3H, Ar-H- 3’, 5’ and 5), 13.9 (br. s, 1H, OH). ^13^C-NMR (DMSO-*d*_6_, 154 MHz): 20.78 (CH_3_), 107.9 (C-3), 114.9, 116.5, 117.8, 124.2, 127.8, 133.9, 134.9, 151.1, 157.6, 162.7 (Ar-C, CN and C=O). EI-MS, *m/z* (%): 411 (M + 1, 0.51), 410 (M^+^, 2.62), 408 (M − 2, 12.76), 133, consistent with the molecular formula C_16_H_14_N_2_O_7_S_2_ (410.42).

6-Chloro-4-hydroxy-2-oxo-*N*-(4-(*N*-(pyrimidin-2-yl)sulfamoyl)phenyl)-2*H*-chromene-3-sulfonamide (**7c**): Yield: 78.2%; yellow solid. MP: 230–232 °C. IR (υ_max_, cm^−1^): 3373 (2NH), 3084 (br., OH), 1691 (C=O), 1346 (asymmetric S-O), 1151 (symmetric S-O). ^1^H-NMR (DMSO-*d*_6_, 600 MHz): δ 4.7 (br. s, 1H, NH), 6.6 (d, *J* = 9.6, 2H, Ar-H-2’, 6’), 7.0 (m, 1H, diazine-H-4), 7.4–7.7 (m, 5H, Ar-H and NH), 7.8 (s, 1H, Ar-H-5), 8.4 (m, 2H, diazine-H-3, 5). ^13^C-NMR (DMSO-*d*_6_, 154 MHz): 108.6 (C-3), 113.7, 116.0, 116.8, 118.9, 123.8, 126.8, 128.6, 130.2, 133.6, 151.7, 156.9, 157.6, 158.7, 161.6 (Ar-C, CN and C=O). EI-MS, *m/z* (%): 511 (M + 3, 0.6), 315, consistent with the molecular formula C_19_H_13_ClN_4_O_7_S_2_ (508.9).

4-Hydroxy-6-methyl-2-oxo-*N*-(4-(*N*-(pyrimidin-2-yl)sulfamoyl)phenyl)-2*H*-chromene-3-sulfonamide (**7d**): Yield: 86.3%.; yellow solid. MP: 207–209 °C. IR (υ_max_, cm^−1^): 3620.39 (OH), 3423, 3356 (2NH), 1691 (C=O), 1334 (asymmetric S-O), 1166 (symmetric S-O). ^1^H-NMR (DMSO-*d*_6_, 600 MHz): δ 2.39 (s, 3H, CH_3_), 6.6 (d, *J* = 9.6, 2H, Ar-H-2’, 6’), 7.0 (m, 1H, diazine-H-4), 7.2 (d, *J* = 10.8, 1H, Ar-H-8), 7.4 (d, *J* = 9.6, 1H, Ar-H-7), 7.64–7.67 (m, 3H, Ar-H-5, 3’, 5’), 8.4 (m, 2H, diazine-H-3,5). ^13^C-NMR (DMSO-*d*_6_, 154 MHz): 20.78 (CH_3_), 108.0 (C-3), 113.3, 114.9, 116.0, 116.5, 124.2, 126.2, 128.2, 128.4, 130.2, 133.9, 134.8, 151.1, 157.6, 158.7, 162.7 (Ar-C, CN and C=O) EI-MS, *m/z* (%): 490 (M + 2, 2.54), 489 (M + 1, 5.08), 355, consistent with the molecular formula C_20_H_16_N_4_O_7_S_2_ (488.4).

6-Chloro-4-hydroxy-2-oxo-*N*-(4-(*N*-(thiazol-2-yl)sulfamoyl)phenyl)-2*H*-chromene-3-sulfonamide (**7e**): Yield: 74.13%; white solid. MP: 215–217 °C. IR (υ_max_, cm^−1^): 3448 (2NH), 3140 (br., OH), 1676 (C=O), 1338 (asymmetric S-O), 1147 (symmetric S-O). ^1^H-NMR (DMSO*-d*_6_, 600 MHz): δ 5.3 (br. s, 1H, NH), 5.4 (br. s, 1H, NH), 6.7 (d, *J* = 3.6, 1H, thiazole-H), 6.8 (d, *J* = 8.4, 2H, Ar-H-2’, 6’), 7.2 (d, *J* = 3.6, 1H, thiazole-H), 7.42 (d, *J* = 8.4, 1H, Ar-H-8), 7.58 (d, *J* = 9.6, 2H, Ar-H-3’ and 5’), 7.72 (d, *J* = 7.8, 1H, Ar-H-7), 7.8 (s, 1H, Ar, CH-5). ^13^C-NMR (DMSO-*d*_6_, 154 MHz): 108.3 (C-3), 108.6, 116.6, 116.8, 118.9, 123.8, 124.8, 128.1, 128.6, 132.8, 133.7, 147.1, 151.7, 157.0, 161.6, 168.7, (Ar-C, CN and C=O). EI-MS, *m/z* (%): 514 (M + 1, 0.2), 513 (M^+^, 1.2), 73, consistent with the molecular formula C_18_H_12_ClN_3_O_7_S_3_ (513.95).

4-Hydroxy-6-methyl-2-oxo-*N*-(4-(*N*-(thiazol-2-yl)sulfamoyl)phenyl)-2*H*-chromene-3-sulfonamide (**7f**): Yield: 84.7%; white solid. MP: 167–169 °C. IR (υ_max_, cm^−1^): 3421(OH) overlapped with 3338 (2NH), 1683 (C=O), 1350 (asymmetric S-O), 1153 (symmetric S-O). ^1^H-NMR (DMSO-*d*_6_, 600 MHz): δ 2.37 (s, 3H, CH_3_), 6.7 (m, 3H, Ar, CH-2’, 6’ and thiazole-H), 7.1 (d, *J* = 3.6, 1H, thiazole-H), 7.2 (d, *J* = 8.4, 1H, Ar-H-8), 7.4 (d, *J* = 7.8, 1H, Ar-H-7), 7.5 (d, *J* = 8.4, 2H, Ar-H-3’, 5’), 7.6 (s, 1H, Ar-H-5), 13.9 (br. s, 1H, OH). ^13^C-NMR (DMSO-*d*_6_, 154 MHz): 20.78 (CH_3_), 107.9 (C-3), 108.1, 114.9, 115.3, 116.5, 124.2, 124.8, 128.1, 131.2, 133.9, 134.9, 145.9, 151.1, 157.5, 168.6, 162.7 (Ar-C, CN and C=O). EI-MS, *m/z* (%): 493 (M^+^, 0.1), 491(M − 2, 3.2), 490 (M − 3, 3.2), 73, consistent with the molecular formula C_19_H_15_N_3_O_7_S_3_ (493.53).

#### 3.1.8. 4-Hydroxy-6-(substituted)coumarin-3-sulfonamides (**8a–f**)

A mixture of **6a** or **6b** (0.002 mol), 4-substituted-aniline (aniline, 4-hydroxyaniline, or 4-acetylaniline, 0.004 mol) and absolute EtOH (15 mL) was refluxed for 6–8 h. While warm, the solid was collected by filtration and then washed with hot absolute EtOH and petroleum ether, respectively. After drying, the solid was recrystallised if necessary.

6-Chloro-4-hydroxy-2-oxo-*N*-phenyl-2*H*-chromene-3-sulfonamide (**8a**): Yield: 92.65%. MP: 337–339 °C. IR (υ_max_, cm^−1^): 3367 (NH), 3169 (br., OH), 1683 (C=O), 1348 (asymmetric S-O), 1168 (symmetric S-O). ^1^H-NMR (DMSO-*d*_6_, 600 MHz): δ 7.2–7.4 (m, 6H, Ar-H), 7.72 (d, *J* = 8.4*,* 1H, Ar-H), 7.8 (s, 1H, Ar-H-5). ^13^C-NMR (DMSO-*d*_6_, 154 MHz): 108.6 (C-3), 116.8, 117.6, 118.9, 122.7, 123.8, 127.5, 128.6, 130.2, 133.7, 151.7, 157.0, 161.6 (Ar-C, CN and C=O). EI-MS, *m/z* (%): 350 (M − 1, 1.18), 57, consistent with the molecular formula C_15_H_10_ClNO_5_S (351.76).

4-Hydroxy-6-methyl-2-oxo-*N*-phenyl-2*H*-chromene-3-sulfonamide (**8b**): Yield: 84.5%; white solid. MP: 324–325 °C. IR (υ_max_/cm^−1^): 3356 (NH), 3111 (br., OH), 1685 (C=O), 1354 (asymmetric S-O), 1159 (symmetric S-O). ^1^H-NMR (DMSO-*d*_6_, 600 MHz): δ 2.37 (s, 3H, CH_3_), 7.3–7.6 (m, 8H, Ar-H), 9.6 (br. s, 1H, NH), 13.9 (br. s, 1H, OH). ^13^C-NMR (DMSO-*d*_6_, 154 MHz): 20.79 (CH_3_), 107.9 (C-3), 114.9, 116.5, 123.0, 124.2, 127.9, 130.2, 133.1, 133.9, 134.9, 151.1, 157.6, 162.7 (Ar-C, CN and C=O). EI-MS, *m/z* (%): 434 (M + 3, 1.65), 333 (M^+2^, 3.92), 332 (M^+1^, 26.25), 207, consistent with the molecular formula C_16_H_13_NO_5_S (331.34).

6-Chloro-4-hydroxy-*N*-(4-hydroxyphenyl)-2-oxo-2*H*-chromene-3-sulfonamide (**8c**): Crystallisation solvent: CHCl_3_. Yield: 48.3%; white solid. MP: 250–251 °C. IR (υ_max_, cm^−1^): 3278 (br., OH overlapped with NH), 1681 (C=O), 1346 (asymmetric S-O), 1168 (symmetric S-O). ^1^H-NMR (DMSO-*d*_6_, 600 MHz): δ 6.8 (m, 2H, Ar-H-2’, 6’), 7.1 (m, 2H, Ar-H-3’, 5’), 7.4 (d, *J* = 8.4, 1H, Ar,CH-8), 7.7 (d, *J* = 7.8, 1H, Ar,CH-7), 7.8 (s, 1H, Ar,CH-5), 9.7 (s, 1H, NH), 10.0 (br. s, 1H, OH). ^13^C-NMR (DMSO-*d*_6_, 154 MHz): 108.5 (C-3), 116.5, 116.8, 118.9, 123.8, 124.2, 128.6, 133.7, 151.7, 157.0, 160.1, 161.7 (Ar-C, CN and C=O). EI-MS, *m/z* (%): 366 (M − 1, 0.1), 363 (M − 4, 28.19), 332 (5.86), 196, consistent with the molecular formula C_15_H_10_ClNO_6_S (367.76).

4-Hydroxy-*N*-(4-hydroxyphenyl)-6-methyl-2-oxo-2*H*-chromene-3-sulfonamide (**8d**): Crystallisation solvent: CHCl_3_. Yield: 63.13%; white solid. MP: 253–254 °C. IR (υ_max_, cm^−1^): 3298 (br., OH overlapped with NH), 1662 (C=O), 1354 (asymmetric S-O), 1166 (symmetric S-O). ^1^H-NMR (DMSO-*d*_6_, 600 MHz): δ 2.38 (s, 3H, CH_3_), 6.8 (m, 2H, Ar, CH-2’, 6’), 7.1–7.2 (m, 3H, Ar, CH-3’, 5’ and 8), 7.4–7.6 (m, 2H, Ar, CH-7 and 5), 9.7 (br. s, 1H, NH). ^13^C-NMR (DMSO-*d*_6_, 154 MHz): 20.79 (CH_3_), 107.9 (C-3), 114.9, 116.5, 123.4, 124.2, 124.3, 133.9, 134.9, 151.1, 157.2, 157.6, 162.7, 173.7 (Ar-C, CN and C=O). EI-MS, *m/z* (%): 345 (M − 2, 0.04), 344 (M − 3, 0.48), 343 (M − 4, 3.31), 342 (M − 5, 9.7), 341 (M − 6, 52.35), 209, consistent with the molecular formula C_16_H_13_NO_6_S (347.34).

*N*-(4-acetylphenyl)-6-chloro-4-hydroxy-2-oxo-2*H*-chromene-3-sulfonamide (**8e**): Crystallisation solvent: CHCl_3_/isopropanol. Yield: 81.25%; white solid. MP: 238–240 °C. IR (υ_max_, cm^−1^): 3344 (NH), 3088 (br., OH), 1718 (C=O ketone), 1678 (C=O ester), 1357 (asymmetric S-O), 1166 (symmetric S-O). ^1^H-NMR (DMSO-*d*_6_, 600 MHz): δ 2.38 (s, 3H, CH_3_C=O), 6.6 (d, *J* = 8.4 2H, Ar, CH-2’, 6’), 7.3 (d, *J* = 7.8, 1H, Ar,CH-8), 7.6 (m, 3H, Ar,CH-3’, 5’ and 7), 7.7 (s, 1H, Ar,CH-5), 13.9 (br. s, 1H, OH). ^13^C-NMR (DMSO-*d*_6_, 154 MHz): 26.4 (CH_3_), 108.5 (C-3), 115.0, 116.7, 118.8, 123.8, 127.4, 128.5, 130.8, 133.6, 150.6, 151.6, 156.9, 161.6 (Ar-C, CN and C=O). 195.8 (C=O ketone). EI-MS, *m/z* (%): 397 (M + 4, 0.13), 391 (M − 2, 0.13), 390 (M − 3, 0.83), 54, consistent with the molecular formula C_17_H_12_ClNO_6_S (393.8).

*N*-(4-acetylphenyl)-4-hydroxy-6-methyl-2-oxo-2*H*-chromene-3-sulfonamide (**8f**): Crystallisation solvent: CHCl_3_/isopropanol. Yield: 74.5%; white solid. MP: 231–232 °C. IR (υ_max_, cm^−1^): 3342 (NH), 3068 (br., OH), 1728 (C=O ketone), 1707 (C=O ester), 1359 (asymmetric S-O), 1163 (symmetric S-O). ^1^H-NMR (DMSO-*d*_6_, 600 MHz): δ 2.37 (s, 3H, CH_3_), 2.38 (s, 3H, CH_3_C=O), 6.6 (d, *J* = 7.8, 2H, Ar,CH-2’, 6’), 7.2 (d, 1H, *J* = 9, Ar,CH-8), 7.4 (d, *J* = 8.4, 1H, Ar,CH-7), 7.66–7.69 (m, 3H, Ar,CH-5, 3’, 5’), 13.9 (br. s, 1H, OH). ^13^C-NMR (DMSO-*d*_6_, 154 MHz): 20.7 (CH_3_), 26.6 (CH_3_-C=O), 107.9 (C-3) 114.9, 116.4, 116.9, 124.2, 129.4, 130.7, 133.9, 134.9, 147.5, 151.1, 157.6, 162.77 (Ar-C, CN and C=O). 196.18 (C=O ketone). EI-MS, *m/z* (%): 377 (M + 4, 0.1), 476 (M + 3, 0.5), 375 (M + 2, 2.4), 374 (M + 1, 4), 373 (M^+^, 15.3), 73, consistent with the molecular formula C_18_H_15_NO_6_S (373.38).

#### 3.1.9. Coumarin-Sulfonamide Chalcones (**9a–f**)

Equimolar quantities of **8e** or **8f** (0.01 mol) and an aromatic aldehyde (4-nitrobenzaldehyde, 4-chlorobenzaldehyde, or 4-methoxybenzaldehyde, 0.01 mol) were dissolved in a minimal amount of EtOH. Sodium hydroxide solution (2 mL, 0.02 M) was added slowly and stirred at ambient temperature for 24 h. The mixture was poured slowly into 400 mL of ice water with constant stirring and then refrigerated for 24 h. The formed precipitates were filtered, washed with distilled water, dried, and recrystallised with CHCl_3_ if necessary.

*(E*)-6-Chloro-4-hydroxy-*N*-(4-(3-(4-nitrophenyl)acryloyl)phenyl)-2-oxo-2*H*-chromene-3-sulfonamide (**9a**): Yield: 35.34%; orange solid. MP: 205–207 °C. IR (υ_max_, cm^−1^): 3487 (OH), 3388 (NH), 1703 (C=O ester), 1637 (C=O ketone), 1342 (asymmetric S-O), 1180 (symmetric S-O). ^1^H-NMR (DMSO-*d*_6_, 600 MHz): δ 6.3–6.6 (m, 4H, Ar-H), 7.7 (d, *J* = 9, 1H, Ar-H), 7.9–8.3 (m, 9H, CH_α_=CH_β_, Ar-H and NH). ^13^C-NMR (DMSO-*d*_6_, 154 MHz): 101.1 (C-3), 113.2, 115.9, 122.0, 124.4, 124.6, 125.4, 127.2, 130.0, 130.4, 130.5, 130.8, 131.9, 139.1, 142.34, 148.2, 151.20, 157.7, 161.9 (Ar-C, CN and C=O), 185.92 (C=O ketone). EI-MS, *m/z* (%): 527 (M + 1, 8), 526 (M^+^, 11), 373, consistent with the molecular formula C_24_H_15_ClN_2_O_8_S (526.9).

(*E*)-4-Hydroxy-6-methyl-*N*-(4-(3-(4-nitrophenyl)acryloyl)phenyl)-2-oxo-2*H*-chromene-3-sulfonamide (**9b**): Yield: 30%; orange solid. MP: 220–220 °C. IR (υ_max_, cm^−1^): 3487 (OH), 3388 (NH), 1707 (C=O ester), 1637 (C=O ketone), 1340 (asymmetric S-O), 1182 (symmetric S-O). ^1^H-NMR (DMSO-*d*_6_, 600 MHz): δ 2.38 (s, 3H, CH_3_), 6.67–6.7 (m, 4H, Ar-H), 7.25 (s, 1H, Ar, CH-5), 7.4–8.3 (m, 9 H, CH_α_=CH_β_, Ar-H, and NH), 13.9 (br. s, 1H, OH). ^13^C-NMR (DMSO-*d*_6_, 154 MHz): 20.82 (CH_3_), 107.9 (C-3) 114.9, 116.4, 116.8, 121.67, 123.5, 124.3, 128.4, 129.3, 130.7, 133.9, 134.9, 138.2, 140.7, 141.94, 145.9, 151.1, 157.6, 162.32 (Ar-C, CN and C=O), 186.10 (C=O ketone). EI-MS, m/z (%): 506 (M^+^, 0.24), 503 (M − 3, 1.61), 73, consistent with the molecular formula C_25_H_18_N_2_O_8_S (506.48).

(*E*)-6-Chloro-*N*-(4-(3-(4-chlorophenyl)acryloyl)phenyl)-4-hydroxy-2-oxo-2*H*-chromene-3-sulfonamide (**9c**): Yield: 22.32%; yellow solid. MP: 157–159 °C. IR (υ_max_, cm^−1^): 3460 (OH), 3340 (NH), 1703 (C=O ester), 1629 (C=O ketone), 1346 (asymmetric S-O), 1176 (symmetric S-O). ^1^H-NMR (DMSO-*d*_6_, 600 MHz): 5.6 (s, 1H, NH), 6.6 (m, 2H, Ar-H), 7.4 (d, *J* = 8.4, 1H, Ar-H), 7.51 (d, *J* = 8.4, 1H, Ar-H), 7.61 (d, *J* = 16.1, 1H, C=CH_α_), 7.7 (dd.,*J* = 2.5, *J* = 6.3, 2H, Ar-H), 7.8 (d, *J* = 8.4, 1H, Ar-H), 7.89–7.92 (m, 3H, Ar-H and C=CH_β_), 7.95 (d, *J* = 8.4, 2H, Ar-H), 14.1 (br. s, 1H, OH). ^13^C-NMR (DMSO-*d*_6_, 154 MHz): 108.6, 113.4, 116.8, 118.9, 122.8, 123.6, 123.8, 128.6, 129.3, 130.6, 131.6, 132.8, 133.7, 134.6, 134.8, 140.4, 151.7, 156.9, 161.6 (Ar-C, CN and C=O). 186.1 (C=O ketone). EI-MS, *m/z* (%): 519 (M + 3, 0.3), 518 (M + 2, 0.6), 517 (M + 1, 1.6), 221, consistent with the molecular formula C_24_H_15_Cl_2_NO_6_S (516.35).

(*E*)-*N*-(4-(3-(4-chlorophenyl)acryloyl)phenyl)-4-hydroxy-6-methyl-2-oxo-2*H*-chromene-3-sulfonamide (**9d**): Yield: 22.41%; yellow solid. MP: 141–143 °C. IR (υ_max_, cm^−1^): 3462 (OH), 3342 (NH), 1701 (C=O ester), 1647 (C=O ketone), 1346 (asymmetric S-O), 1178 (symmetric S-O). ^1^H-NMR (DMSO-*d*_6_, 600 MHz): δ 2.39 (s, 3H, CH_3_), 6.7 (d, *J* = 8.4, 2H, Ar-H), 7.2 (d, *J* = 8.4, 1H, Ar-H), 7.4–7.5 (m, 3H, Ar-H), 7.6 (d, *J* = 15.4, 1H, C=CH_α_), 7.67 (s, 1H, Ar-H), 7.8–7.9 (m, 3H, Ar-H and C=CH_β_), 7.99 (d, *J* = 9.1, 2H, Ar-H), 14.0 (s, 1H, OH). ^13^C-NMR (DMSO-*d*_6_, 154 MHz): 20.80 (CH_3_), 108.03 (C-3), 114.5, 114.9, 116.5, 123.5, 124.3, 129.3, 129.8, 130.7, 131.5, 131.6, 133.9, 134.5, 134.8, 134.9, 140.7, 151.2, 157.5, 162.7 (Ar-C, CN and C=O). 186.44 (C=O ketone). EI-MS, m/z (%): 497 (M + 2, 1.1), 496 (M + 1, 10.4), 495 (M^+^, 60.46), 55, consistent with the molecular formula C_25_H_18_ClNO_6_S (495.93).

(*E*)-6-Chloro-4-hydroxy-*N*-(4-(3-(4-methoxyphenyl)acryloyl)phenyl)-2-oxo-2*H*-chromene-3-sulfonamide (**9e**): Yield: 30.23%; yellow solid. MP: >300 °C. IR (υ_max_, cm^−1^): 3468 (OH), 3329 (NH), 1695 (C=O ester), 1626 (C=O ketone), 1350 (asymmetric S-O), 1163 (symmetric S-O). ^1^H-NMR (DMSO-*d*_6_, 600 MHz): δ 3.8 (s, 3H, OCH_3_), 6.6 (d, *J* = 7.7, 2H, Ar-H), 6.9 (m, 2H, Ar-H), 7.09 (m, 2H, Ar-H), 7.35 (d, *J* = 8.4, 1H, Ar-H), 7.57 (d, *J* = 8.4, 1H, Ar-H), 7.7–7.9 (m, 3H, Ar-H, CH_α_=C and C=CH_β_), 8.2 (d, *J* = 8.4, 2H, Ar-H), 8.5 (s, 1H, NH). ^13^C-NMR (DMSO-*d*_6_, 154 MHz): 55.7 (OCH_3_), 108.3 (C-3), 113.1, 114.7, 119.9, 120.3, 121.6, 125.9, 128.2, 129.1, 130.4, 130.7, 131.2, 131.4, 135.2, 144.1, 141.8, 161.3, 157.1, 162.0 (Ar-C, CN and C=O). 186.3 (C=O ketone). EI-MS, m/z (%): 513 (M + 2, 0.12), 73, consistent with the molecular formula C_25_H_18_ClNO_7_S (511.93).

(*E*)-4-Hydroxy-*N*-(4-(3-(4-methoxyphenyl)acryloyl)phenyl)-6-methyl-2-oxo-2*H*-chromene-3-sulfonamide (**9f**): Yield: 15.2%; yellow solid. MP: >300 °C. IR (υ_max_, cm^−1^):3468 (OH), 3331 (NH), 1701 (C=O ester), 1626 (C=O ketone), 1342 (asymmetric S-O), 1163 (symmetric S-O). ^1^H-NMR (DMSO-*d*_6_, 600 MHz): 2.39 (s, 3H, CH_3_), 3.81 (s, 3H, OCH_3_), 6.8 (d, *J* = 7.7, 2H, Ar-H), 7.01 (d, *J* = 7.7, 2H, Ar-H), 7.2 (d, *J* = 8.4, 1H, Ar-H), 7.5 (d, *J* = 8.4, 1H, Ar-H), 7.6 (d, *J* = 15.4, 1H, C=CH_α_), 7.67 (s, 1H, Ar-H-5), 7.7 (d, *J* = 15.4, 1H, C=CH_β_), 7.8 (d, *J* = 7.7, 2H, Ar-H), 8.0 (d, *J* = 8.2, 2H, Ar-H), 14.04 (br. s, 1H, OH). ^13^C-NMR (DMSO-*d*_6_, 154 MHz): 20.79 (CH_3_), 55.8 (OCH_3_), 108.0 (C-3), 114.8, 114.9, 115.5, 116.5, 120.1, 124.3, 128.5, 130.8, 130.9, 131.2, 132.3, 133.9, 134.9, 142.5, 151.2, 157.6, 161.4, 162.7 (Ar-C, CN and C=O). 186.8 (C=O ketone). EI-MS, m/z (%): 491 (M^+^, 0.8), 490 (M − 1, 5.29), 78, consistent with the molecular formula C_26_H_21_NO_7_S (491.51).

#### 3.1.10. X-Ray Crystallographic Study of Compound **2a**

The Bruker-Nonius Apex X8 CCD Diffractometer was used to analyse the X-ray single-crystal structure. Yellow needles of compound **2a** were obtained by crystallisation from EtOH by allowing the solvent to slowly evaporate. Crystallographic data for the structure of compound **2a** have been deposited at the Cambridge Crystallographic Data Center (deposit CCDC 1985063).

### 3.2. Antioxidant Testing

The radical scavenging activity of the synthesised compounds was measured using the DPPH microplate-based method [25,26]. The absorbance was measured using a microplate/cuvette reader (Spectramax M5, Molecular Devices, San Jose, CA, USA). Reference standard ascorbic acid was utilised as the positive control for this assay. Test samples were prepared at a starting concentration of 200 μg/mL after mixing with 100 μL of 0.2 mM DPPH methanolic solution. A series of concentrations (1.563 to 200 μg/mL) were used to determine the 50% inhibitory concentration (IC_50_) of samples. Samples were incubated with DPPH in the dark for 30 min at room temperature (25 °C). The methanolic DPPH solution was prepared daily and stored in a flask covered with aluminium foil. DPPH radicals have an absorption maximum (λ_max_) at 515 nm, at which the absorbance values were measured and converted into a percentage of antioxidant activity [43].

The methanolic DPPH solution without antioxidants was used as a control, and samples in methanol alone served as a background control. IC_50_ was determined using GraphPad Prism 7 (GraphPad Software Inc., San Diego, CA, USA). The percent inhibition was calculated using the following Equation (1):

(1) $$\%\;Inhibition=100-\left[\frac{\left(\mathrm{Sample}\;\mathrm{Absorbance}\;-\mathrm{Sample}\;\mathrm{background}\;\mathrm{Absorbance}\right)}{\mathrm{DPPH}\;\mathrm{only}\;\mathrm{Absorbance}}\times 100\right]$$

The assay was carried out in triplicate and performed as much as possible in an area protected against light. The data points are presented as the mean ± standard deviation (SD, n = 3).

### 3.3. Antimicrobial Activity

#### 3.3.1. Agar Well Diffusion Method

Antimicrobial tests were conducted via the agar well diffusion method [27] using 100 μL of suspension containing 1 × 10^8^ CFU/mL pathological tested bacteria and 1 × 10^6^ CFU/mL yeast spread on NA and SDA, respectively. After the media cooled and solidified, wells (10 mm in diameter) were made in the solidified agar and loaded with 100 μL of the tested compound solution prepared by dissolving 10 mg of the chemical compound in 1 mL dimethyl sulfoxide (DMSO). The inoculated plates were then incubated for 24 h at 37 °C for bacteria and yeast growth. DMSO served as negative control. Ciprofloxacin (10 mg/mL) and ketoconazole (10 mg/mL) were used as standards for antibacterial and antifungal activity, respectively. After incubation, antimicrobial activity was evaluated by measuring the zone of inhibition against the test organisms and compared with that of the standard. Antimicrobial activities were expressed as the diameter of the inhibition zone (IZ) in mm. The experiment was carried out in triplicate and the average zone of inhibition was calculated.

#### 3.3.2. MIC Measurement

The bacteriostatic activity of the active compounds (having IZ > 16 mm) was then evaluated using the two-fold serial dilution technique [28]. Two-fold serial dilutions of the tested compounds solutions were prepared using the proper nutrient broth to reach final concentrations of 1000, 500, 250, and 125 μg/mL. The tubes were then inoculated with the test microorganisms grown in their suitable broth at 37 °C for 24 h (1 × 10^8^ CFU/mL for bacteria and 1 × 10^6^ CFU/mL of yeast), 0.1 mL of the above inoculum was administered per 5 mL, and the tubes were incubated at 37 °C for 24 h. The lowest concentration showing no growth was taken as the MIC.

### 3.4. Anti-Inflammatory Activity

#### 3.4.1. Proteinase-Inhibitory Activity

The test was performed according to El-Serwy et al. [32] with minor modifications. The reaction mixture (2 mL) contained 0.06 mg trypsin, 20 mM Tris HCl buffer (pH 7.4), and 1 mL test sample (250 µg/mL for each compound). After incubating for 5 min, 1 mL of 0.8% (*w*/*v*) casein was added. The mixture was incubated for an additional 20 min. The reaction was arrested by adding 1 mL of 10% perchloric acid. The cloudy suspension was centrifuged, and the absorbance of the supernatant was read at 280 nm. Reference standard aspirin was used as the positive control. The experiment was performed in triplicate. The percent inhibition of protein denaturation was calculated using the following Equation (2):

(2) $$\%\;Inhibition=100-\left[\frac{\left(A\;\mathrm{control}\;-A\;\mathrm{test}\right)}{A\;\mathrm{control}}\times 100\right]$$

where Acontrol and Atest represent the absorbance of the control reaction without and with the sample, respectively.

#### 3.4.2. Pharmacological In Vivo Study

Prophylactic anti-inflammatory effect on formaldehyde-induced rat paw oedema:

Animals: Female Wister albino rats (150–175 g) were obtained from the animal house colony of the National Research Centre, Egypt. The animals were kept in standard plastic cages in an air-conditioned room at 22 ± 3 °C and 55 ± 5% humidity and supplied with a standard laboratory diet and water ad libitum. All animal experimental procedures were carried out according to the Ethics Committee of the National Research Centre, Cairo, Egypt and followed the guidelines of the National Institutes of Health Guide for Care and Use of Laboratory Animals.

Tested derivatives and the reference drug were orally administered to rats one hour before induction of paw oedema and all hind footpad thickness were measured at zero time (basal reading) immediately before, then every hour for three consecutive hours, after induction of left hind paw oedema. The paw thickness was measured by using Vernier calliper [33].

The difference between initial and subsequent readings gave the change in oedema thickness for the corresponding time. Oedema volume of the paw of the positive control group (Vc) and volume of treated group (Vt) were used to calculate percentage (%) inhibition and (%) oedema volume using the following Equations (3) and (4):

% Oedema volume = (Oedema volume after drug treatment/base time volume) × 100 − 100(3)

% Inhibition = (Vt/Vc) × 100 − 100(4)

Thirty-six female rats were classified into the following groups (6 rats each):

- Positive control group: Paw oedema was induced by 0.2 mL (1%, *w*/*v*) formaldehyde injected in the subplantar area of the left hind paw of the rat [33].
- Prophylactic groups:

All drug regimens were given orally one hour before induction of paw oedema, as follows:

(a) Indomethacin (Reference) group: Rats were given indomethacin orally at 25 mg/kg [44].
(b) Test groups: rats were given coumarin derivative **2b**, **3a**, **7f**, and **8c** orally in a dose of 2.5 mg/kg each.

Statistical analysis: values were expressed as means ± standard error (SE). Comparisons between means were carried out using two-way analysis of variance followed by the Tukey Kramer multiple comparisons test for all acute toxicity tests. *p* < 0.0001 was considered significant. All statistical tests were performed in GraphPad Prism (version 6).

#### 3.4.3. COX Inhibition

COX assay kits were purchased from Cayman Chemical (Ann Arbor, MI, USA) and used following the manufacturer’s instructions (COX-1 Catalogue no. 701070, COX-2 Catalogue no. 701080). The activity was expressed in IC_50_, which is the mean of two determinations, and the deviation from the mean is <10% of the mean value.

### 3.5. In Silico Studies

#### 3.5.1. Molecular Docking Study

The protein crystal structure was obtained from the RCSB Protein Databank. The 3D-crystal structure used for docking was 5KIR, cyclooxygenase-2 (prostaglandin synthase-2) complexed with the selective inhibitor rofecoxib, with a resolution of 2.697 Å. Discovery Studio, AutoDock Tools, Vina, and PyRx (The Scripps Research Institute, La Jolla, CA, USA) software programs were used for molecular docking. Using Discovery Studio, the protein crystal structure was processed by removing water, ligands, and sulphate molecules. The protein was then saved as a PDB file format. Second, polar hydrogens were added using AutoDock Tools, and the file was saved in PDBQT format. Third, the original ligand was isolated and saved as a PDB using Discovery Studio. Compounds **5a** and **8d** were also saved in PDB file format. Finally, docking was performed by PyRx, and the docking scores were obtained.

#### 3.5.2. Drug-Likeness Assessment

In silico Lipinski Rule of Five (RO5) and topological polar surface area (TPSA) analyses were conducted using Molinspiration (accessed 24 February 2018) [39], an online cheminformatics software that provides web-based interactive calculation of molecular properties, including molecular weight, hydrogen bond donors and acceptors, and calculated partition coefficients of the molecules.

## 4. Conclusions

In the present investigation, the newly synthesised compounds were screened for antioxidant, antimicrobial, and anti-inflammatory activities. Compounds **8a–d** demonstrated the highest antioxidant activity, while compounds **7c**,**d**, **8c**,**d**, and **9c**,**d** exhibited antimicrobial activity equal to or higher than the standard antimicrobials of ciprofloxacin and ketoconazole against one or more tested microorganisms. Notably, compound **7d** exhibited broad-spectrum antimicrobial activity. Regarding anti-inflammatory activity, compounds **2b**, **3a**,**b**, **5c**, and **9a** showed more potent in vitro antiproteinase activity than aspirin; however, **5d**,**e**, **7f**, and **8a**,**c** were as potent as aspirin. Moreover, compound **3a** showed significant in vivo anti-inflammatory activity. Compounds **5a** and **8d** were the most selective and potent coumarin derivatives, respectively, toward COX-2 isozyme. These potent compounds, except **5c**, **7c**, and **9c**, met the in silico criteria for orally active drugs. Potent compounds such as **3a**, **8c**,**d**, and **7d** are promising starting points for the development of more potent antioxidant, antimicrobial, and/or anti-inflammatory agents as orally active compounds. However, further studies are needed to determine the toxicity and pharmacokinetic properties of these compounds.

## Figures and Tables

**Table 1:** Antioxidant activity of coumarin compounds and ascorbic acid using DPPH antioxidant assay (IC_50_, μg/mL).

Compd	IC_50_	Compd	IC_50_	Compd	IC_50_
**2a**	—	**5f**	—	**8d**	4.30 ± 0.531
**2b**	—	**7a**	93.12 ± 0.727	**8e**	32.85 ± 1.322
**3a**	—	**7b**	61.78 ± 2.719	**8f**	35.36 ± 3.265
**3b**	48.38 ± 4.616	**7c**	120.12 ± 4.629	**9a**	—
**4a**	—	**7d**	112.19 ± 4.391	**9b**	—
**4b**	—	**7e**	71.67 ± 3.231	**9c**	—
**5a**	—	**7f**	83.52 ± 1.849	**9d**	—
**5c**	—	**8a**	14.51 ± 1.827	**9e**	—
**5d**	82.92 ± 3.300	**8b**	19.25 ± 4.171	**9f**	—
**5e**	—	**8c**	3.87 ± 0.409	Ascorbic acid *	2.83 ± 0.166

Values represent mean ± SD (n = 3). (—): inactive (IC_50_ > 200 μg/mL). * Positive control. DPPH, 2,2-diphenyl-1-picrylhydrazyl; IC_50_, half maximal inhibitory concentration; Compd, compound.

**Table 2:** Antimicrobial activity expressed as the diameter (mm) of inhibition zones of chemical compounds (except **5b**), ciprofloxacin, and ketoconazole against the pathological strains *S. aureus* ATCC 29,213 (*SA*), *B. subtilis* ATCC 6633 (*BS*)*, B. megaterium* ATCC 9885 (*BM*), *E. coli* ATCC 2592 (*EC*), *P. aeruginosa* ATCC 27,953 (*PA*), *S. cerevisiae* local isolate (*SC*), and *C. albicans* NRRL Y-477 (*CA*) based on well diffusion assay.

Compd	Consensus Log *P* ^a^	Gram +ve Bacteria			Gram −ve Bacteria		Fungi	
		*SA*	*BS*	*BM*	*EC*	*PA*	*SC*	*CA*
**2a**	2.5	24	22	25	18	20	25	22
**2b**	2.39	25	23	25	18	21	27	23
**3a**	1.76	-	18	16	16	15	-	-
**3b**	1.75	16	-	-	16	16	17	18
**4a**	3.03	16	-	-	-	-	-	-
**4b**	2.94	16	-	-	15	15	-	-
**5a**	4.29	25	27	22	26	24	26	25
**5c**	4.21	23	23	24	24	25	27	24
**5d**	4.21	18	-	-	-	-	19	19
**5e**	5.33	16	16	16	15	15	22	20
**5f**	5.28	25	23	20	20	21	24	22
**7a**	1.23	28	29	25	28	27	26	26
**7b**	1.05	-	-	-	-	-	-	-
**7c**	1.88	30	31	30	30	24	31	27
**7d**	1.71	30	31	30	28	25	32	30
**7e**	2.51	23	25	25	20	22	20	23
**7f**	2.36	24	27	27	23	24	26	24
**8a**	2.52	26	28	29	25	26	28	29
**8b**	2.41	20	19	24	18	17	19	20
**8c**	2.03	30	29	28	27	29	32	30
**8d**	1.90	30	29	29	31	31	32	30
**8e**	2.45	-	15	16	-	-	-	-
**8f**	2.32	16	15	15	-	-	-	-
**9a**	3.5	16	18	18	-	-	-	-
**9b**	3.15	22	24	25	26	26	25	23
**9c**	4.48	28	29	30	30	30	31	30
**9d**	4.32	28	29	29	38	29	32	30
**9e**	3.88	23	21	20	21	21	29	23
**9f**	3.73	25	25	26	26	28	26	24
**Ciprofloxacin**		28	30	30	30	30	-	-
**Ketoconazole**		-	-	-	-	-	30	28

(-): inactive (IZ < 15 mm); Compd, compound. ^a^ Calculated using SwissADME web tool. Consensus Log *P* is the average of five different prediction methods for the calculation of computerised partition coefficient, including ilog *P*, Xlog *P*3, Wlog *P*, Mlog *P*, and Log *P*_SILICOS-IT_.

**Table 3:** Minimum inhibitory concentration (μg/mL) of the most active compounds (having inhibition zones > 16 mm), ciprofloxacin, and ketoconazole against the pathological strains *S. aureus* ATCC 29,213 (*SA*), *B. subtilis ATCC6633* (*BS*), *B. megaterium ATCC* 9885 (*BM*), *E. coli* ATCC *2592* (*EC*), *P. aeruginosa* ATCC 27,953 (*PA*), *S. cerevisiae* local isolate (*SC*), and *C. albicans* NRRL Y-477 (*CA*) based on two-fold serial dilution technique.

Compd	Gram +ve Bacteria			Gram –ve Bacteria		Fungi	
	*SA*	*BS*	*BM*	*EC*	*PA*	*SC*	*CA*
**2a**	500	500	500	1000	500	500	500
**2b**	500	500	500	1000	1000	500	500
**3a**	N.D.	1000	N.D.	N.D.	N.D.	N.D.	N.D.
**3b**	N.D.	N.D.	N.D.	N.D.	N.D.	1000	1000
**5a**	250	250	500	250	500	500	500
**5c**	500	500	500	500	500	500	500
**5d**	1000	N.D.	N.D.	N.D.	N.D.	1000	1000
**5e**	N.D.	N.D.	N.D.	N.D.	N.D.	500	500
**5f**	500	500	1000	1000	1000	500	500
**7a**	250	250	500	250	250	250	250
**7c**	125	125	125	125	250	125	125
**7d**	125	125	125	125	125	125	125
**7e**	500	500	500	500	1000	1000	500
**7f**	250	250	250	500	500	250	500
**8a**	500	250	250	500	500	250	250
**8b**	1000	1000	1000	1000	1000	1000	1000
**8c**	125	125	250	250	250	125	125
**8d**	125	125	250	125	125	125	125
**9a**	N.D.	1000	1000	N.D.	N.D.	N.D.	N.D.
**9b**	500	500	500	250	250	500	500
**9c**	250	250	250	250	250	125	125
**9d**	250	250	250	250	250	125	125
**9e**	500	500	500	500	500	250	500
**9f**	500	500	250	250	250	500	500
**Ciprofloxacin**	125	125	125	125	125	N.D.	N.D.
**Ketoconazole**	N.D.	N.D.	N.D.	N.D.	N.D.	125	125

N.D., not determined; Compd, compound.

**Table 4:** Proteinase-inhibitory activity of the coumarin compounds and aspirin.

Compd	% Inhibition	Compd	% Inhibition	Compd	% Inhibition
**2a**	17.38 ± 1.63	**5f**	26.83 ± 5.33	**8d**	20.15 ± 7.31
**2b**	74.68 ± 3.01	**7a**	18.78 ± 2.05	**8e**	18.5 ± 3.47
**3a**	79.72 ± 4.51	**7b**	5.93 ± 2.11	**8f**	2.0 ± 1.4
**3b**	49.28 ± 10.93	**7c**	8.9 ± 2.04	**9a**	62.14 ± 4.87
**4a**	31.99 ± 2.45	**7d**	36.84 ± 4.87	**9b**	—
**4b**	33.17 ± 2.79	**7e**	19.8 ± 2.54	**9c**	17.6 ± 6.86
**5a**	N.D.	**7f**	43.88 ± 5.6	**9d**	31.88 ± 3.82
**5c**	68.5 ± 6.23	**8a**	44.7 ± 8.22	**9e**	22.83 ± 4.88
**5d**	43.4 ± 1.52	**8b**	14.3 ± 2.21	**9f**	21.7 ± 7.6
**5e**	45.78 ± 1.62	**8c**	41.69 ± 2.83	Aspirin *	45.83 ± 4.21

Values represent mean ± SD (n = 3). (—): inactive. N.D., not determined; Compd, compound. * Positive control.

**Table 5:** In vitro COX-inhibitory activity of compounds **2–8**.

Compd	IC_50_ (µM) ^a,b^		SI ^c^
	COX-1	COX-2	
**2a**	116.66	2.74	42.58
**2b**	201.16	4.25	47.33
**3a**	183.27	9.81	18.68
**3b**	7.73	2.45	3.16
**4a**	12.49	7.99	1.56
**4b**	57.46	25.23	2.28
**5a**	1126.47	7.41	152.02
**5b**	215.39	19.63	10.97
**5c**	18.50	13.30	1.39
**5d**	27.19	16.04	1.7
**5e**	20.80	8.27	2.52
**5f**	90.20	3.10	29.1
**7a**	239.47	17.42	13.75
**7b**	47.60	8.24	5.78
**7c**	62.27	19.94	3.12
**7d**	214.28	3.35	63.96
**7e**	29.62	16.11	1.84
**7f**	40.89	10.07	4.06
**8a**	25.02	8.50	2.94
**8b**	25.99	13.37	1.94
**8c**	15.40	11.90	1.29
**8d**	19.44	1.86	10.45
**8e**	19.90	9.22	2.16
**8f**	7.92	25.66	0.31
**Indomethacin**	1.88	15.14	0.12
**Celecoxib**	187.82	0.77	243.92

^a^ The mean of two determinations. ^b^ The deviation from the mean is <10% of the mean value. ^c^ Selectivity index (COX-1/COX-2).

**Table 6:** The Lipinski parameters and topological polar surface area (TPSA) of target compounds.

Compd	Mol. Wt	miLog*P*	n-ON	n-OHNH	RO5 Violations	TPSA (Å^2^)
**Rule**	≤500	≤5	≤10	≤5	≤1	≤140
**2** **a**	375.34	3.10	8	2	0	135.08
**2** **b**	320.30	2.40	6	2	0	102.40
**3** **a**	393.36	2.05	9	4	0	154.39
**3** **b**	338.32	1.35	7	4	0	121.70
**4** **a**	430.42	3.58	9	0	0	124.66
**4** **b**	375.38	2.88	7	0	0	91.98
**5** **a**	463.45	5.16	8	0	1	121.42
**5** **b**	408.41	4.45	6	0	0	88.74
**5** **c**	553.53	4.79	11	0	2	149.13
**5** **d**	498.49	4.08	9	0	0	116.44
**5** **e**	532.34	6.44	8	0	2	121.42
**5** **f**	477.30	5.74	6	0	1	88.74
**7** **a**	430.85	1.76	9	4	0	156.77
**7** **b**	410.43	1.53	9	4	0	156.77
**7** **c**	508.92	2.01	11	2	2	168.56
**7** **d**	488.50	1.78	11	3	1	168.56
**7** **e**	513.96	2.89	10	3	1	155.67
**7** **f**	493.54	2.66	10	3	0	155.67
**8** **a**	351.77	3.07	6	2	0	96.61
**8** **b**	331.35	2.84	6	2	0	96.61
**8** **c**	367.77	2.59	7	3	0	116.84
**8** **d**	347.35	2.36	7	3	0	116.84
**8** **e**	393.80	2.97	7	2	0	113.68
**8** **f**	373.39	2.74	7	2	0	113.68
**9** **a**	526.91	4.90	10	2	1	159.50
**9** **b**	506.49	4.67	10	2	1	159.50
**9** **c**	516.36	5.62	7	2	2	113.68
**9** **d**	495.94	5.39	7	2	1	113.68
**9** **e**	511.94	5.00	8	2	2	122.91
**9** **f**	491.52	4.77	8	2	0	122.91

(n-OHNH), H-bond donors; (n-ON), H-bond acceptors; Compd, compound; Mol. Wt, molecular weight; RO5, rule of five.

## Supplementary Materials

Figures S1–S16: ^1^H and ^13^C-NMR spectra of compounds **2a**, **4a**, **5c**, **6b**, **8e**, and **9f**. Table S1: X-ray Crystallographic data of compound **2a.**

## References

[1] Liguori I., Russo G., Curcio F., Bulli G., Aran L., Della-Morte D., Gargiulo G., Testa G., Cacciatore F., Bonaduce D. (2018). Oxidative Stress, Aging, and Diseases. Clin. Interv. Aging.

[2] Chen L., Deng H., Cui H., Fang J., Zuo Z., Deng J., Li Y., Wang X., Zhao L. (2017). Inflammatory Responses and Inflammation-Associated Diseases in Organs. Oncotarget.

[3] Pal C., Bengtsson-Palme J., Kristiansson E., Larsson D.G.J. (2016). The Structure and Diversity of Human, Animal and Environmental Resistomes. Microbiome.

[4] Laxminarayan R., Duse A., Wattal C., Zaidi A.K.M., Wertheim H.F.L., Sumpradit N., Vlieghe E., Hara G.L., Gould I.M., Goossens H. (2013). Antibiotic Resistance—the Need for Global Solutions. Lancet Infect. Dis..

[5] Pereira T.M., Franco D.P., Vitorio F., Kummerle A.E. (2018). Coumarin Compounds in Medicinal Chemistry: Some Important Examples from the Last Years. Curr. Top. Med. Chem..

[6] Kontogiorgis C., Detsi A., Hadjipavlou-Litina D. (2012). Coumarin-Based Drugs: A Patent Review (2008–Present). Expert Opin. Ther. Pat..

[7] Dehkordi M.F., Dehghan G., Mahdavi M., Hosseinpour Feizi M.A. (2015). Multispectral Studies of DNA Binding, Antioxidant and Cytotoxic Activities of a New Pyranochromene Derivative. Spectrochim. Acta. A. Mol. Biomol. Spectrosc..

[8] Vukovic N., Sukdolak S., Solujic S., Niciforovic N. (2010). Substituted Imino and Amino Derivatives of 4-Hydroxycoumarins as Novel Antioxidant, Antibacterial and Antifungal Agents: Synthesis and in Vitro Assessments. Food Chem..

[9] Arora R.K., Kaur N., Bansal Y., Bansal G. (2014). Novel Coumarin–Benzimidazole Derivatives as Antioxidants and Safer Anti-Inflammatory Agents. Acta Pharm. Sin. B.

[10] Bylov I.E., Vasylyev M.V., Bilokin Y.V. (1999). Synthesis and Anti-Inflammatory Activity of N-Substituted 2-Oxo-2H-1-Benzopyran-3-Carboxamides and Their 2-Iminoanalogues. Eur. J. Med. Chem..

[11] Sashidhara K.V., Kumar M., Modukuri R.K., Sonkar R., Bhatia G., Khanna A.K., Rai S., Shukla R. (2011). Synthesis and Anti-Inflammatory Activity of Novel Biscoumarin–Chalcone Hybrids. Bioorg. Med. Chem. Lett..

[12] Bedair A.H., El-Hady N.A., El-Latif M.S.A., Fakery A.H., El-Agrody A.M. (2000). 4-Hydroxycoumarin in Heterocyclic Synthesis: Part III. Synthesis of Some New Pyrano[2,3-d]Pyrimidine, 2-Substituted[1,2,4]Triazolo[1,5-c]Pyrimidine and Pyrimido[1,6-b][1,2,4]Triazine Derivatives. Il Farm..

[13] Shi X., Lv C., Li J., Hou Z., Yang X., Zhang Z., Luo X., Yuan Z., Li M. (2015). Synthesis, Photoluminescent, Antibacterial Activities and Theoretical Studies of Three Novel Coumarin and Dihydropyran Derivatives Containing a Triphenylamine Group. Res. Chem. Intermed..

[14] Al-Masoudi N.A., Mohammed H.H., Hamdy A.M., Akrawi O.A., Eleya N., Spannenberg A., Pannecouque C., Langer P. (2014). Synthesis and Anti-HIV Activity of New Fused Chromene Derivatives Derived from 2-Amino-4-(1-Naphthyl)-5-Oxo-4H,5H-Pyrano[3,2- c]Chromene-3-Carbonitrile. Z. Für Naturforschung B.

[15] Sahoo J., Kumar Mekap S., Sudhir Kumar P. (2015). Synthesis, Spectral Characterization of Some New 3-Heteroaryl Azo 4-Hydroxy Coumarin Derivatives and Their Antimicrobial Evaluation. J. Taibah Univ. Sci..

[16] Alshibl H.M., Al-Abdullah E.S., Alkahtani H.M. (2019). Coumarin: A Promising Scaffold for Design and Development of Bioactive Agents. Curr. Bioact. Compd..

[17] Silverstein F.E., Faich G., Goldstein J.L., Simon L.S., Pincus T., Whelton A., Makuch R., Eisen G., Agrawal N.M., Stenson W.F. (2000). Gastrointestinal Toxicity With Celecoxib vs. Nonsteroidal Anti-Inflammatory Drugs for Osteoarthritis and Rheumatoid Arthritis The CLASS Study: A Randomized Controlled Trial. JAMA.

[18] Peppercorn M.A. (1984). Sulfasalazine. Pharmacology, Clinical Use, Toxicity, and Related New Drug Development. Ann. Intern. Med..

[19] Fischi M.A., Dickinson G.M., Vole L.L. (1988). Safety and Efficacy of Sulfamethoxazole and Trimethoprim Chemoprophylaxis for Pneumocystis Carinii Pneumonia in AIDS. JAMA.

[20] Fox C.L. (1977). Pharmacology and Clinical Use of Silver Sulfadiazine and Related Topical Antimicrobial Agents. Pahlavi Med. J..

[21] Alshibl H.M., Al-Abdullah E.S., Haiba M.E. (2019). Coumarin Derivatives. U.S. Patent.

[22] Kiyani H., Ghorbani F. (2015). Efficient Tandem Synthesis of a Variety of Pyran-Annulated Heterocycles, 3,4-Disubstituted Isoxazol-5(4H)-Ones, and α,β-Unsaturated Nitriles Catalyzed by Potassium Hydrogen Phthalate in Water. Res. Chem. Intermed..

[23] Reddy N.S., Mallireddigari M.R., Cosenza S., Gumireddy K., Bell S.C., Reddy E.P., Reddy M.V.R. (2004). Synthesis of New Coumarin 3-(N-Aryl) Sulfonamides and Their Anticancer Activity. Bioorg. Med. Chem. Lett..

[24] Behrami A. (2014). Antibacterial Activity of Coumarine Derivatives Synthesized from 4-Chloro-Chromen-2-One. The Comparison with Standard Drug. Orient. J. Chem..

[25] Yu L., Haley S., Perret J., Harris M., Wilson J., Qian M. (2002). Free Radical Scavenging Properties of Wheat Extracts. J. Agric. Food Chem..

[26] Qazi S.S., Li D., Briens C., Berruti F., Abou-Zaid M.M. (2017). Antioxidant Activity of the Lignins Derived from Fluidized-Bed Fast Pyrolysis. Mol. Basel Switz..

[27] Perez-Eid C., Pauli W.M., Bazerque P. (1990). An Antibiotic Assay by Agar-Well Diffusion Method. Acta Biologiae et Med. Experimentalis..

[28] Scott A.C., Collee J., Duguid J., Fraser A., Marmion B. (1989). Laboratory Control of Antimicrobial Therapy. Practical Medical Microbiology.

[29] Rutkowska E., Pajak K., Jóźwiak K. (2013). Lipophilicity-Methods of Determination and Its Role in Medicinal Chemistry. Acta Pol. Pharm..

[30] Kalinowska M., Bajko E., Matejczyk M., Kaczyński P., Łozowicka B., Lewandowski W. (2018). The Study of Anti-/Pro-Oxidant, Lipophilic, Microbial and Spectroscopic Properties of New Alkali Metal Salts of 5-O-Caffeoylquinic Acid. Int. J. Mol. Sci..

[31] Kocabalkanli A., Ates Ö., Ötük G. (2001). Synthesis of Mannich Bases of Some 2,5-Disubstituted 4-Thiazolidinones and Evaluation of Their Antimicrobial Activities. Arch. Pharm. (Weinheim).

[32] El-Serwy W.S., Mohamed N.A., El-Serwy W.S., Mahmoud A.H. (2017). Synthesis, biological evaluation and molecular modeling studies of some 5-methylisoxazole derivatives as anti-Inflammatory agents. J. Chem. Pharmaceut. Res..

[33] Ibrahim B.M.M., Yassin N.A.Z., Hetta M.H., Ta K.F., Mohammed W.I., Hassan M.E.S. (2016). Phytochemical and pharmacological studies on newly-suggested herbal formulations for potential protection against inflammatory conditions. Int. J. Pharmacogn. Phytochem. Res..

[34] Owen C.A., Campbell E.J. (1995). Neutrophil Proteinases and Matrix Degradation. The Cell Biology of Pericellular Proteolysis. Semin. Cell Biol..

[35] Leelaprakash G., Dass S.M. (2011). In vitro anti-inflammatory activity of methanol extract of Enicostemma axillare. Int. J. Drug Dev. Res..

[36] Ruiz-Ruiz J.C., Matus-Basto A.J., Acereto-Escoffié P., Segura-Campos M.R. (2017). Antioxidant and Anti-Inflammatory Activities of Phenolic Compounds Isolated from Melipona Beecheii Honey. Food Agric. Immunol..

[37] Garavito R.M., DeWitt D.L. (1999). The Cyclooxygenase Isoforms: Structural Insights into the Conversion of Arachidonic Acid to Prostaglandins. Biochim. Biophys. Acta Mol. Cell Biol. Lipids.

[38] Llorens O., Perez J.J., Palomer A., Mauleon D. (1999). Structural Basis of the Dynamic Mechanism of Ligand Binding to Cyclooxygenase. Bioorg. Med. Chem. Lett..

[39] Molinspiration Cheminformatics. http://www.molinspiration.com.

[40] Lipinski C.A., Lombardo F., Dominy B.W., Feeney P.J. (2001). Experimental and Computational Approaches to Estimate Solubility and Permeability in Drug Discovery and Development Settings. Adv. Drug Deliv. Rev..

[41] Clark D.E. (1999). Rapid Calculation of Polar Molecular Surface Area and Its Application to the Prediction of Transport Phenomena. 2. Prediction of Blood–Brain Barrier Penetration. J. Pharm. Sci..

[42] Bhatt N.S., Shah A.K., Raval R.V., Thakor V. (1984). Novel Synthesis of Benzofurans. Curr. Sci..

[43] Kapewangolo P., Omolo J.J., Bruwer R., Fonteh P., Meyer D. (2015). Antioxidant and Anti-Inflammatory Activity of Ocimum Labiatum Extract and Isolated Labdane Diterpenoid. J. Inflamm. Lond. Engl..

[44] Moharram F.A.-E., Al-Gendy A.A., El-Shenawy S.M., Ibrahim B.M., Zarka M.A. (2018). Phenolic profile, anti-inflammatory, antinociceptive, anti-ulcerogenic and hepatoprotective activities of Pimenta racemosa leaves. BMC Complement Altern Med..

