# Supplementary material for: Synthesis and Evaluation of New Coumarin Derivatives as Antioxidant, Antimicrobial, and Anti-Inflammatory Agents
Source: Molecules. 2020 Jul 16;25(14):3251. doi: 10.3390/molecules25143251 (PMC7397269; doi:10.3390/molecules25143251)
Supplement: Supplementary file 1 [file molecules-25-03251-s001.pdf]

Supplimentary Material

Synthesis and evaluation of new coumarin derivatives as antioxidant, antimicrobial, and anti-inflammatory agents

Hanan M. Alshibl [1,*], Ebtehal S. Al-Abdullah [1], Mogedda E. Haiba [1,2], Hamad M. Alkahtani [1], Ghada E.A. Awad [3], Ahlam H. Mahmoud [4], Bassant M.M. Ibrahim [5], Ahmed Bari [1], Alexander Villinger [6]

[1] Department of Pharmaceutical Chemistry, College of Pharmacy, King Saud University, Riyadh 11451, Saudi Arabia; halshibl@ksu.edu.sa (H.M.A.S.); ealabdullah@ksu.edu.sa (E.S.A.A.) ; mogedda.haiba@yahoo.com (M.E.H.); ahamad@ksu.edu.sa (H.M.A.K.); abari@ksu.edu.sa (A.B.)

[2] Department of Medicinal Chemistry, National Research Centre, Cairo 12622, Egypt

[3] Chemistry of Natural and Microbial Product Department, National Research Centre, Cairo 12622, Egypt; ghadaawadnrc@gmail.com

[4] Department of Therapeutic Chemistry, Pharmaceutical and Drug Industries Research Division, National Research Centre, Dokki, Cairo 12622, Egypt; ahlam@hotmail.co.uk

[5] Pharmacology Department, Medical Research Division, National Research Centre, Cairo 12622, Egypt; bmmih1974@gmail.com

[6] Institut für Chemie, Abteilung Anorganische Chemie, Universität Rostock, Albert-Einstein-Str. 3a, 18059 Rostock, Germany; alexander.villinger@uni-rostock.de

* Correspondence: Halshibl@ksu.edu.sa ; Tel.: +966-11805-2756.

Contents:

1- NMR spectra of compounds (**2a**, **4a**, **5c**, **6b**, **8e**, **9f**)
2- X-ray Crystallographic data of Compound **2a**

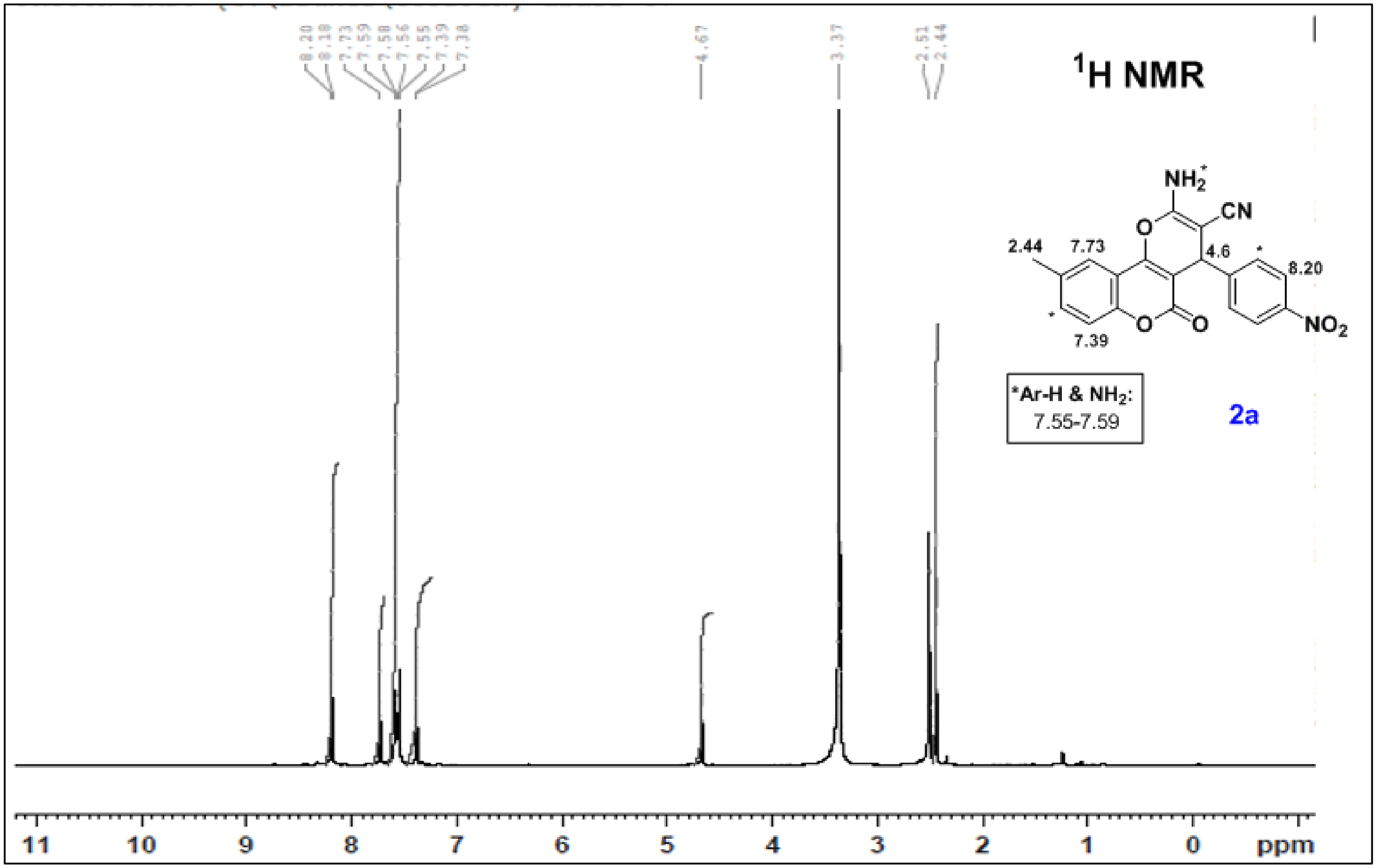

Figure S1: ^{1}H-NMR spectrum of compound **2a** in DMSO-d$_6$ (700 MHz).

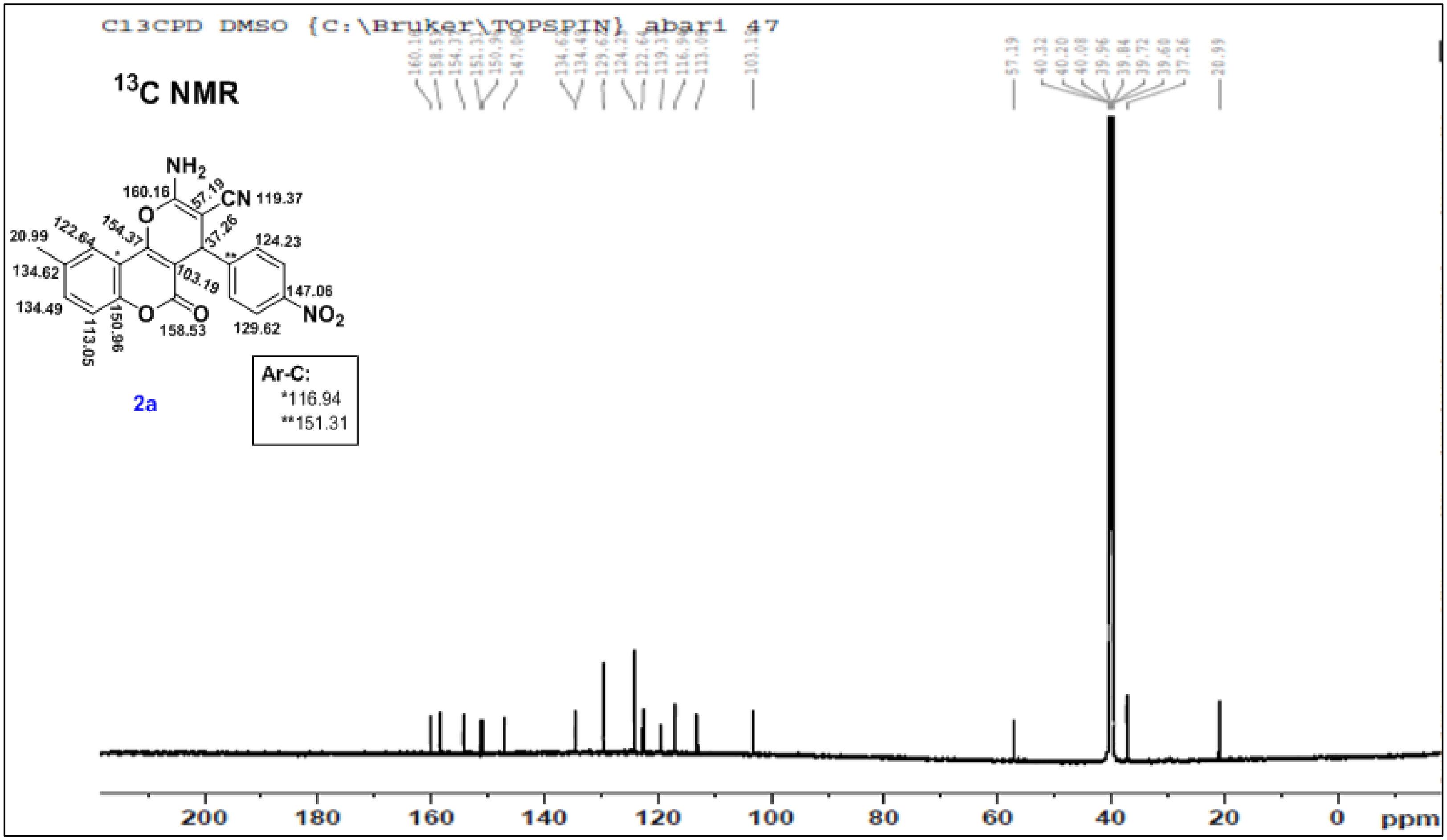

Figure S2: ^{13}C-NMR spectrum of compound **2a** in DMSO-d$_6$ (176 MHz).

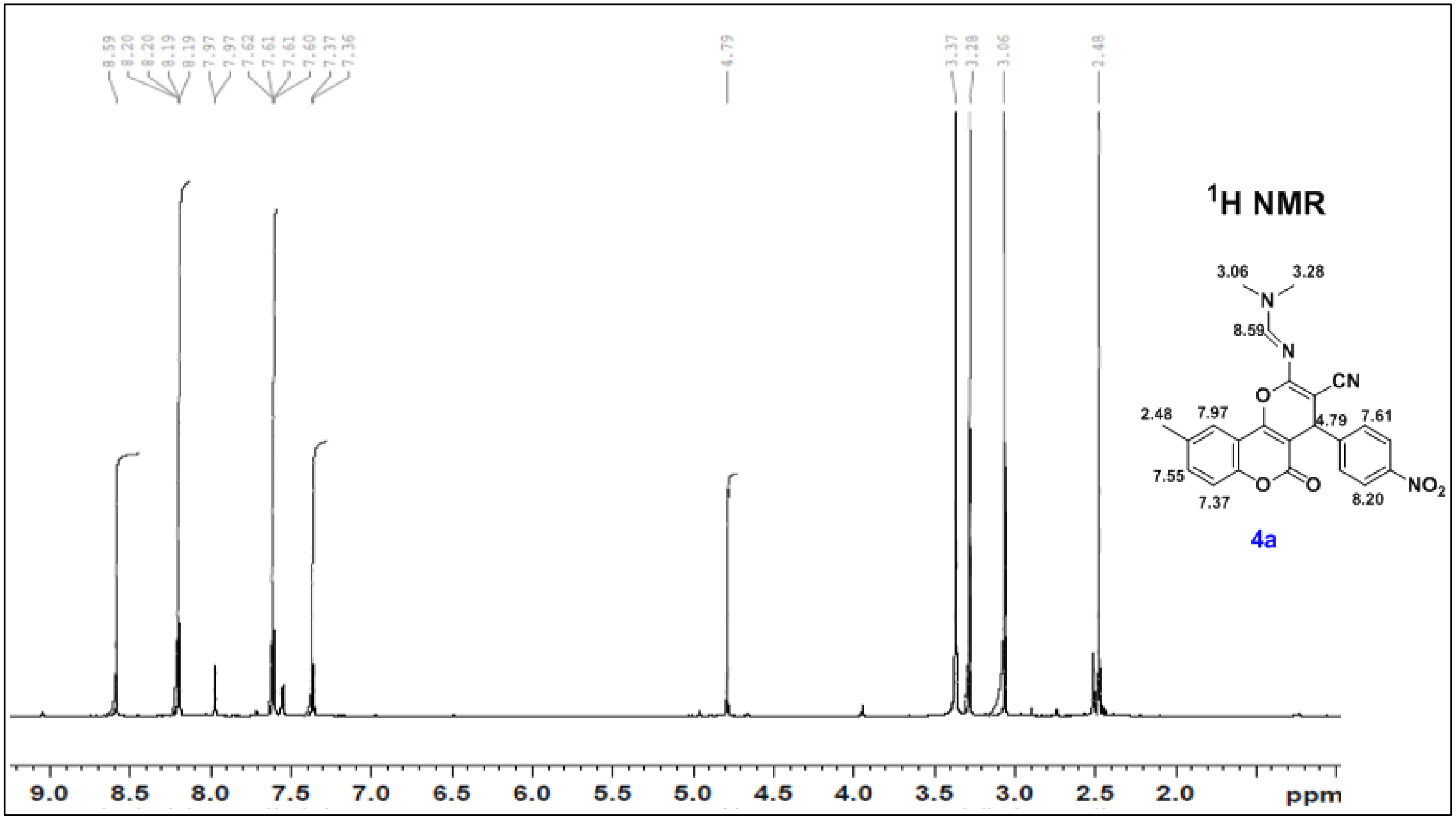

Figure S3: ^{1}H-NMR spectrum of compound **4a** in DMSO-d_6 (700 MHz).

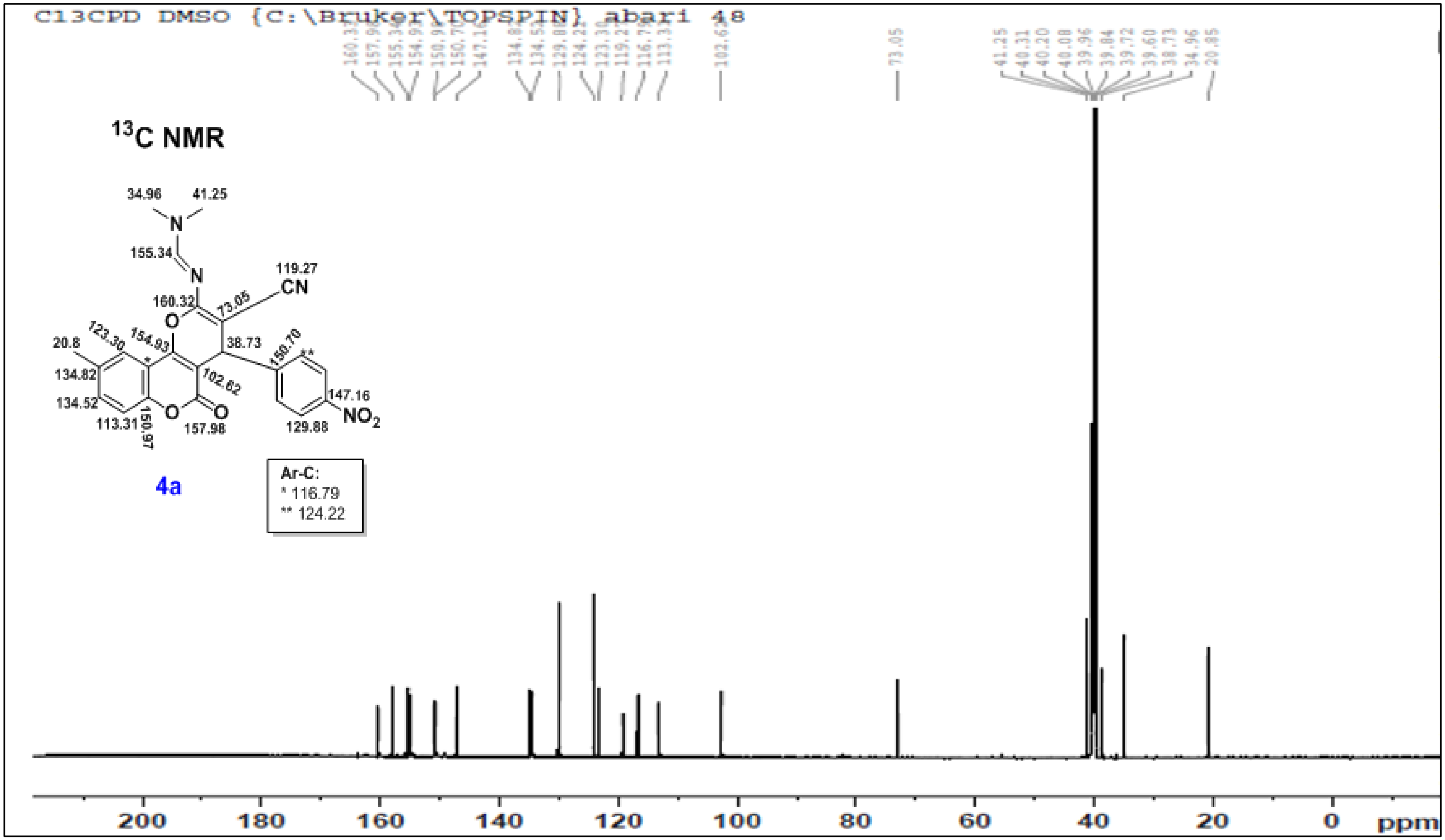

Figure S4: ^{13}C-NMR spectrum of compound **4a** in DMSO-d_6 (176 MHz).

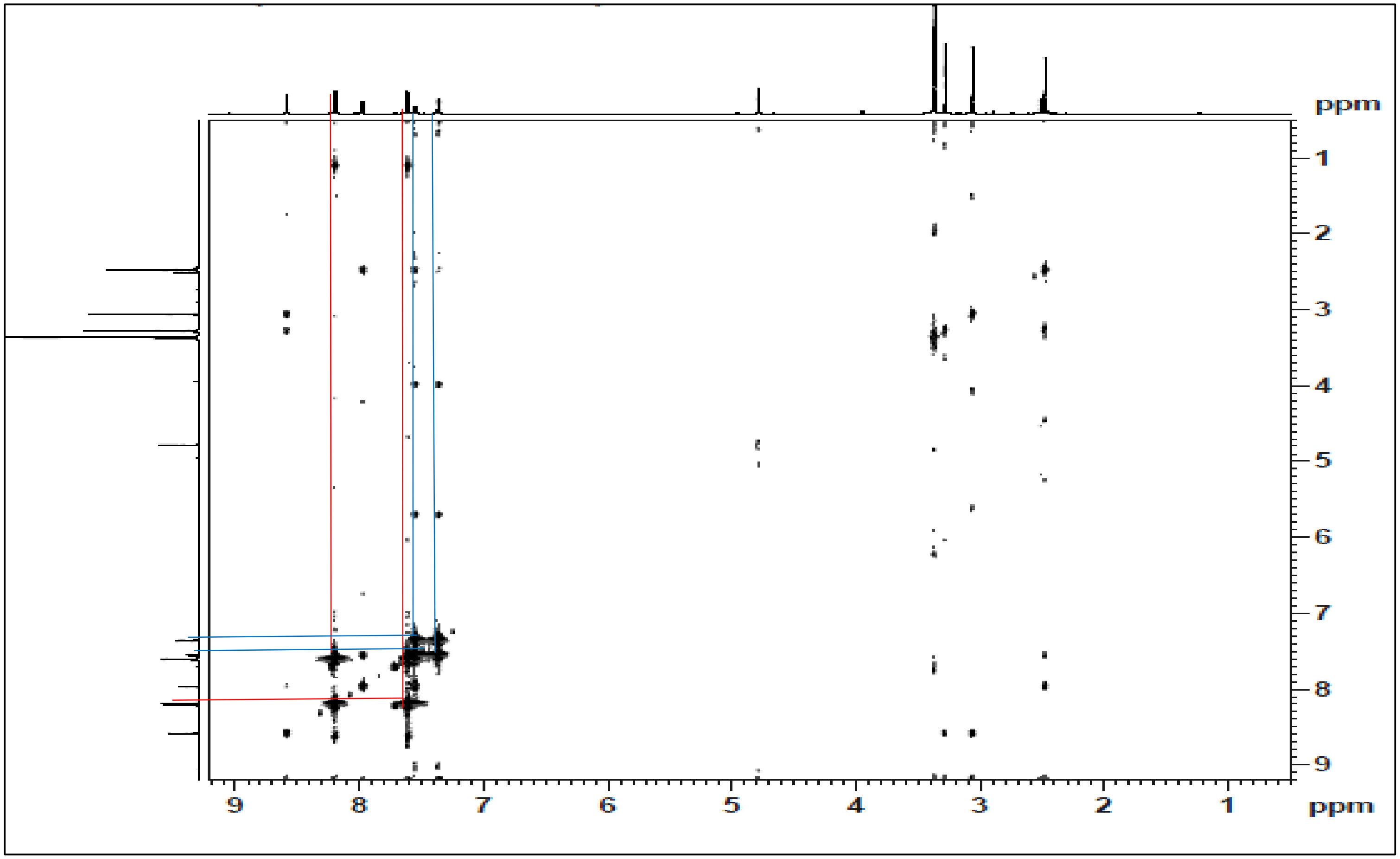

Figure S5: ^{1}H-^{1}H-Homonuclear COSY NMR spectrum of compound **4a** in DMSO-d_6 (700 MHz).

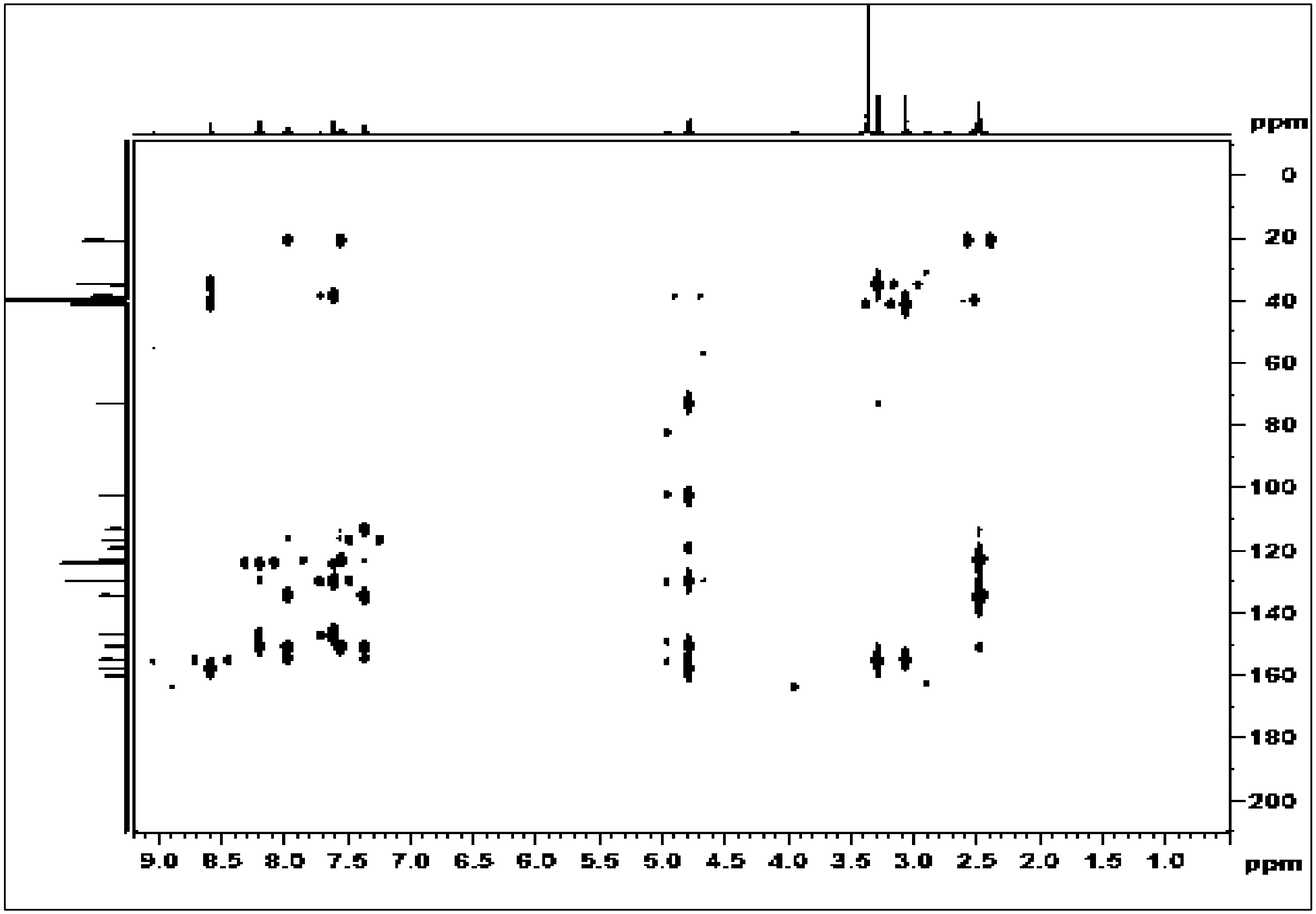

Figure S6: ^{1}H-^{13}C-Heteronuclear COSY (HMBC) NMR spectrum of compound **4a** in DMSO-d_6 (700/176 MHz).

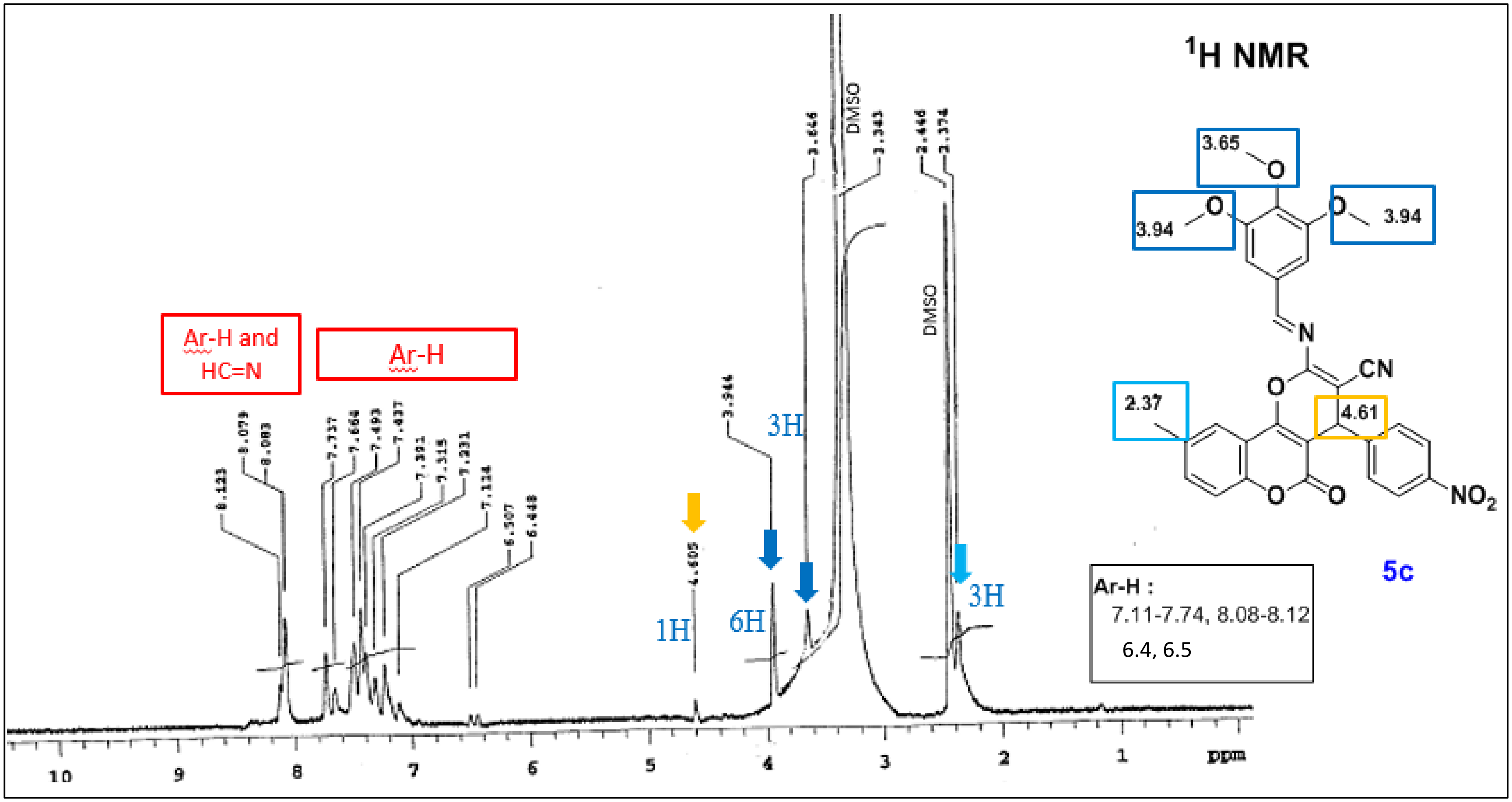

Figure S7: ^{1}H-NMR spectrum of compound **5c** in DMSO-d_6 (600 MHz).

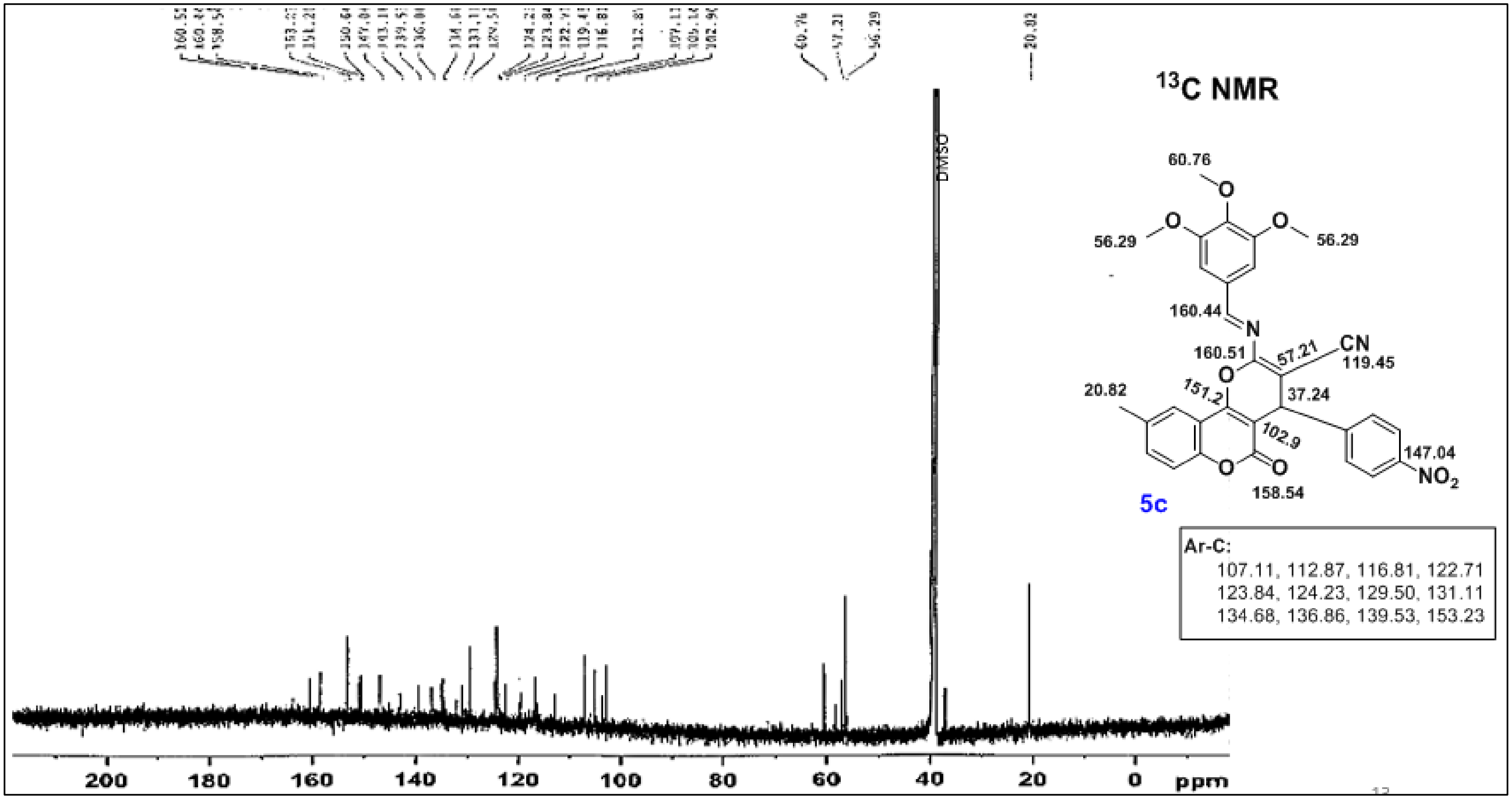

Figure S8: ^{13}C-NMR spectrum of compound **5c** in DMSO-d_6 (176 MHz).

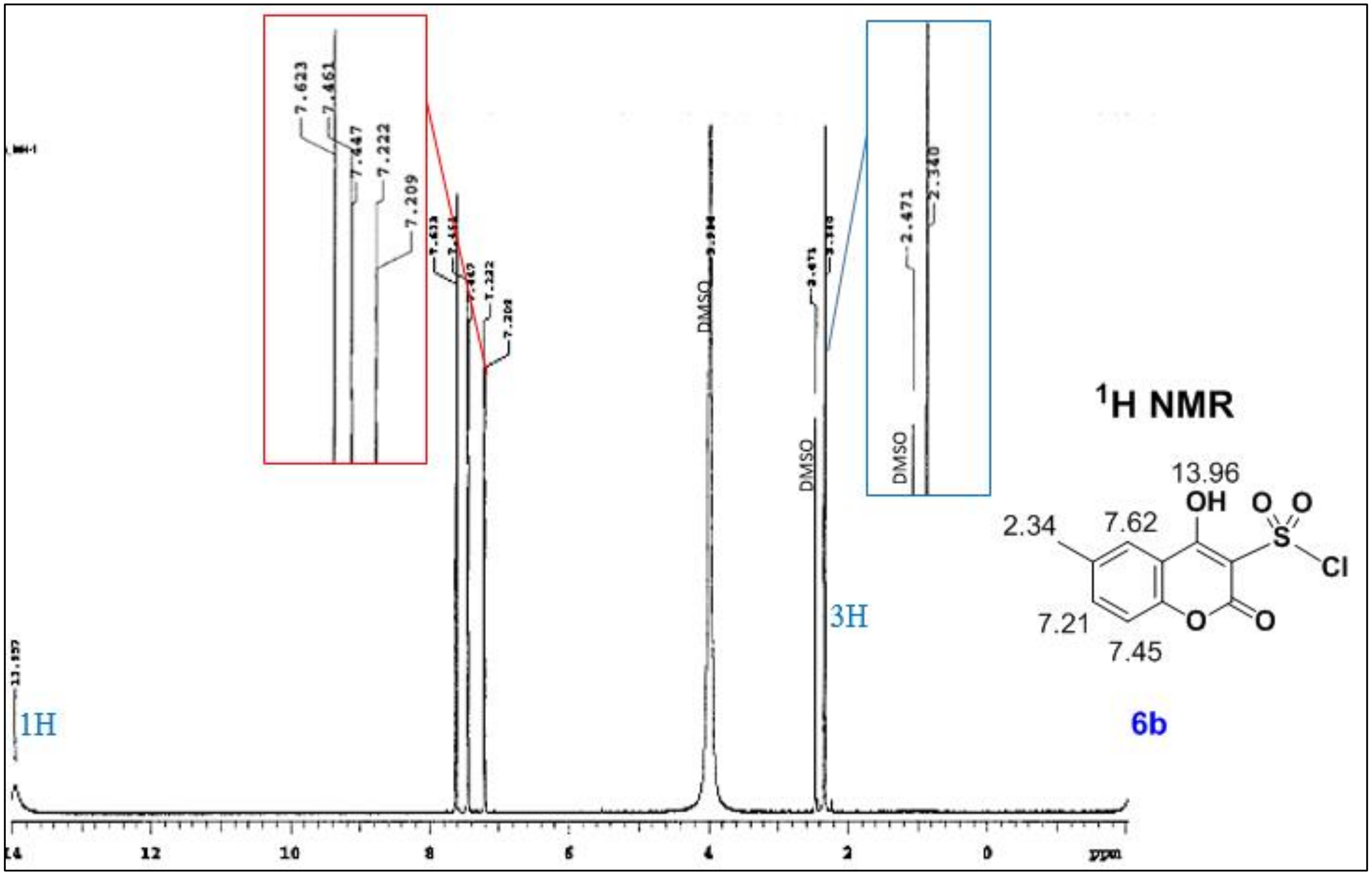

Figure S9: ^{1}H-NMR spectrum of compound **6b** in DMSO-d_6 (600 MHz).

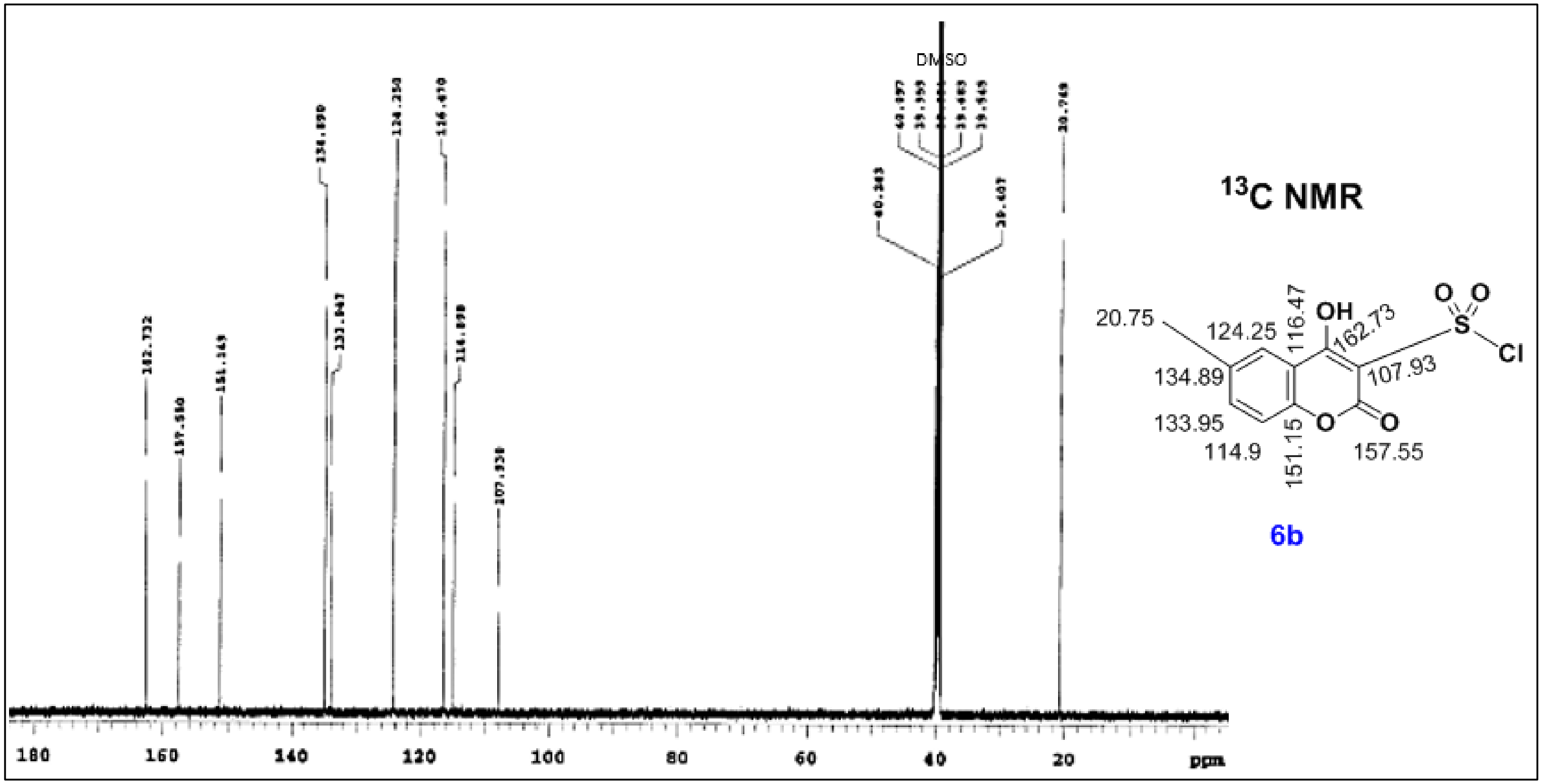

Figure S10a: ^{13}C-NMR spectrum of compound **6b** in DMSO-d$_6$ (154 MHz).

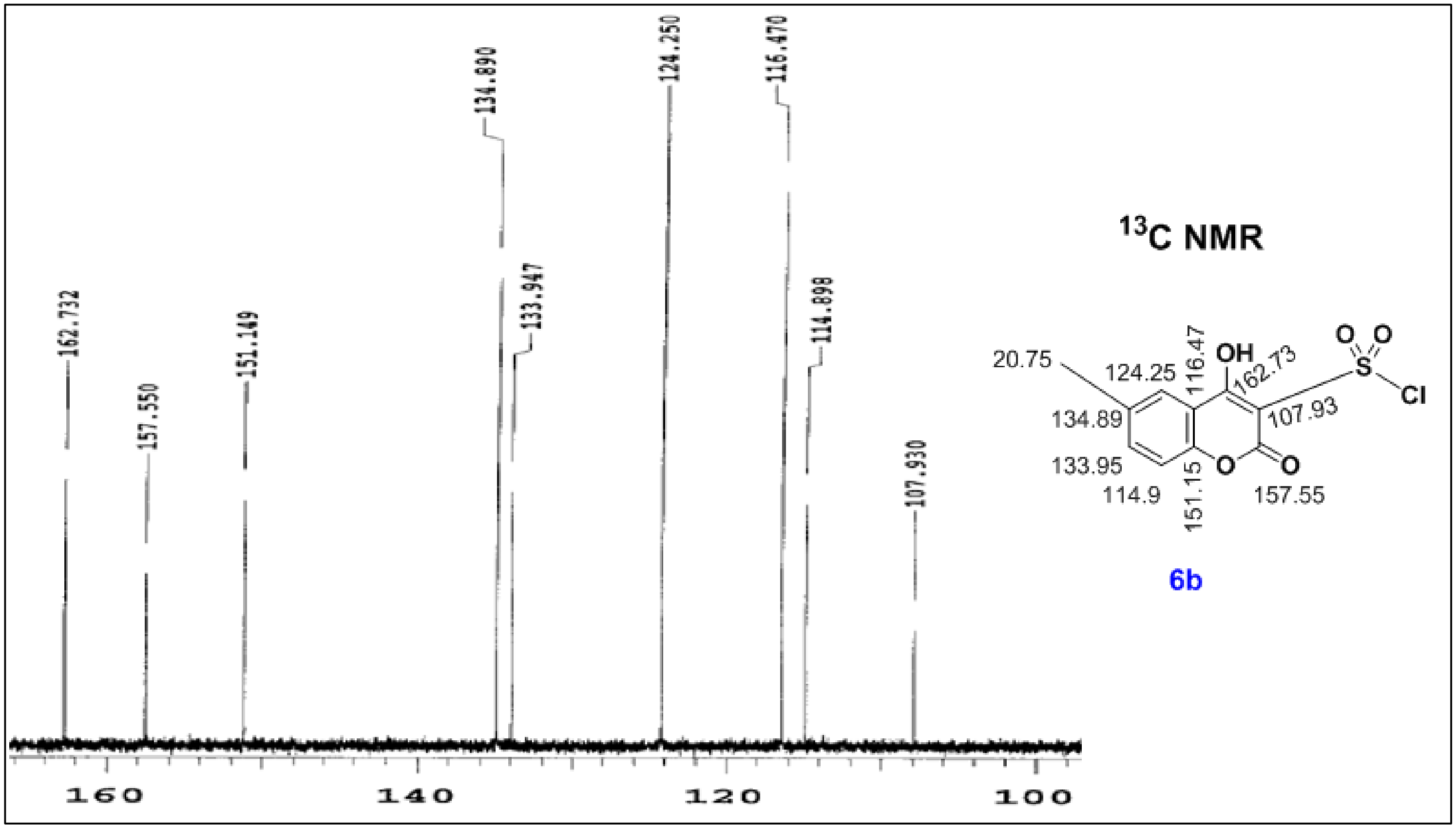

Figure S10b: ^{13}C-NMR spectrum of compound **6b** in DMSO-d_6 (154 MHz).

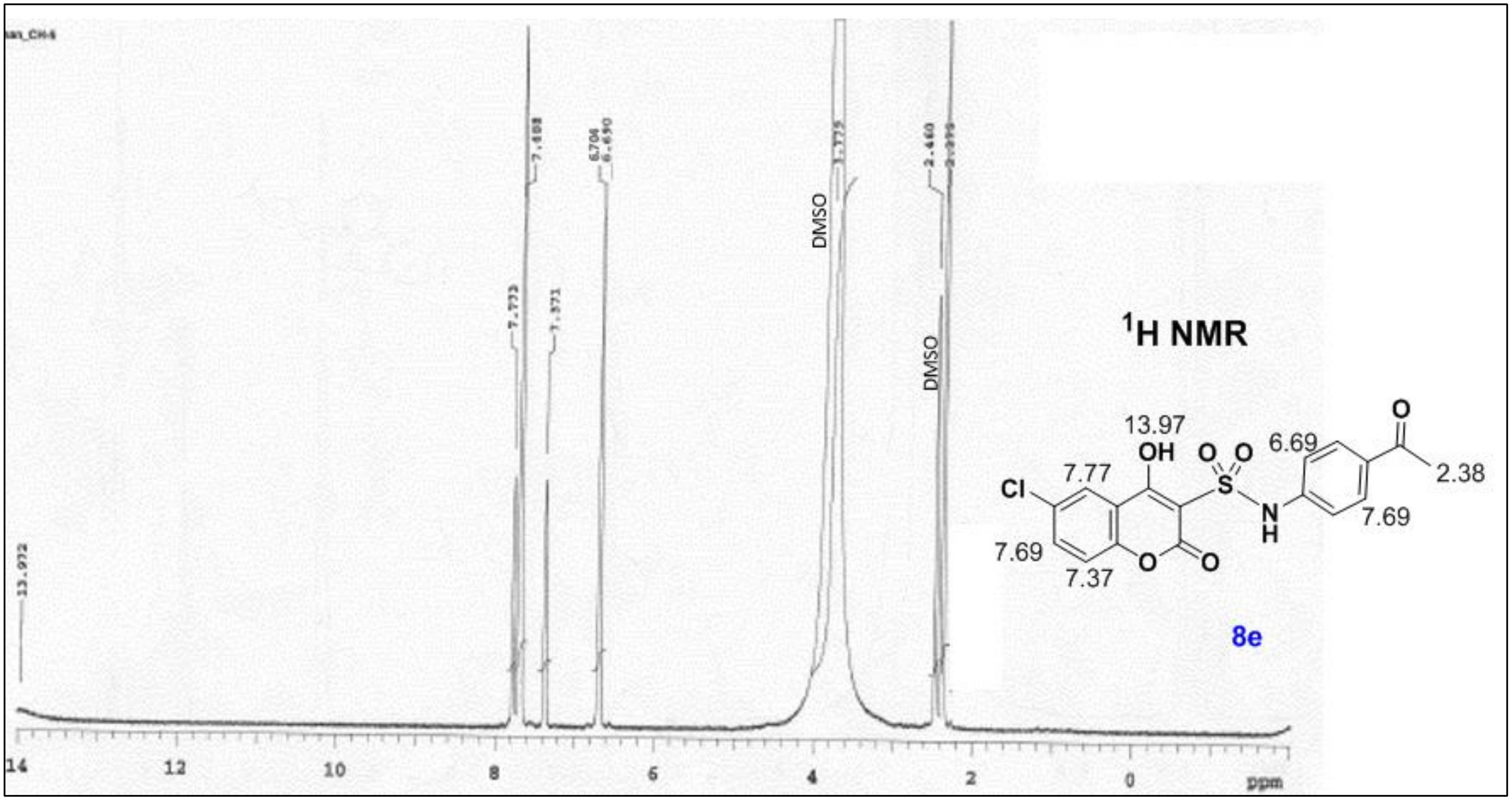

Figure S11: ^{1}H-NMR spectrum of compound **8e** in DMSO-d_6 (600 MHz).

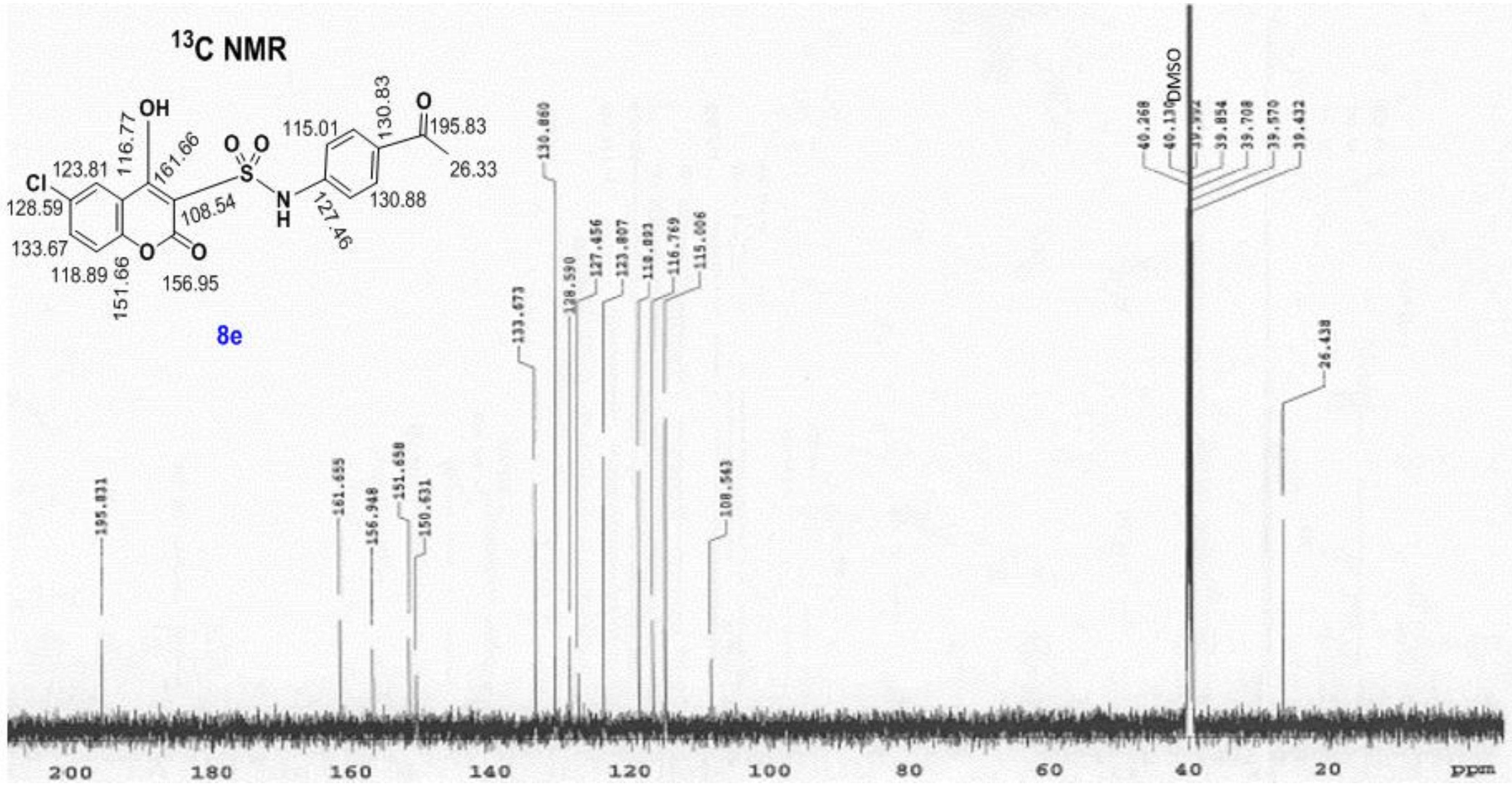

Figure S12: ^{13}C-NMR spectrum of compound **8e** in DMSO-d_6 (154 MHz).

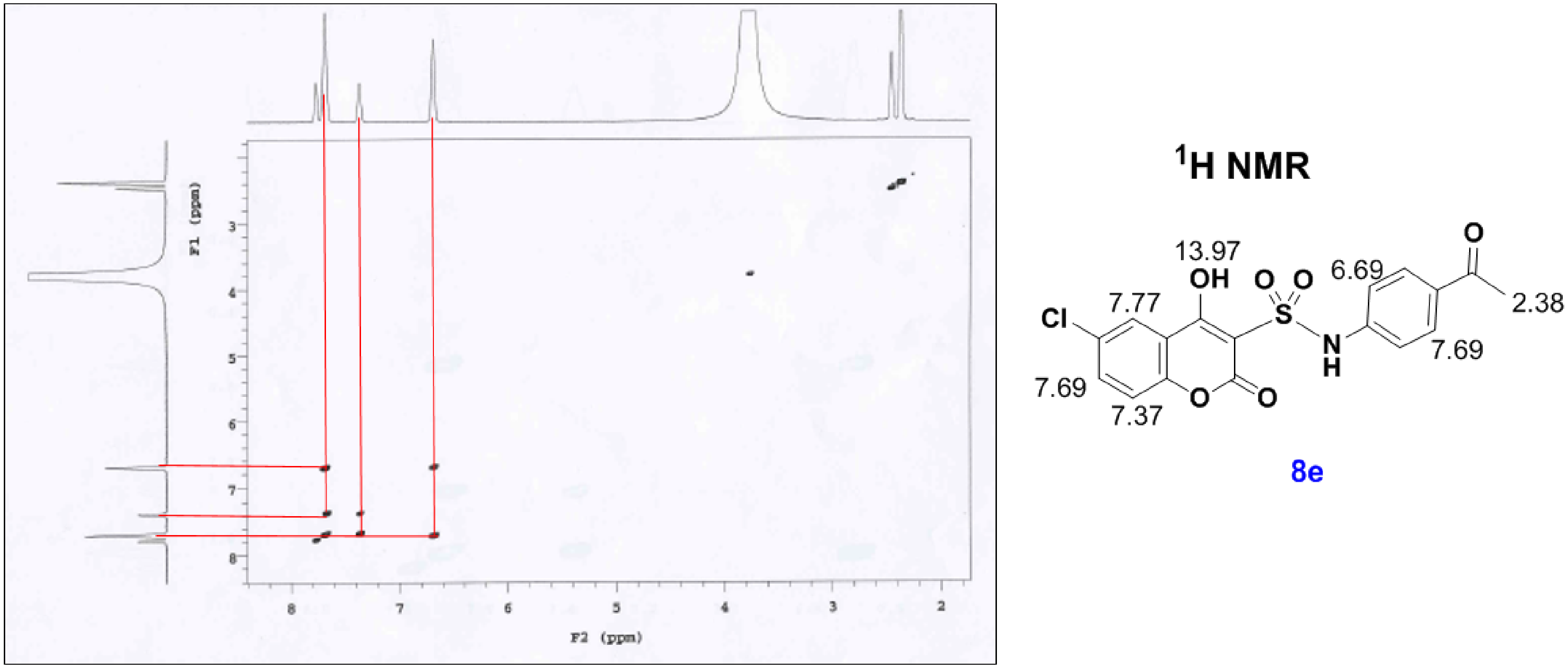

Figure S13: ^{1}H-^{1}H-Homonuclear COSY NMR spectrum of compound **8e** in DMSO-d_6 (600 MHz).

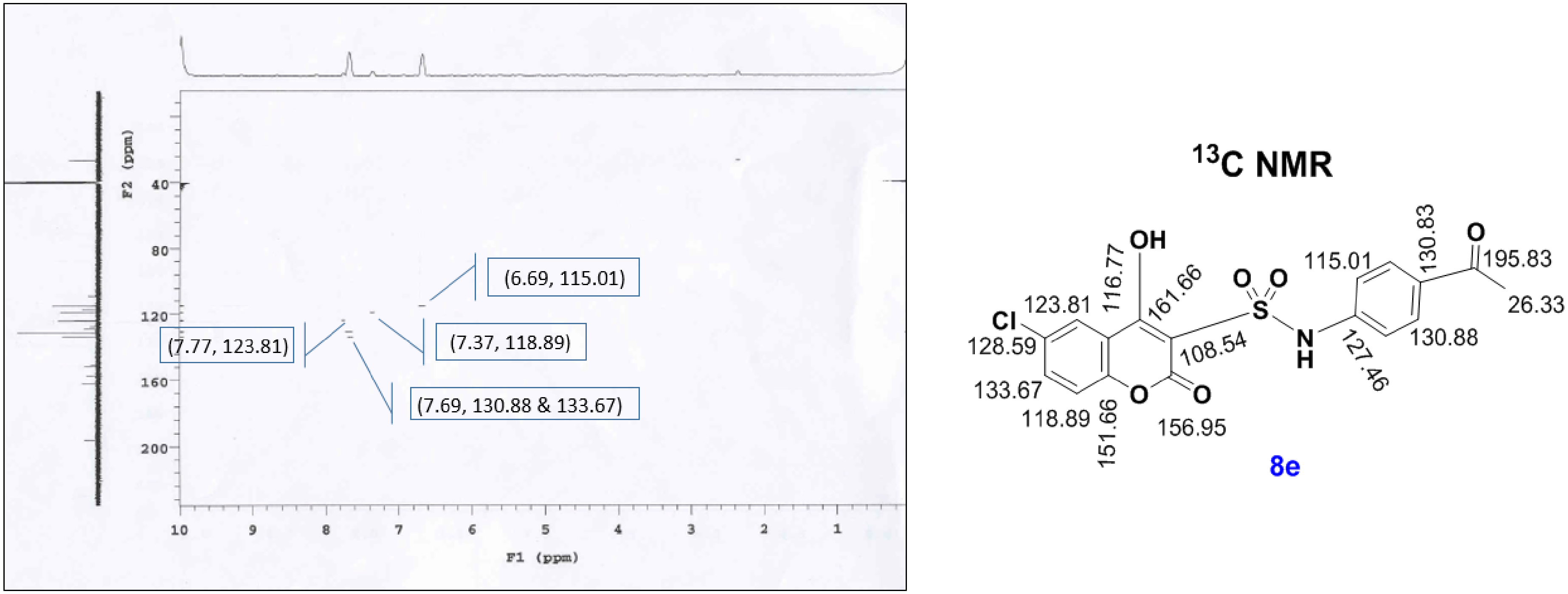

Figure S14: ^{1}H-^{13}C-Heteronuclear COSY NMR spectrum of compound **8e** in DMSO-d_6 (600/154 MHz).

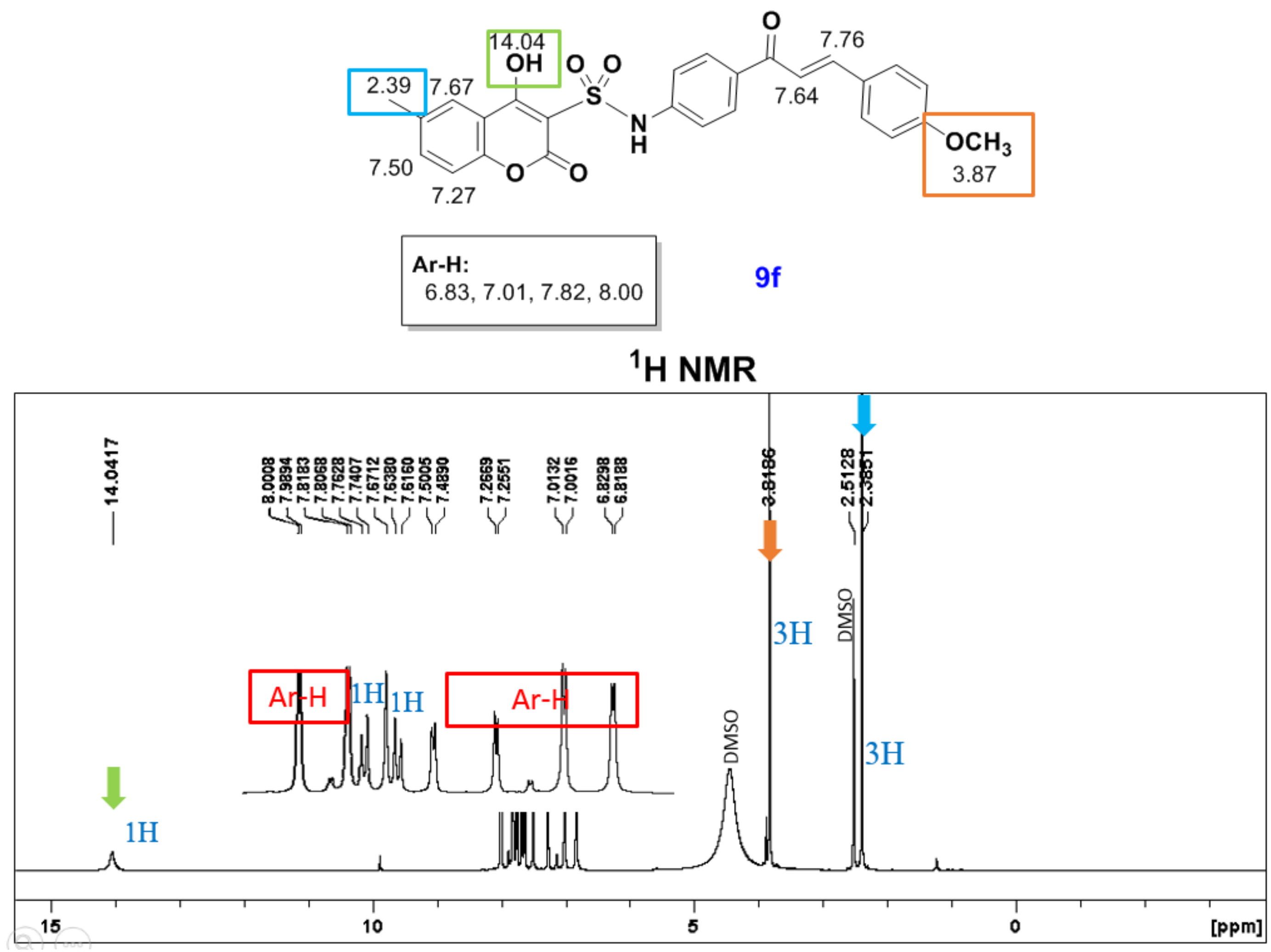

Figure S15: ^{1}H-NMR spectrum of compound **9f** in DMSO-d$_6$ (700 MHz).

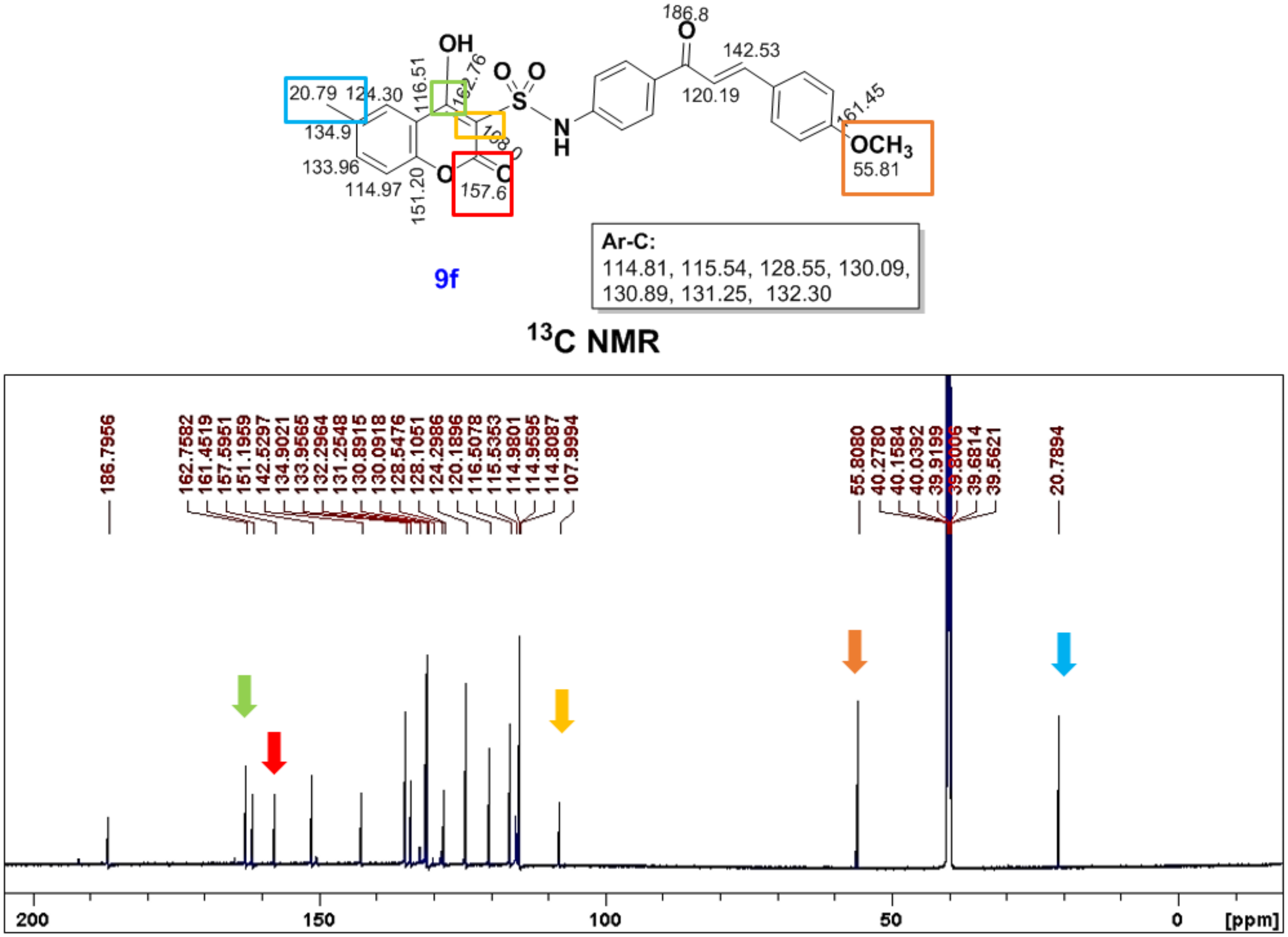

Figure S16: ^{13}C-NMR spectrum of compound **9f** in DMSO-d_6 (176 MHz).

Table S1: X-ray Crystallographic data of Compound **2a**: Bond precision: C-C = 0.0020 Å, Wavelength=0.71073, Cell: a=6.1640(4) b=20.1792(14) c=13.4613(9) ⊚=90, ⊚ =95.956(2), ⊚ =90, Temperature: 123 K, Correction method= # Reported T Limits: Tmin=0.721 Tmax=0.746, AbsCorr = MULTI-SCAN, Data completeness= 1.000, Theta(max)= 29.998, R(reflections)= 0.0478(3442), wR2(reflections)= 0.1142(4853), S = 1.017, Npar= 262.

	**Calculated**	**Reported**
**Volume**	1665.34(19)	1665.34(19)
**Space group**	P 21/n	P 21/n
**Hall group**	-P 2yn	-P 2yn
**Moiety formula**	$C_{20} H_{13} N_3 O_5$	$C_{20} H_{13} N_3 O_5$
**Sum formula**	$C_{20} H_{13} N_3 O_5$	$C_{20} H_{13} N_3 O_5$
**Mr**	375.33	375.33
**Dx,g cm-3**	1.497	1.497
**Z**	4	4
**Mu (mm-1)**	0.110	0.110
**F000**	776.0	776.0
**F000′**	776.41	
**h,k,lmax**	8,28,18	8,28,18
**Nref**	4855	4853
**Tmin,Tmax**	0.991,0.995	0.721,0.746
**Tmin′**	0.970	
